# Supplementary material for: Onset of Action of Selected Second-Generation Antipsychotics (Pines)–A Systematic Review and Meta-Analyses
Source: Biomedicines. 2022 Dec 29;11(1):82. doi: 10.3390/biomedicines11010082 (PMC9855925; doi:10.3390/biomedicines11010082)
Supplement: Supplementary file 1 [file biomedicines-11-00082-s001.zip › Supplementary data_30112022_final.pdf]

Supplementary data

Onset of action of selected Second-Generation Antipsychotics (the pines) A Systematic Review and Meta-Analyses

R. Meyer, K. Skov, I.K. Dhillon, E. Olsson, N. Graudal, L. Baandrup, G. Jürgens

Index

Supplemental 1 – PRISMA-checklist **3**

Supplemental 2 – Search strategies **6**

MEDLINE *6*

EMBASE *6*

Cochrane Central Register of Controlled Trials (CENTRAL) *6*

PsycINFO *6*

Supplemental 3 – Study characteristics **7**

Supplemental 4 – Risk of Bias Assessment **31**

Supplemental 5 – Forest plots **33**

Pooled meta-analyses for immediate release and transdermal drug delivery antipsychotics *33*

Asenapine *36*

Asenapine, transdermal *37*

Olanzapine *38*

Quetiapine *40*

Zotepine *41*

Olanzapine, Long-acting Injections *42*

Quetiapine, Extended Release *44*

Supplemental 6 – Sensitivity analysis, sequentially data only **45**

Asenapine sequentially data only *45*

Olanzapine sequentially data only *46*

Quetiapine sequentially data only *47*

Zotepine sequentially data only *49*

Supplemental 7 – Sensitivity analyses, enriched vs non-enriched **51**

Asenapine, enriched and non-enriched *51*

Olanzapine, enriched and non-enriched *51*
Quetiapine, enriched and non-enriched *52*
Quetiapine XR, enriched and non-enriched *53*

Supplemental 8 – Sensitivity analyses, withdrawal of placebo-responders 54
Olanzapine, without studies reporting withdrawal of placebo-responders *54*
Quetiapine, without studies reporting withdrawal of placebo-responders *54*
Zotepine, without studies reporting withdrawal of placebo-responders *55*

References 56

Supplemental 1 – PRISMA-checklist [1]

Section and Topic	Item #	Checklist item	Location where item is reported
**TITLE**			
Title	1	Identify the report as a systematic review.	Title
**ABSTRACT**			
Abstract	2	See the PRISMA 2020 for Abstracts checklist.	Abstract
**INTRODUCTION**			
Rationale	3	Describe the rationale for the review in the context of existing knowledge.	1
Objectives	4	Provide an explicit statement of the objective(s) or question(s) the review addresses.	1
**METHODS**			
Eligibility criteria	5	Specify the inclusion and exclusion criteria for the review and how studies were grouped for the syntheses.	2.1-2.4
Information sources	6	Specify all databases, registers, websites, organisations, reference lists and other sources searched or consulted to identify studies. Specify the date when each source was last searched or consulted.	2.5
Search strategy	7	Present the full search strategies for all databases, registers and websites, including any filters and limits used.	The supplementary data file
Selection process	8	Specify the methods used to decide whether a study met the inclusion criteria of the review, including how many reviewers screened each record and each report retrieved, whether they worked independently, and if applicable, details of automation tools used in the process.	2.6
Data collection process	9	Specify the methods used to collect data from reports, including how many reviewers collected data from each report, whether they worked independently, any processes for obtaining or confirming data from study investigators, and if applicable, details of automation tools used in the process.	2.7
Data items	10a	List and define all outcomes for which data were sought. Specify whether all results that were compatible with each outcome domain in each study were sought (e.g. for all measures, time points, analyses), and if not, the methods used to decide which results to collect.	2.7
	10b	List and define all other variables for which data were sought (e.g. participant and intervention characteristics, funding sources). Describe any assumptions made about any missing or unclear information.	2.7
Study risk of bias assessment	11	Specify the methods used to assess risk of bias in the included studies, including details of the tool(s) used, how many reviewers assessed each study and whether they worked independently, and if applicable, details of automation tools used in the process.	2.8
Effect measures	12	Specify for each outcome the effect measure(s) (e.g. risk ratio, mean difference) used in the synthesis or presentation of results.	2.9

Section and Topic	Item #	Checklist item	Location where item is reported
Synthesis methods	13a	Describe the processes used to decide which studies were eligible for each synthesis (e.g. tabulating the study intervention characteristics and comparing against the planned groups for each synthesis (item #5)).	2.10
	13b	Describe any methods required to prepare the data for presentation or synthesis, such as handling of missing summary statistics, or data conversions.	2.7
	13c	Describe any methods used to tabulate or visually display results of individual studies and syntheses.	2.10
	13d	Describe any methods used to synthesize results and provide a rationale for the choice(s). If meta-analysis was performed, describe the model(s), method(s) to identify the presence and extent of statistical heterogeneity, and software package(s) used.	2.10
	13e	Describe any methods used to explore possible causes of heterogeneity among study results (e.g. subgroup analysis, meta-regression).	2.11
	13f	Describe any sensitivity analyses conducted to assess robustness of the synthesized results.	2.11
Reporting bias assessment	14	Describe any methods used to assess risk of bias due to missing results in a synthesis (arising from reporting biases).	NA
Certainty assessment	15	Describe any methods used to assess certainty (or confidence) in the body of evidence for an outcome.	NA
**RESULTS**			
Study selection	16a	Describe the results of the search and selection process, from the number of records identified in the search to the number of studies included in the review, ideally using a flow diagram.	Figure 2
	16b	Cite studies that might appear to meet the inclusion criteria, but which were excluded, and explain why they were excluded.	Figure 2
Study characteristics	17	Cite each included study and present its characteristics.	The supplementary data file
Risk of bias in studies	18	Present assessments of risk of bias for each included study.	The supplementary data file
Results of individual studies	19	For all outcomes, present, for each study: (a) summary statistics for each group (where appropriate) and (b) an effect estimate and its precision (e.g. confidence/credible interval), ideally using structured tables or plots.	The supplementary data file
Results of syntheses	20a	For each synthesis, briefly summarise the characteristics and risk of bias among contributing studies.	NA

Section and Topic	Item #	Checklist item	Location where item is reported
	20b	Present results of all statistical syntheses conducted. If meta-analysis was done, present for each the summary estimate and its precision (e.g. confidence/credible interval) and measures of statistical heterogeneity. If comparing groups, describe the direction of the effect.	3.4 + The supplementary data file
	20c	Present results of all investigations of possible causes of heterogeneity among study results.	3.5
	20d	Present results of all sensitivity analyses conducted to assess the robustness of the synthesized results.	3.5
Reporting biases	21	Present assessments of risk of bias due to missing results (arising from reporting biases) for each synthesis assessed.	NA
Certainty of evidence	22	Present assessments of certainty (or confidence) in the body of evidence for each outcome assessed.	NA
**DISCUSSION**			
Discussion	23a	Provide a general interpretation of the results in the context of other evidence.	4
	23b	Discuss any limitations of the evidence included in the review.	4
	23c	Discuss any limitations of the review processes used.	NA
	23d	Discuss implications of the results for practice, policy, and future research.	4
**OTHER INFORMATION**			
Registration and protocol	24a	Provide registration information for the review, including register name and registration number, or state that the review was not registered.	2
	24b	Indicate where the review protocol can be accessed, or state that a protocol was not prepared.	2
	24c	Describe and explain any amendments to information provided at registration or in the protocol.	NA
Support	25	Describe sources of financial or non-financial support for the review, and the role of the funders or sponsors in the review.	Funding
Competing interests	26	Declare any competing interests of review authors.	Conflicts of interest
Availability of data, code and other materials	27	Report which of the following are publicly available and where they can be found: template data collection forms; data extracted from included studies; data used for all analyses; analytic code; any other materials used in the review.	NA

Supplemental 2 – Search strategies

MEDLINE

Performed 16.08.2022

https://ovidsp.ovid.com/ovidweb.cgi?T=JS&CSC=Y&NEWS=N&PAGE=titles&SEARCHNAME=Pine-antipsychotics+and+schizophrenia+or+psychosis+and+RCT+and+placebo+-+MEDLINE-PubMed&SEARCHTYPE=ps&SEARCHLEVEL=pin&D=ppezv

EMBASE

Performed 16.08.2022

https://ovidsp.ovid.com/ovidweb.cgi?T=JS&CSC=Y&NEWS=N&PAGE=titles&SEARCHNAME=Pine-antipsychotics+and+schizophrenia+or+psychosis+and+RCT+and+placebo+-+Embase&SEARCHTYPE=ps&SEARCHLEVEL=pin&D=oemezd

Cochrane Central Register of Controlled Trials (CENTRAL)

Performed 16.08.2022

https://www.cochranelibrary.com/advanced-search/search-manager?search=7010441

PsycINFO

Performed 16.08.2022

https://ovidsp.ovid.com/ovidweb.cgi?T=JS&CSC=Y&NEWS=N&PAGE=titles&SEARCHNAME=Pine-antipsychotics+and+schizophrenia+or+psychosis+and+RCT+and+placebo+-+PsychInfo&SEARCHTYPE=ps&SEARCHLEVEL=pin&D=psyh

Supplemental 3 – Study characteristics

Study characteristics of included studies distributed by drug.

SUBLINGUAL ASENAPINE				
**Study ID**	**Design**	**Population**	**Intervention**	**Outcome measure**
Potkin et al. 2007[2]	RCT, DB, PLB-controlled<br>3-arm study<br><br>Duration: 6 weeks<br><br>Run-in period:<br>- Pre baseline washout: yes (≥ 3 days for current antipsychotic medication and ≥ 5 days for mood stabilizers)<br>- Pre study trial-drug phase: No<br><br>Inclusion criteria:<br>- DSM-IV: Schizophrenia (disorganized, paranoid, catatonic or undifferentiated)<br>- Acute exacerbation<br>- CGI-S ≥ 4<br>- PANSS ≥ 60 and a score of ≥ 4 on ≥ 2 of the items: Delusions, conceptual disorganization, hallucinatory behavior, grandiosity, suspiciousness<br>- If received depot neuroleptics ≥ 1 month between last injection and the first dose of trial medication.<br><br>Substance abuse allowed: No<br>Hospitalized the first 3 weeks<br><br>Must meet inclusion criteria after washout: NSPEC	Randomized, N= 182<br><br>Age, mean (range), years:<br>- Asenapine: 38 (21-70)<br>- Risperidone: 43 (22-61)<br>- PLB: 42 (22-68)<br><br>Sex, male: 72.8%<br><br>Enriched population: Yes<br><br>PLB-responders withdrawn: Yes, PANSS at BL required to be ≥ 80% of that of prior visits.<br><br>Those who were >75% adherent during the wash out period was randomized.<br><br>PANSS-total mean at BL:<br>- Asenapine: 96.48<br>- Risperidone: 92.18<br>- PLB: 92.43<br>- Total 93.7<br><br>Completed the study: Asenapine 46%, Risperidone 42%, PLB 34%<br><br>LOCF/ITT-population: Yes	Sublingual Asenapine 10mg/d (n=58) vs Oral Risperidone 6 mg/d (n=56) vs<br>PLB (n=60)<br><br>Administration: B.i.d.<br><br>Dose titration: 1 mg at day 1, 2 mg at day 2, 3 mg at day, 4 mg at day 4 and 5 mg day 3-42<br><br>Double-dummy design:<br>Asenapine treated patients also received oral PLB.<br>Risperidone treated patients also received sublingual PLB.<br>PLB treated patients received oral and sublingual PLB.<br><br>Fixed doses: NSPEC<br><br>Allowed supplemental medication:<br>- For sleep induction: Zolpidem tartrate ≤ 10 mg/d, Zaleplon ≤ 20 mg/d or chloral hydrate ≤ 3000 mg/d.<br>- For agitation: benzodiazepines at daily doses equivalent to Lorazepam ≤ 10 mg/d.<br>- Anticholinergics for newly emergent EPS.	Primary:<br>- PANSS-total<br><br>Secondary:<br>- CGI-S<br>- PANSS-positive subscale<br>- PANSS-negative subscale<br>- PANSS general psychopathology<br><br>Follow up (days): 7, 14, 21, 28, 35, 42<br><br>Interrater reliability: Raters required to have ≥ 2 years of experience in performing clinical evaluations of patients with schizophrenia. No κ-value.

Kane et al. 2010[3]	RCT, DB, PLB-controlled<br>4-arm study<br><br>Duration: 6 weeks<br><br>Run-in period:<br>- Pre baseline washout: Yes (3 days)<br>- Pre study trial-drug phase: No<br><br>Inclusion criteria:<br>- DSM-IV: Schizophrenia<br>- Acute exacerbation<br>- PANSS ≥ 60 and score of ≥4 on ≥2 PANSS-Positive subscale items: Delusions, conceptual disorganization, hallucinatory behavior, grandiosity, suspiciousness<br>- CGI-S ≥4<br><br>Substance abuse allowed: No<br>Hospitalized the first 2 weeks<br><br>Must meet inclusion criteria after washout: Yes	Randomized, N= 458<br><br>Age, mean range, years: 37-40 across the 4 treatment groups<br><br>Sex, male: 52-68%<br><br>Enriched population: No<br><br>PLB-responders withdrawn: Yes, patient excluded if ≥ 20% decrease in PANSS-total from screening to BL.<br><br>PANSS-total mean at BL: NSPEC<br><br>Completed the study: 61% (272)<br><br>LOCF/ITT-population: Yes	Sublingual Asenapine at two dosages (n=214) vs Haloperidol 4 mg/d (n=112) vs PLB (n=122)<br><br>Administration: B.i.d.<br>Dosage groups,<br>- Sublingual Asenapine 10mg/d (n=109)<br>- Sublingual Asenapine 20mg/d (n=105)<br><br>Dose titration: NSPEC<br><br>Fixed doses: Yes<br><br>Allowed supplemental medication: NSPEC	Primary:<br>- PANSS-total<br><br>Secondary:<br>- PANSS subscale scores<br>- PANSS Marder factors<br>- CGI-S<br>- CGI-I<br>- CDSS<br><br>Follow up (days): 4, 7, 14, 21, 28, 35, 42<br><br>Interrater reliability: NSPEC. No κ-value.
Kinoshita et al. 2016[4]	RCT, DB, PLB-controlled<br>3-arm study<br><br>Duration: 6 weeks<br><br>Run-in period:<br>- Pre baseline washout: Yes (3-7 days)<br>- Pre study trial-drug phase: No<br><br>Inclusion criteria:<br>- DSM-IV: Schizophrenia (paranoid, disorganized, catatonic, undifferentiated)<br>- Acute exacerbation ≤ 2 months duration<br>- PANSS ≥ 60 and score of ≥4 on ≥2 PANSS-Positive subscale items: Delusions, conceptual disorganization, hallucinatory behavior, grandiosity, suspiciousness<br>- CGI ≥ 4	Randomized, N= 532<br><br>Age, mean (SD), years: 41.42 (11.45)<br><br>Sex, male: 48.1%<br><br>Enriched population: Yes<br><br>PLB-responders withdrawn: Yes, patient excluded if ≥ 20% decrease in PANSS-total from screening to BL<br><br>PANSS-total mean (SD) at BL:<br>- Asenapine 10 mg: 94.15 (17.97)<br>- Asenapine 20 mg: 92.74 (17.34)	Sublingual Asenapine at two dosages (n=351) vs PLB (n=174)<br><br>Administration: B.i.d.<br>Dosage groups,<br>- Sublingual Asenapine 10 mg/d (n=173)<br>- Sublingual Asenapine 20 mg/d (n=178)<br><br>Dose titration: NSPEC<br><br>Fixed doses: Yes<br><br>Allowed supplemental medication:<br>- Agitation and anxiety: Short-acting benzodiazepines<br>- Lorazepam<br>- EPS medication if EPS worsened or appeared.	Primary:<br>- PANSS-total<br><br>Secondary:<br>- CGI<br><br>Follow up (days): 4, 7, 14, 21, 28, 35, 42<br><br>Interrater reliability: Performed by an investigator or trained rater. Each patient was rated by the same rater for assessments. No κ-value.

	- If received depot neuroleptics >3 month must have occurred between last injection and randomization.<br><br>Substance abuse allowed: No<br>Hospitalized at least the first 3 weeks<br><br>Must meet inclusion criteria after washout: Yes	- PLB: 94.51 (17.26)<br>- Total: 93.79 (17.51)<br><br>Completed the study: 57.2% (303)<br><br>LOCF/ITT-population: Yes		
Landbloom et al. 2017[5]	RCT, DB, PLB-controlled<br>4-arm study<br><br>Duration: 6 weeks<br><br>Run-in period:<br>- Pre baseline washout: NSPEC<br>- Pre study trial-drug phase: No<br><br>Inclusion criteria:<br>- DSM-IV: Schizophrenia (paranoid, disorganized, undifferentiated type)<br>- Acute exacerbation ≤ 8 weeks<br>- PANSS ≥ 70 and score of ≥4 on ≥2 PANSS-Positive subscale items<br>- CGI ≥4<br><br>Substance abuse allowed: No<br>Hospitalized at least 3 weeks.<br><br>Must meet inclusion criteria after washout: Yes	Randomized, N= 360<br><br>Age, mean, years: 40.6<br><br>Sex, male: 58.5%<br><br>Enriched population: Yes<br><br>PLB-responders withdrawn: Yes, patient excluded if ≥ 20% decrease in PANSS-total from screening to BL.<br><br>PANSS-total mean (SD) at BL:<br>- Asenapine 5 mg: 93.3 (11.10)<br>- Asenapine 10 mg: 95.8 (13.77)<br>- Olanzapine: 92.7 (10.47)<br>- PLB: 93.4 (11.16)<br>- Total: 94.1 (11.97)<br><br>Completed the study: 60%<br><br>LOCF/ITT-population: Yes	Sublingual Asenapine in two dosages (n=207) vs Olanzapine 15 mg (n=45) vs. PLB (n=99)<br><br>Administration: Once daily<br>Dosage groups,<br>- Sublingual Asenapine 5 mg/d (n=96)<br>- Sublingual Asenapine 10 mg/d (n=111)<br><br>Dose titration: Olanzapine group received 10mg the first week.<br><br>Fixed doses: Yes<br><br>Allowed supplemental medication:<br>NSPEC	Primary:<br>- PANSS-total<br><br>Secondary:<br>- CGI-S<br><br>Follow up (days): 42<br><br>Interrater reliability:<br>NSPEC. No κ-value.

TRANSDERMAL ASENAPINE				
**Study ID**	**Design**	**Population**	**Intervention**	**Outcome measure**
Citrome et al. 2020[6]	RCT, DB, PLB-controlled<br>3- arm study<br><br>Duration: 6 weeks<br><br>Run-in period:<br>- Pre baseline washout: Yes (3-14 days)<br>- Pre study trial-drug phase: No<br><br>Inclusion criteria:<br>- DSM-IV: Schizophrenia<br>- Acute exacerbation<br>- PANSS ≥ 80 and a score of ≥ 4 in ≥ 2 of the items: Delusions, hallucinatory behavior, conceptual disorganization, unusual thought content<br>- CGI-S ≥ 4<br>- Disease duration: > 6 months<br><br>Substance abuse allowed: No<br>Hospitalized for during the treatment period<br><br>Must meet inclusion criteria after washout: NSPEC	Randomized, N= 617<br><br>Age, mean, years: 42,0<br><br>Sex, male: 60,6%<br><br>Enriched population: Yes<br><br>PLB-responders withdrawn: Yes, patient excluded if ≥ 20% decrease in PANSS-total from screening to BL.<br><br>PANSS-total mean at BL:<br>- HP-3070 3.8mg/24hours: 97.0 (9.74)<br>- HP-3070 7.6mg/24hours: 95.6 (8.68)<br>- PLB: 97.3 (10.05)<br><br>Completed the study: 79%<br><br>LOCF/ITT-population: No, solely MMRM was used	HP-3070 at two dosages (n=411) vs PLB (n=206)<br><br>Administration: Once daily (evening)<br>Dosage groups,<br>- HP-3070 3.8mg/24hours (n=205)<br>- HP-3070 7.6mg/24hours (n=206)<br><br>Fixed doses: Yes<br><br>Allowed supplemental medication: NSPEC	Primary:<br>- PANSS-total<br><br>Secondary:<br>- CGI-S<br>- CGI-I<br><br>Follow up (days): 7, 14, 21, 28, 35, 42<br><br>Interrater reliability: NSPEC. No κ-value.
ORAL OLANZAPINE				
**Study ID**	**Design**	**Population**	**Intervention**	**Outcome measure**
Lecrubier et al 2006[7]	RCT, DB, PLB-controlled<br>4-arm study<br><br>Duration: 6 months<br>- Optional DB extension period for responders to the 6 months treatment period.	Randomized, N= 245<br><br>Age, mean (SD), years:<br>- Olanzapine 5 mg: 38.1 (11.1)<br>- Olanzapine 20 mg: 36.4 (10.4)<br>- Amisulpride: 37.8 (11.6)<br>- PLB: 38.2 (9.0)	Olanzapine in two dosages (n=140) vs Amisulpride 150 mg (n=70) vs PLB (n=34)<br><br>Administration: Once daily (evening)<br>Dosage groups,<br>- Olanzapine 5 mg (n=70)	Primary:<br>- SANS<br><br>Secondary:<br>- PANSS-total<br>- PDS<br>- BPRS

	Run-in period:<br>- Pre baseline washout: Yes (2-9 days)<br>- Pre study trial-drug phase: No<br><br>Inclusion criteria:<br>- DSM-IV: Schizophrenia, residual type, disorganized or catatonic<br>- Disease duration: At least 1 year<br>- SANS ≥ 10<br>- PANSS ≤4 on the items: hallucinations and delusions<br><br>Substance abuse allowed: No<br>Hospitalization: 60.5% at inclusion<br><br>Must meet inclusion criteria after washout: Yes	Sex, male: 68%<br><br>Enriched population: yes<br><br>PLB-responders withdrawn: NSPEC<br><br>PANSS-total mean at BL:<br>- Olanzapine 5 mg: 67.5 (17.3)<br>- Olanzapine 20 mg: 65.4 (15.4)<br>- Amisulpride: 67.4 (14.8)<br>- PLB: 67.9 (14.4)<br><br>Completed the study: 41.3%<br><br>LOCF/ITT-population: Yes	- Olanzapine 20 mg (n=70)<br><br>Dose titration: NSPEC<br><br>Fixed doses: Yes<br><br>Allowed supplemental medication:<br>- Benzodiazepines: Chronic users, dose up to 60 mg of diazepam equivalent. Non-chronic users could be treated with diazepam 60 mg/d for a cumulative time administration of 60 days.<br>- Benztropine or biperiden for EPS at a total of 4 mg/day.	- CGI<br>- PGI<br><br>Follow up (days): 7, 14, 21, 28, 35, 42 and then every 4 weeks<br><br>Interrater reliability: Investigators trained by videotapes. Rating should be within 1.5 SD otherwise proposed further training or withdraw from the study.
Meltzer et al. 2011[8]	RCT, DB, PLB-controlled<br>4-arm study<br><br>Duration: 6 weeks<br><br>Run-in period:<br>- Pre baseline washout: No<br>- Pre study trial-drug phase: No<br><br>Inclusion criteria:<br>- DSM-IV: Schizophrenia<br>- Hospitalized ≤ 2 weeks for an acute exacerbation<br>- CGI-S ≥4<br>- PANSS ≥ 80 and a score of ≥ 4 on the items: Delusions, hallucinatory behavior and conceptual disorganization, unusual thought content, suspiciousness<br>- Disease duration: At least 1 year<br><br>Substance abuse allowed: NSPEC<br>Hospitalized at least 21 days	Randomized, N= 478<br><br>Age, mean (SD), years:<br>- Lurasidone 40 mg: 37.7 (11.0)<br>- Lurasidone 120 mg: 37.9 (11.2)<br>- Olanzapine: 38.3 (10.2)<br>- PLB: 37.0 (11.3)<br><br>Sex, male: 78%<br><br>Enriched population: No<br><br>PLB-responders withdrawn: NSPEC<br><br>PANSS-total mean (SD) at BL:<br>- Lurasidone 40 mg: 96.6 (10.7)<br>- Lurasidone 120 mg: 97.9 (11.3)<br>- Olanzapine: 96.3 (12.2)<br>- PLB: 95.8 (10.8)	Lurasidone at two dosages (n=236) vs Olanzapine 15 mg (n=121) vs PLB (n=114)<br><br>Administration: Once daily (morning)<br><br>Dose titration: The Lurasidone group received the target dose at treatment start. The olanzapine group received 10mg/d on day 1-7 and 15mg/d thereafter.<br><br>Fixed doses: Yes<br><br>Allowed supplemental medication:<br>Limited use of benzodiazepines for anxiety, agitation or insomnia.	Primary:<br>- PANSS-total<br><br>Secondary:<br>- CGI-S<br>- MADRS<br>- PANSS subscales<br><br>Follow up (days): 4, 7, 14, 21, 28, 35, 42<br><br>Interrater reliability: NSPEC. No κ-value.

	Must meet inclusion criteria after washout: Yes	Completed the study: 62.3% (298)<br><br>LOCF/ITT-population: Yes		
Beasley et al. 1996_1[9]	RCT, DB, PLB-controlled<br>5-arm study<br><br>Duration: 6 weeks<br><br>Run-in period:<br>- Pre baseline washout: Yes (4-7 days)<br>- Pre study trial-drug phase: No<br><br>Inclusion criteria:<br>- DSM-III: Schizophrenia<br>- Acute exacerbation<br>- BPRS ≥ 24<br><br>Substance abuse allowed: No<br>Hospitalized for washout and first 2 weeks of treatment<br><br>Must meet inclusion criteria after washout: NSPEC	Randomized, N= 335<br><br>Age, mean (SD), years:<br>- Olanzapine-Low: 36 (10)<br>- Olanzapine-medium: 37 (10)<br>- Olanzapine-high: 36 (10)<br>- Haloperidol: 36 (9)<br>- PLB: 35 (8)<br><br>Sex, male: 87,8%<br><br>Enriched population: No<br><br>PLB-responders withdrawn: Yes, if ≥ 25% decrease in BPRS-score or BPRS-score decreased to <24 during the placebo lead-in phase.<br><br>BPRS-total mean (SD) at BL:<br>- Olanzapine-Low: 41.2 (11.7)<br>- Olanzapine-medium: 42.8 (10.0)<br>- Olanzapine-high: 42.6 (10.9)<br>- Haloperidol: 41.8 (11.4)<br>- PLB: 39.7 (10.5)<br><br>Completed the study (%):<br>- Olanzapine-Low: 41.5<br>- Olanzapine-medium: 40.6<br>- Olanzapine-high: 49.3<br>- Haloperidol: 43.5<br>- PLB: 32.4<br><br>LOCF/ITT-population: Yes	Olanzapine at low, medium and high dosages (n=190) vs Haloperidol (n=68) vs PLB (n=62)<br><br>Administration: Once daily<br>Dosage groups,<br>- Olanzapine 2.5, 5 or 7.5 mg, mean dose: 6.6 (1.4) mg/d (n=63)<br>- Olanzapine 7.5, 10, 12.5 mg, mean dose: 11.6 (1.5) mg/d (n=62)<br>- Olanzapine 12.5, 15, 17.5 mg, mean dose: 16.3 (1.6) mg/d (n=65)<br>- Haloperidol 10, 15, 20 mg, mean dose: 16.4 (4.0) mg/d (n=68)<br><br>Dose titration: The treatment was initiated in the middle dose and could be adjusted if clinically indicated. Upward adjustment could occur after day 4 and at any scheduled visit. Downward adjustment could occur at any time.<br><br>Fixed doses: No<br><br>Allowed supplemental medication:<br>- Lorazepam ≤10 mg/day during the leading period and ≤ 21 days of the DB treatment period.<br>- Benztropine mesylate ≤ 6 mg/day.	Primary:<br>- BPRS-Anchored, 18-items, score 0-6 at each item<br>- SANS<br><br>Secondary:<br>- CGI-S<br><br>Follow up (days): 3, 7, 14, 21, 28, 35, 42<br><br>Interrater reliability: Investigators received training on administration and scoring of BPRS and SANS. No κ-value.

Beasley et al. 1996_2[10]	RCT, DB, PLB-controlled<br>3-arm study<br><br>Duration: 6 weeks<br><br>Run-in period:<br>- Pre baseline washout: Yes (4-7 days)<br>- Pre study trial-drug phase: No<br><br>Inclusion criteria:<br>- DSM-III: Schizophrenia<br>- BPRS ≥ 24<br>- CGI-S ≥ 4<br><br>Substance abuse allowed: No<br>Hospitalized during wash out and the first 2 weeks of DB-treatment<br><br>Must meet inclusion criteria after washout: Yes	Randomized, N= 152<br><br>Age, mean, years: 38<br><br>Sex, male: 72.4%<br><br>Enriched population: No<br><br>PLB-responders withdrawn: Yes, if BPRS improved by ≥ 25% or to a score <24 between screening and BL.<br><br>BPRS-total mean (SD) at BL:<br>- Olanzapine 1 mg/d: 39.6 (11.1)<br>- Olanzapine 10 mg/d: 37.4 (8.5)<br>- PLB: 36.8 (6.9)<br><br>Completed the study: 27%<br><br>LOCF/ITT-population: yes	Olanzapine at two dosages (n=100) vs PLB (n=49)<br><br>Administration: Once daily<br>Dosage groups,<br>- Olanzapine 1 mg/d (n=51)<br>- Olanzapine 10 mg/d (n=49)<br><br>Dose titration: No<br><br>Fixed doses: Yes<br><br>Allowed supplemental medication:<br>- Lorazepam ≤ 10mg/d during lead-in period and maximum 21 days during DB treatment<br>- Benztropine mesylate ≤ 6mg/d	Primary:<br>- PANSS-total<br>- BPRS<br>- PGI<br><br>Follow up days: 7, 14, 21, 28, 35, 42<br><br>Interrater reliability: Raters were trained by videotaped interviews. No κ-value.
Corrigan et al. 2004[11]	RCT, DB, PLB-controlled<br>5-arm study<br><br>Duration: 6 weeks<br><br>Run-in period:<br>- Pre baseline washout: Yes (3-7 days)<br>- Pre study trial-drug phase: No<br><br>Inclusion criteria:<br>- DSM-IV: Schizophrenia (disorganized, catatonic, paranoid, residual, undifferentiated)<br>- PANSS ≥ 60<br><br>Substance abuse allowed: No<br>Hospitalized 4-7 weeks	Randomized, N= 467<br><br>Age, mean (range), years:<br>- Olanzapine: 36.8 (19-61)<br>- PLB: 37.2 (19-59)<br><br>Sex, male: 65.7%<br><br>Enriched population: No<br><br>PLB-responders withdrawn: NSPEC<br><br>PANSS-total mean at BL:<br>- Olanzapine: 24.0<br>- PLB: 23.8	Sonepiprazol at three dosages (n=277) vs Olanzapine 15 mg (n=93) vs PLB (n=85)<br><br>Administration: Once daily (morning)<br><br>Fixed doses: Yes<br><br>Allowed supplemental medication:<br>- Sleep difficulties: Chloral hydrate ≤ 2mg/d.<br>- EPS: anticholinergics.<br>- Acute dystonic symptoms: oral or parenteral benztropine mesylate 1-4 mg, oral benzhexol hydrochloride 1-15 mg/d or oral diphenhydramine 60-200 mg/d.<br>- Sedation: lorazepam ≤ 6mg/d, zolpidem ≤ 10mg/d or flunitrazepam ≤ 2 mg/d only if Chloral hydrate was ineffective and not	Primary:<br>- PANSS-total<br><br>Secondary:<br>- BPRS<br>- CGI-S<br><br>Follow up (days): 0, 7, 14, 21, 28, 35, 42<br><br>Interrater reliability: NSPEC. No κ-value.

	Must meet inclusion criteria after washout: Yes	Completed the study: 71.7%<br><br>LOCF/ITT-population: Yes	administrated within 8 hours of an efficacy assessment.	
Marder et al. 2007[12]	RCT, DB, PLB-controlled<br>4- arm study<br><br>Duration: 6 weeks<br><br>Run-in period:<br>- Pre baseline washout: Yes (3 days)<br>- Pre study trial-drug phase: No<br><br>Inclusion criteria:<br>- DSM-IV: Schizophrenia<br>- Acute exacerbation<br>- PANSS 70-120<br>- Disease duration: At least 1 year<br><br>Substance abuse allowed: No<br>Hospitalized for ≥ 2 weeks<br><br>Must meet inclusion criteria after washout: Yes	Randomized, N= 444<br><br>Age, mean, years: 41.6<br><br>Sex, male: 74%<br><br>Enriched population: Yes<br><br>PLB-responders withdrawn: NSPEC<br><br>PANSS-total mean at BL:<br>- Paliperidone ER 6 mg: 92.3 (12.0)<br>- Paliperidone ER 12 mg: 94.1 (11.4)<br>- Olanzapine: 94.9 (12.4)<br>- PLB: 93.6 (11.7)<br><br>Completed the study: 43%<br><br>LOCF/ITT-population: Yes	Paliperidone ER at two dosages (n=221) vs Olanzapine 10 mg (n=105) vs PLB (n=105)<br><br>Administration: Once daily<br><br>Fixed doses: Yes<br><br>Allowed supplemental medication:<br>- Benzodiazepines at predefined doses for the treatment of agitation, anxiety or sleep difficulties.<br>- Antidepressants, for patients on a stable dosage for 3 months before the study.	Primary:<br>- PANSS-total<br><br>Secondary:<br>- CGI<br><br>Follow up (days): 4, 8, 15, 22, 29, 36, 43<br><br>Interrater reliability: NSPEC. No κ-value.
Schmidt et al. 2012[13]	RCT, DB, PLB-controlled<br>5-arm study<br><br>Duration: 12 weeks<br>- From week 7-12 the PLB-group converted to Olanzapine by ethical reasons<br><br>Run-in period:<br>- Pre baseline washout: No<br>- Pre study trial-drug phase: No<br><br>Inclusion criteria:	Randomized, N= 498<br><br>Age, mean (SD), years: 39.4 (10.91)<br><br>Sex, male: 57%<br><br>Enriched population: Yes<br><br>PLB-responders withdrawn: Not relevant<br><br>PANSS-total mean (SD) at BL:	JNJ37822681 at three dosages (n=300) vs Olanzapine 15 mg (n=93) vs PLB (n=99)<br><br>Administration: Once daily (evening)<br><br>Fixed doses: Yes<br><br>Allowed supplemental medication:<br>- Anti-parkinsonian medication could be reinstated if the patient experienced worsened EPS.	Primary:<br>- PANSS-total<br><br>Secondary:<br>- PANSS Marder factors<br>- CGI-S<br><br>Follow up (days): 3, 8, 15 and then weekly until day 57. Then bi-weekly until last visit.

	- DSM-IV: Schizophrenia<br>- Acute exacerbation for <6 months<br>- PANSS 70-120<br>- BMI ≥ 40kg/m2<br>- Disease duration: At least 1 year<br><br>Substance abuse allowed: No<br>Hospitalized at least 2 weeks<br><br>Must meet inclusion criteria after washout: Not relevant	- Olanzapine: 91.0 (11.15)<br>- PLB: 90.2 (10.39)<br><br>Completed the study: 60%<br><br>LOCF/ITT-population: Yes	- Oral benzodiazepines for agitation, anxiety or sleep difficulties but ≤ 6mg/d of lorazepam or equivalent during week 1-2 and an equivalent dose of 3 mg/d of lorazepam thereafter.	Interrater reliability: All investigators and staff involved in rating patients were trained on the key efficacy assessments. Raters were instructed and had to demonstrate acceptable rating accuracy as a condition for being qualified. No κ-value.
Bugarski-Kirola et al. 2014[14]	RCT, DB, PLB-controlled, parallel-group, phase II/III 4-arm study<br><br>Duration:<br>- Screening: up to 2 weeks<br>- DB treatment: 4 weeks<br>- Follow up: 4 weeks<br><br>Run-in period:<br>- Pre baseline washout: No<br>- Pre study trial-drug phase: No<br><br>Inclusion criteria:<br>- DSM-IV: schizophrenia<br>- Acute exacerbation<br>- PANSS 80-120 and score of ≥ 4 on at least two items: Delusions, conceptual disorganization, hallucinatory behavior, suspiciousness, unusual thought content<br>- CGI-S ≥ 4<br><br>Substance abuse allowed: NSPEC<br>Hospitalized for the 4 weeks DB treatment period<br><br>Must meet inclusion criteria after washout: Not relevant	Randomized, N= 301<br><br>Age, mean, years: 40 (range 18-65)<br><br>Sex, male: 74.3%<br><br>Enriched population: Yes<br><br>PLB-responders withdrawn: Not relevant<br><br>PANSS-total mean (SD) at BL:<br>- Bitopertin 10 mg: 64.8 (9.5)<br>- Bitopertin 30 mg: 65.9 (10.4)<br>- Olanzapine: 63.0 (9.5)<br>- PLB: 65.1 (8.7)<br><br>Completed the study: 72.4%<br><br>LOCF/ITT-population: Yes	Bitopertin at two dosages (n=153) vs Olanzapine 15 mg (n=61) vs PLB (n=79)<br><br>Administration: Once daily<br><br>Dose titration: No<br><br>Fixed doses: Yes<br><br>Allowed supplemental medication:<br>- Benztropine ≤ 4mg/d for emergent EPS.<br>- Lorazepam or equivalent ≤ 6 mg/d on days 1-7 and ≤ 4mg/d on days 8-28 for anxiety, agitation and restlessness.<br>- Zolpidem <10mg/d for insomnia.	Primary:<br>- PANSS total<br><br>Secondary:<br>- PANSS-positive<br>- PANSS-negative<br>- CGI-S<br>- NOSIE<br><br>Follow up: week 2, 3, 4, 5, 6, 8<br><br>Interrater reliability: Rated by both side raters and centralized raters. The discrepancy between the two ratings was not allowed to exceed an 8-point difference. No κ-value.

Shen et al. 2014[15]	RCT, DB, PLB-controlled, parallel-group trial<br>4-arm study<br><br>Duration: 6 weeks<br><br>Run-in period:<br>- Pre baseline washout: Yes (7 days)<br>- Pre study trial-drug phase: No<br><br>Inclusion criteria:<br>- DSM-IV: Schizophrenia<br>- Acute exacerbation<br>- PANSS 70-120<br>- PANSS-positive ≥ 20 and scores of ≥ 4 on at least two items: Delusions, conceptual disorganization, hallucinatory behavior, suspiciousness, unusual thought content<br>- CGI-S ≥ 4<br><br>Substance abuse allowed: No<br>Hospitalized at study entry and at least 4 weeks during DB-treatment<br><br>Must meet inclusion criteria after washout: Yes	Randomized, N= 289<br><br>Age, mean, years (SD): 40.2 (10.3)<br><br>Sex, male: 72.3%<br><br>Enriched population: Yes<br><br>PLB-responders withdrawn: NSPEC<br><br>PANSS-total mean (SD) at BL, central rated:<br>- Vabicaserin 200mg/d: 94.45 (12.07)<br>- Vabicaserin 400mg/d: 95.14 (10.30)<br>- Olanzapine: 94.52 (11.67)<br>- PLB: 94.72 (10.21)<br><br>Completed the study: 38%<br><br>LOCF/ITT-population: Yes	Vabicaserin at two dosages (n=147) vs Olanzapine 15mg/d (n=71) vs PLB (n=71)<br><br>Administration: B.i.d.<br><br>Dose titration: No<br><br>Fixed doses: Yes<br><br>Allowed supplemental medication:<br>Lorazepam, clorazepate dipotassium, benztropine, biperiden, trihexyphenidyl Hcl, zaleplon and zolpidem.	Primary:<br>- PANSS-total<br><br>Secondary:<br>- PANSS-positive<br>- PANSS-negative<br>- CGI-S<br>- BPRS<br>- CGI-I<br><br>Follow up days: 7, 14, 21, 28, 35, 42<br><br>Interrater reliability: Comparison of results between a side rater and central rater. No κ-value.
Davidson 2007[16]	RCT, DB, PLB-controlled<br>5-arm study<br><br>Duration: 6 weeks<br><br>Run-in period:<br>- Pre baseline drug washout: yes (3 days)<br>- Pre study trial drug phase: no<br><br>Inclusion criteria:<br>- DSM-IV: schizophrenia<br>- Acute exacerbation<br>- Disease duration: at least 1 year<br>- PANSS: 70-120	Randomized, N= 618<br><br>Age, mean, years (SD): 36.8 (10.6)<br><br>Sex, male: 68%<br><br>Enriched population: yes<br><br>PLB-responders withdrawn: NSPEC<br><br>PANSS-total mean (SD) at BL:<br>- Paliperidone ER 3 mg: 91.6 (12.2)	Paliperidone ER (n=359) at three different dosages vs. Olanzapine10 mg (n=126) vs. PLB (n=120)<br><br>Administration: once daily<br><br>Dose titration: patients in the group receiving PER 15mg were initiated on 12 mg/d on days 1-7 and increased to 15mg/d by day 8.<br><br>Fixed doses: yes<br><br>Allowed supplemental medication:	Primary<br>- PANSS-total<br><br>Secondary<br>- GCI-S<br>- PSP<br>- PANSS Marder factor<br><br>Follow up (days): 4, 8, 15, 22, 29, 36, 43<br><br>Interrater reliability: NSPEC. No κ-value.

	Substance abuse allowed: no<br>Hospitalized during first 2 weeks<br><br>Must meet inclusion criteria after washout: NSPEC	- Paliperidone ER 9 mg: 93.9 (13.2)<br>- Paliperidone ER 15 mg: 92.3 (12.3)<br>- Olanzapine: 93.3 (12.2)<br>- PLB: 93.9 (12.7)<br>- Total: 93.0 (12.5)<br><br>Completed the study: 59%<br><br>LOCF/ITT-population: yes	- Benzodiazepine and antidepressants continuation if the patient had been on a stable dose for ≥ 3 months before the study.<br>- Benzodiazepine: The dosage could be equivalent to lorazepam ≤ 6mg/d during screening and to day 7, ≤ 3mg/d day 8-14 and ≤ 2mg/d days 15-42<br>- Benztropine 1-2 mg twice daily or 2mg three times daily	
Kane 2007[17]	RCT, DB, PLB-controlled<br>5-arm study<br><br>Duration: 6 weeks<br><br>Run-in period:<br>- Pre baseline drug washout: yes (3 days)<br>- Pre study trial drug phase: no<br><br>Inclusion criteria:<br>- DSM-IV: schizophrenia<br>- Acute exacerbation<br>- Disease duration: schizophrenia for at least 1 year<br>- PANSS: 70-120<br><br>Substance abuse allowed: no<br>Hospitalized for first 2 weeks thereafter weekly return for assessment<br><br>Must meet inclusion criteria after washout: NSPEC	Randomized, N= 415<br><br>Age, mean, years (SD): 37.1 (10.9)<br><br>Sex, male: 52%<br><br>Enriched population: yes<br><br>PLB-responders withdrawn: NSPEC<br><br>PANSS-total mean (SD) at BL:<br>- Paliperidone ER 6 mg: 94.3 (10.5)<br>- Paliperidone ER 9 mg: 93.2 (11.9)<br>- Paliperidone ER 12 mg: 94.6 (11.0)<br>- Olanzapine: 93.0 (10.7)<br>- PLB: 94.1 (10.7)<br>- Total: 93.9 (11.0)<br><br>Completed the study: 66%<br><br>LOCF/ITT-population: yes	Paliperidone ER (n=374) at three different dosages vs. Olanzapine10mg/d (n=128) vs. PLB (n=126)<br><br>Administration: once daily<br><br>Dose titration: no<br><br>Fixed doses: yes<br><br>Allowed supplemental medication:<br>- Benzodiazepine and antidepressants continuation if the patient had been on a stable dose for ≥ 3 months before the study.<br>- Benzodiazepine: The dosage could be equivalent to lorazepam ≤ 6mg/d during screening and to day 7, ≤ 3mg/d days 8-14 and ≤ 2mg/d days 15-42. Patients not on a stable dose, discontinued benzodiazepines in week 2.<br>- Benztropine 1-2 mg twice daily or 2mg three times daily	Primary<br>- PANSS-total<br><br>Secondary<br>- CGI-S<br>- PSP<br><br>Follow up (days): 4, 8, 15, 22, 29, 36, 43<br><br>Interrater reliability: NSPEC. No κ-value.

Potkin et al. 2020[18]	RCT, DB, PLB-controlled<br>3-arm study<br><br>Duration: 4 weeks<br><br>Run-in period:<br>- Pre baseline drug washout: No<br>- Pre study trial drug phase: No<br><br>Inclusion criteria:<br>- DSM-IV: schizophrenia<br>- Acute exacerbation<br>- PANSS: ≥ 80 and scores of ≥ 4 on at least three items: Delusions, conceptual disorganization, hallucinatory behavior, suspiciousness<br>- CGI-S ≥ 4<br>- BMI 18-40kg/m^2<br><br>Substance abuse allowed: no<br>Hospitalized for at least the first 2 weeks<br><br>Must meet inclusion criteria after washout: NSPEC	Randomized, N= 401<br><br>Age, mean (SD): 41.1 (11.4)<br><br>Sex, male: 60.8%<br><br>Enriched population: yes<br><br>PLB-responders withdrawn: NSPEC<br><br>PANSS-total mean (SD) at BL:<br>- Olanzapine: 100.6 (12.1)<br>- PLB: 102.7 (11.9)<br><br>Completed the study: 87.8%<br><br>LOCF/ITT-population: yes	Olanzapine + Samidorphan (n=132) vs Olanzapine10mg/d (n=132) vs. PLB (n=133)<br><br>Administration: once daily<br><br>Dose titration: yes<br>At day 1-2 the dose of olanzapine was 10 mg. On day 3 the dose was increased to 20 mg. At the end of week 1 the dose could be decreased for tolerability to 10 mg.<br><br>Fixed doses: yes<br><br>Allowed supplemental medication:<br>- Beta-blockers<br>- Antihistamines<br>- Anticholinergics for the treatment of akathisia or extrapyramidal symptoms<br>- Lorazepam ≤ 2 mg/d for extrapyramidal symptoms	Primary<br>- PANSS-total<br><br>Secondary<br>- CGI-S<br>- PANSS-positive<br>- PANSS-negative<br><br>Follow up (days): 7, 14, 21, 28<br><br>Interrater reliability: NSPEC. No κ-value.
Egan et al. 2013 [19]	RCT, DB, PLB-controlled<br>3-arm study<br><br>Duration: 4 weeks<br><br>Run-in period:<br>- Pre baseline drug washout: yes (3-7 days)<br>- Pre study trial drug phase: No<br><br>Inclusion criteria:<br>- 18-55 years<br>- DSM-IV: schizophrenia<br>- Disease duration >1 year<br>- Acute exacerbation for 3 days to 6 weeks	Randomized, N= 216<br><br>Age, mean, years: 37<br><br>Sex, male: 58%<br><br>Enriched population: yes<br><br>PLB-responders withdrawn: yes<br><br>PANSS-total mean (SD) at BL:<br>- Olanzapine: 96.4 (11.2)<br>- PLB: 96.0 (12.9)<br><br>Completed the study: 73.1%	MK-8998 16 mg bid (n=86) vs Olanzapine 15mg/d (n=47) vs. PLB (n=83)<br><br>Administration: twice daily<br><br>Dose titration: yes<br>Olanzapine 10 mg/d for 1 week, then 15 mg/d for weeks 2-4.<br><br>Fixed doses: yes<br><br>Allowed supplemental medication:<br>- Benztropine, Biperiden and trihexyphenidyl allowed for the treatment of extrapyramidal symptoms<br>- Lorazepam ≤ 2 mg/d	Primary<br>- PANSS-total<br><br>Secondary<br>- CGI-S<br>- MADRS<br><br>Follow up (days): 4, 7, 14, 21, 28<br><br>Interrater reliability: Raters certified. PANSS ratings were reviewed by blinded, independent PANSS experts for internal consistency and

	- PANSS: ≥ 70 and scores of ≥ 4 on at least two items: Delusions, conceptual disorganization, hallucinatory behavior, suspiciousness, unusual thought content<br>- CGI-S ≥ 4<br><br>Substance abuse allowed: no<br>Hospitalization during the study<br><br>Must meet inclusion criteria after washout: NSPEC	LOCF/ITT-population:Yes	- Zolpidem ≤ 10 mg in any 24h period<br>- Temazepam ≤ 30 mg in any 24h period	relationship to prior ratings from the same patient.<br>No κ-value.
Kinon et al. 2011 [20]	RCT, DB, PLB-controlled<br>6-arm study<br><br>Duration: 4 weeks<br><br>Run-in period:<br>- Pre baseline drug washout: yes (3 days)<br>- Pre study trial drug phase: No<br><br>Inclusion criteria:<br>- 18-65 years<br>- DSM-IV: schizophrenia<br>- BPRS total score extracted from the PANSS of ≥ 45 and scores of ≥ 4 on at least two items: conceptual disorganization, hallucinatory behavior, suspiciousness, unusual thought content<br>- CGI-S ≥ 4<br><br>Substance abuse allowed: NSPEC<br>Hospitalization during the study<br><br>Must meet inclusion criteria after washout: No	Randomized, N= 669<br><br>Age, mean, years (SD): 38.8 (11.4)<br><br>Sex, male: 56.5%<br><br>Enriched population: No<br><br>PLB-responders withdrawn: No<br><br>PANSS-total mean (SD) at BL:<br>- Olanzapine: 99.6 (10.0)<br>- PLB: 97.6 (12.1)<br><br>Completed the study: 58.1%<br><br>LOCF/ITT-population: NSPEC	LY2140023 vs Olanzapine 15mg/d (n=62) vs. PLB (n=122)<br><br>Administration: twice daily<br>Dosage groups,<br>- LY2140023 5mg bid (n=121)<br>- LY2140023 20 mg bid (n=122)<br>- LY2140023 40 mg bid (n=120)<br>- LY2140023 80 mg bid (n=122)<br><br>Dose titration: NSPEC<br><br>Fixed doses: yes<br><br>Allowed supplemental medication:<br>- Benzodiazepines<br>- Anticholinergics<br>- Lorazepam	Primary<br>- PANSS-total<br><br>Secondary<br>- PANSS positive<br>- PANSS negative<br>- PANSS general psychopathology<br>- CGI-S<br><br>Follow up (days): 7, 14, 21, 28<br><br>Interrater reliability: NSPEC.<br>No κ-value.
Litman et al. 2014 [21]	RCT, DB, PLB-controlled<br>3-arm study<br><br>Duration: 4 weeks<br><br>Run-in period:	Randomized, N= 106<br><br>Age, mean, years: 39.2<br><br>Sex, male: 95.3%	AZD2624 40mg vs Olanzapine 15mg/d (n=22) vs. PLB (n=41)<br><br>Administration: once daily<br><br>Dose titration: NSPEC	Primary<br>- PANSS-total<br><br>Secondary<br>- CGI-S

	- Pre baseline drug washout: yes (3-7 days)<br>- Pre study trial drug phase: No<br><br>Inclusion criteria:<br>- 18-65 years<br>- DSM-IV: schizophrenia (paranoid, undifferentiated, disorganized, catatonic, residual)<br>- PANSS total ≥ 70<br><br>Substance abuse allowed: No<br>Hospitalization: NSPEC<br><br>Must meet inclusion criteria after washout: NSPEC	Enriched population: Yes<br><br>PLB-responders withdrawn: No<br><br>PANSS-total mean (SD) at BL:<br>- Olanzapine: 100.59<br>- PLB: 102.1<br><br>Completed the study: 76.4%<br><br>LOCF/ITT-population: Yes	Fixed doses: yes<br><br>Allowed supplemental medication:<br>- Lorazepam for agitation or anxiety up to 8 mg/d<br>- Zolpidem tartrate up to 10 mg/d for insomnia<br>- Benztropine mesylate up to 2 mg per 8-hour period for extrapyramidal symptoms<br>- Acetaminophen for pain up to 3g	Follow up (days): 1, 8, 15, 22, 28<br><br>Interrater reliability:<br>NSPEC.<br>No κ-value.
Mosolov et al. 2011 [22]	RCT, DB, PLB-controlled<br>3-arm study<br><br>Duration: 4 weeks<br><br>Run-in period:<br>- Pre baseline drug washout: yes<br>- Pre study trial drug phase: No<br><br>Inclusion criteria:<br>- 18-65 years<br>- DSM-IV: schizophrenia<br>- BPRS score ≥ 4 on at least two items: conceptual disorganization, hallucinatory behavior, suspiciousness, unusual thought content<br>- CGI-S ≥ 4<br><br>Substance abuse allowed: No<br>Hospitalization: NSPEC<br><br>Must meet inclusion criteria after washout: NSPEC	Randomized, N= 196<br><br>Age, mean, years:<br>- Olanzapine: 42.3 (±13.0)<br>- PLB: 41.0 (±14.1)<br><br>Sex, male: NSPEC<br><br>Enriched population: No<br><br>PLB-responders withdrawn: No<br><br>PANSS-total mean at BL: 94.8 (±11.4)<br><br>Completed the study: 60.2% (118)<br><br>LOCF/ITT-population: yes	LY2140023 40 mg bid (n=98) vs Olanzapine 15mg/d (n=34) vs. PLB (n=63)<br><br>Administration: twice daily<br><br>Dose titration: NSPEC<br><br>Fixed doses: yes<br><br>Allowed supplemental medication: NSPEC	Primary<br>- PANSS-total<br><br>Secondary<br>- CGI-S<br>- PANSS-positive<br>- PANSS-negative<br>- HAM-A<br><br>Follow up (days): 7, 14, 21, 28<br><br>Interrater reliability:<br>Training using videotaped interviews.<br>No κ-value.

Study ID	Design	Population	Intervention	Outcome measure
Shen et al. 2008 [23]	RCT, PLB-controlled<br>4-arm study<br><br>Duration: 6 weeks<br><br>Run-in period:<br>- Pre baseline drug washout: yes (7 days ± 3 days)<br>- Pre study trial drug phase: NSPEC<br><br>Inclusion criteria:<br>- 18-65 years<br>- Acute exacerbation of schizophrenia<br><br>Substance abuse allowed: NSPEC<br>Hospitalization: NSPEC<br><br>Must meet inclusion criteria after washout: NSPEC	Randomized, N= 313<br><br>Age, mean, years: NSPEC<br><br>Sex, male: NSPEC<br><br>Enriched population: No<br><br>PLB-responders withdrawn: NSPEC<br><br>PANSS-total mean (SD) at BL: NSPEC<br><br>Completed the study: NSPEC<br><br>LOCF/ITT-population: Yes	Experimental compound vs Olanzapine 15mg/d (n=68) vs. PLB (n=68)<br><br>Administration: NSPEC<br><br>Dose titration: NSPEC<br><br>Fixed doses: yes<br><br>Allowed supplemental medication: NSPEC	Primary<br>- PANSS-total<br><br>Follow up (days): 7, 14, 21, 28, 35, 42<br><br>Interrater reliability: κ-value 0.91.
**INTRAMUSCULAR OLANZAPINE**				
**Study ID**	**Design**	**Population**	**Intervention**	**Outcome measure**
Lauriello et al. 2008[24]	RCT, DB, PLB-controlled<br>4-arm study<br><br>Duration: 8 weeks<br><br>Run-in period:<br>- Pre baseline washout: Yes (2-7 days)<br>- Pre study trial-drug phase: No<br><br>Inclusion criteria:<br>- DSM-IV: Schizophrenia, residual type, disorganized or catatonic<br>- BPRS ≥ 30<br>- Disease duration: At least 1 year<br><br>Substance abuse allowed: No<br>Hospitalized for washout and at least 2 weeks of DB treatment.	Randomized, N= 404<br><br>Age, mean (SD), years:<br>- Olanzapine LAI 210 mg/2 weeks: 39.8 (10.8)<br>- Olanzapine LAI 300 mg/2 weeks: 41.5 (11.1)<br>- Olanzapine LAI 405 mg/4 weeks: 39.5 (11.4)<br>- PLB: 42.6 (11.2)<br><br>Sex, male: 70.5%<br><br>Enriched population: No<br><br>PLB-responders withdrawn: NSPEC	Olanzapine LAI at three dosages (n=304) vs PLB (n=98)<br><br>Administration: Once daily<br>Dosage groups,<br>- Olanzapine LAI 210 mg/2 weeks (n=106)<br>- Olanzapine LAI 300 mg/2 weeks (n=98)<br>- Olanzapine LAI 405 mg/4 weeks (n=100)<br><br>Fixed doses: Yes<br><br>Allowed supplemental medication:<br>- Benzodiazepines/sedative hypnotics as sleep aids if equivalent to ≤ 2 mg/d of Lorazepam<br>- Anticholinergics for emergent EPS if equivalent to ≤ 6 mg/d of Biperiden<br>- Prophylactic use of these was prohibited.	Primary:<br>- PANSS-total<br><br>Secondary:<br>- PANSS-positive<br>- PANSS-negative<br>- BPRS<br>- CGI-S<br>- CGI-I<br><br>Follow up (days): 3, 7, 14, 21, 28, 35, 42, 49, 56<br><br>Interrater reliability: NSPEC. No κ-value.

	Must meet inclusion criteria after washout: Yes	PANSS-total mean (SD) at BL:<br>- Olanzapine LAI 210 mg/2 weeks: 99.6 (15.8)<br>- Olanzapine LAI 300 mg/2 weeks: 102.6 (15.6)<br>- Olanzapine LAI 405 mg/4 weeks: 101.3 (14.4)<br>- PLB: 100.6 (16.7)<br><br>hCompleted the study: 66%<br>LOCF/ITT-population: Yes		
**ORAL QUETIAPINE**				
**Study ID**	**Design**	**Population**	**Intervention**	**Outcome measure**
Fabre et al. 1995[25]	RCT, DB, PLB-controlled<br><br>Duration: 21 days<br><br>Run-in period:<br>- Pre baseline washout: Yes (2 days)<br>- Pre study trial-drug phase: No<br><br>Inclusion criteria:<br>- Man<br>- DSM-III: Schizophrenia<br>- BPRS ≥ 30<br>- CGI-S ≥ 3<br><br>Substance abuse allowed: No<br>Hospitalized at inclusion<br><br>Must meet inclusion criteria after washout: NSPEC	Randomized, N= 12<br><br>Age, mean (SD), years:<br>- Quetiapine: 34 (20-46)<br>- PLB: 35 (24-45)<br><br>Sex, male: 100%<br><br>Enriched population: No<br><br>PLB-responders withdrawn: NSPEC<br><br>BPRS-total mean (SD) at BL:<br>- Quetiapine: 40.5 (8.5)<br>- PLB: 44.0 (12.4)<br><br>Completed the study: 91.7% (11)<br><br>LOCF/ITT-population: Yes	Quetiapine 250 mg (n=8) vs PLB (n=4)<br><br>Administration: Once daily<br><br>Dose titration: Quetiapine 25mg/d initially and increased by 25-50 mg approximately every 4 days until the target dose was reached.<br><br>Fixed doses: Yes<br><br>Allowed supplemental medication:<br>- Chloral hydrate ≤ 2500 mg/d for agitation (500mg) and insomnia (500-1000mg)<br>- Benztropine mesylate oral 1-4 mg/d and parenteral 1-2 mL for EPS<br>- Oral diphenhydramine hydrochloride (50mg) for akathisia<br>- Acetaminophen	Primary:<br>- BPRS, 18-items, score 1-7 at each item<br>- CGI<br><br>Follow up: BPRS was completed daily, CGI at baseline, day 7 and twice weekly thereafter<br><br>Interrater reliability: NSPEC. No κ-value.

Borison et al. 1996[26]	RCT, DB, PLB-controlled, parallel-group<br><br>Duration: 6 weeks<br><br>Run-in period:<br>- Pre baseline washout: Yes (2-10 days)<br>- Pre study trial-drug phase: No<br><br>Inclusion criteria:<br>- DSM-III: Schizophrenia, chronic or subchronic<br>- Acute exacerbation<br>- BPRS ≥ 45 and a score of ≥ 4 of ≥ 2 of the items: Conceptual disorganization, suspiciousness, hallucinatory behavior, unusual thought content<br>- CGI-S ≥ 4<br><br>Substance abuse allowed: No<br>Hospitalization: NSPEC<br><br>Must meet inclusion criteria after washout: Yes	Randomized, N= 109<br><br>Age, mean (range), years: 36 (18-58)<br><br>Sex, male: 90%<br><br>Enriched population: No<br><br>PLB-responders withdrawn: NSPEC<br><br>PANSS-total mean (SD) at BL:<br>- Quetiapine: 55.8 (8.3)<br>- PLB: 54.1 (7.0)<br><br>Completed the study: 45.8%<br><br>LOCF/ITT-population: Yes	Quetiapine (n=53) vs PLB (n=53)<br><br>Administration: t.i.d.<br><br>Mean dose: 307 mg/d (range 58-526 mg/d)<br><br>Dose titration: 25 mg/d on day 1-2. Thereafter the dose was increased by tablets of 25, 50, 100 and 200 mg until an adequate therapeutic effect.<br><br>Fixed doses: no, maximum daily dose was 750 mg, but doses greater than 500 mg were limited to 14 days.<br><br>Allowed supplemental medication:<br>- Acetaminophen<br>- Chloral hydrate for acute agitation (500mg) and insomnia (500-1000mg) up to 2000mg in 24 hours. Not permitted within 6 hours of efficacy assessments.<br>- Lorazepam 1-2 mg oral or I.M. for agitation or insomnia if chloral hydrate was ineffective. Up to 8 mg in 24 hours and only on day 1-7. Not permitted within 12 hours of efficacy assessments.<br>- EPS rated moderately and duration ≥24 hours were treated with benztropine mesylate ≤ 4mg/d oral or ≤ 2mg/d parenterally for ≤ 3 days per episode.<br>- Akathisia: 50 mg diphenhydramine hydrochloride	Primary:<br>- BPRS, 18-items<br>- CGI-S<br><br>Secondary:<br>- BPRS-factor score<br>- CGI-I<br>- SANS<br><br>Follow up (days): 0, 7, 14, 21, 28, 35, 42<br><br>Interrater reliability: NSPEC. No κ-value.
Arvanitis et al. 1997[27]	RCT, DB, PLB-controlled<br>7-arm study<br><br>Duration: 6 weeks<br><br>Run-in period:<br>- Pre baseline washout: Yes (7 days)	Randomized, N= 361<br><br>Age, mean (range), years: 37 (18-64)<br><br>Sex, male: 75.9%	Quetiapine fumarate at five dosages (n=255) vs Haloperidol 12 mg (n=50) vs PLB (n=51)<br><br>Administration: t.i.d.<br>Dosage groups,<br>- Quetiapine 75 mg (n=52)<br>- Quetiapine 150 mg (n=48)	Primary:<br>- BPRS, 18-items, score of 0-6 at each item<br>- CGI-S<br><br>Secondary:<br>- SANS

	- Pre study trial-drug phase: No<br><br>Inclusion criteria:<br>- DSM-III: Schizophrenia<br>- Acute exacerbation<br>- BPRS ≥ 27 and a score of 3 on ≥ 2 of the items: Conceptual disorganization, suspiciousness, hallucinatory behavior, unusual thought content<br>- CGI ≥4<br><br>Substance abuse allowed: NSPEC<br>Hospitalized at least at study entry<br><br>Must meet inclusion criteria after washout: Yes	Enriched population: No<br><br>PLB-responders withdrawn: Yes, if ≥ 20% decrease in BPRS-score or a greater than 1-point decrease in CGI-S during the placebo lead-in phase.<br><br>BPRS-total mean (SE) at BL:<br>- Quetiapine 75 mg: 45.7 (10.9)<br>- Quetiapine 150 mg: 47.2 (10.1)<br>- Quetiapine 300 mg: 45.3 (10.9)<br>- Quetiapine 600 mg: 43.5 (11.3)<br>- Quetiapine 750 mg: 45.7 (11.0)<br>- PLB: 45.3 (9.2)<br><br>Completed the study: 41.3% (149)<br>LOCF/ITT-population: Yes	- Quetiapine 300 mg (n=51)<br>- Quetiapine 600 mg (n=51)<br>- Quetiapine 750 mg (n=53)<br><br>Dose titration: The target dose was reached by day 7, if needed at least by day 14<br><br>Fixed doses: Yes<br><br>Allowed supplemental medication:<br>- Chloral hydrate for insomnia (500-1000) and acute agitation (500 mg), limited to 2000 mg/day. Not permitted within 6 hours of efficacy assessments.<br>- Lorazepam 1-2 mg orally or I.M., permitted through day 14 and ≤ 8mg/d on an emergency basis only. Not permitted within 12 hours of efficacy assessments.<br>- Benztropine mesylate for EPS	- CGI-I<br><br>Follow up (days): 7, 14, 21, 28, 35, 42<br><br>Interrater reliability: Investigators were trained and tested on BPRS and SANS scoring using videotapes. To pass, they were required to rate 80% of the items within one point of the reference-rater for each of the test interviews. No κ-value.
Small et al. 1997[28]	RCT, DB, PLB-controlled<br>3-arm study<br><br>Duration: 6 weeks<br><br>Run-in period:<br>- Pre baseline washout: Yes (1-2 days)<br>- Pre study trial-drug phase: No<br><br>Inclusion criteria:<br>- DSM-III: Schizophrenia – chronic or subchronic<br>- Acute exacerbation<br>- BPRS ≥ 27 and a score of ≥ 4 on ≥ 2 on the BPRS positive-symptom cluster<br>- CGI ≥ 4<br><br>Substance abuse allowed: No<br>Hospitalized at inclusion	Randomized, N= 286<br><br>Age, mean (SD), years:<br>- Quetiapine high dose: 36 (9)<br>- Quetiapine low dose: 37 (9)<br>- PLB: 38 (10)<br><br>Sex, male: 71.3%<br><br>Enriched population: No<br><br>PLB-responders withdrawn: NSPEC<br><br>BPRS mean (SD) at BL:<br>- Quetiapine high dose: 41.0 (9.6)<br>- Quetiapine low dose: 38.9 (9.8)<br>- PLB: 38.4 (9.7)	Quetiapine at two dosages (n=186) vs PLB (n=94)<br><br>Administration: Once daily<br>Dosage groups:<br>- Quetiapine high dose (max 750mg/d) (n=94)<br>- Quetiapine low dose (max 250mg/d) (n=92)<br>Daily dose of 750 mg was limited to 14 days, after that reduction to 500 mg or less.<br><br>Fixed doses: No.<br><br>Allowed supplemental medication:<br>- Chloral hydrate 500-1000mg orally, maximum of 2000mg/d, could not be administrated within 6 hours of assessments<br>- Lorazepam 1-2 mg orally or I.M., could not be administrated within 24 hours of assessments	Primary:<br>- BPRS<br>- CGI-S<br><br>Secondary:<br>- BPRS-positive cluster<br>- CGI-I<br>- SANS/PANSS-negative subscale<br><br>Follow up (days): 7, 14, 21, 28, 35, 42<br><br>Interrater reliability: Personal trained in the use of the assessments. No κ-value.

	Must meet inclusion criteria after washout: NSPEC	Completed the study: 44.4%<br><br>LOCF/ITT-population: Yes	- If EPS ≥ 24 hours and rated moderate: treatment with 50 mg diphenhydramine hydrochloride or ≤ 4mg benztropine mesylate for up to 3 days.	
Potkin et al. 2006[29]	RCT, DB, PLB-controlled<br>3-arm study<br><br>Duration: 6 weeks<br>- 2 weeks monotherapy with study drug<br>- 4 weeks with additional psychotropic medication if needed<br><br>Run-in period:<br>- Pre baseline drug washout: No<br>- Pre study trial-drug phase: No<br><br>Inclusion criteria:<br>- DSM-IV: Schizophrenia (paranoid, disorganized or undifferentiated type) or schizoaffective disorder<br>- Acute exacerbation ≤ 4 weeks<br>- Score of ≥ 4 on ≥ 2 on the PANSS items: Hostility, excitement, tension, uncooperativeness, poor impulse control and a total score of these items of ≥ 17.<br>- CGI ≥ 5<br><br>Substance abuse allowed: Yes<br>Hospitalized for first 3 weeks<br><br>Must meet inclusion criteria after washout: Not relevant	Randomized, N= 382<br><br>Age, mean (SD), years:<br>- Risperidone: 34.7 (9.6)<br>- Quetiapine: 34.2 (9.8)<br>- PLB: 36.1 (9.8)<br><br>Sex, male: 65.7%<br><br>Enriched population: No<br><br>PLB-responders withdrawn: Not relevant<br><br>PANSS-total mean at BL:<br>- Risperidone: 95.0 (18.0)<br>- Quetiapine: 97.3 (19.1)<br>- PLB: 94.3 (18.2)<br><br>Completed the study: 74.8%<br><br>LOCF / ITT population: Yes	Quetiapine 400-600mg (n=156) vs. Risperidone 4-6mg (n=152) vs. PLB (n=71)<br><br>Administration: Once daily<br>Dosage, mean (SD at day 14):<br>- Quetiapine: 523.8 (168.2)<br>- Risperidone: 4.32 (1.09)<br><br>Dose titration: Day 1-5 the quetiapine group was titrated from 50mg/d to the target dose of 400mg/d (≤ 70kg) or 600mg/d (>70kg). On day 8 investigators could increase quetiapine dose to 600mg/d (≤ 70kg) or 800mg/d (>70kg).<br><br>Fixed doses: no<br><br>Allowed supplemental medication:<br>- Day 1-14: zolpidem, zaleplon, zopiclone for insomnia and injectable lorazepam, sodium amytal, midazolam for agitation and restlessness<br>- After day 14: any psychotropic medication necessary except drugs known to interact with CYP2D6 and CYP3A4 and drugs with potential thyroid toxicity.<br>- Benztropine mesylate or equivalent for movement disorders permitted during the entire study if necessary.	Primary:<br>- PANSS-total<br><br>Secondary:<br>- PANSS Marder factors<br>- CGI-S<br>- HAM-D-17<br><br>Follow up (days): 3, 5, 7, 9, 14, 21, 28, 42<br><br>Interrater reliability: Raters trained to use the scales.<br>No κ-value.
Canuso et al. 2009[30]	RCT, DB, PLB-controlled<br>3-arm study<br><br>Duration: 6 weeks<br>- 2 weeks monotherapy with study drug	Randomized, N= 399<br><br>Age, mean (SD), years:<br>- Quetiapine: 36.9 (10.2)<br>- Paliperidone ER: 35.7 (11.6)<br>- PLB: 36.1 (10.4)	Quetiapine 600-800mg (n=157) vs Paliperidone ER 9-12 mg (n=157) vs PLB (n=80)<br><br>Administration: Once daily<br>Dosage, mean (SD) during monotherapy phase:<br>- Paliperidone ER: 10.4 (1.7)	Primary:<br>- PANSS-total<br><br>Secondary:<br>- CGI-S

	- 4 weeks with additional psychotropic medication if needed<br><br>Run-in period:<br>- Pre baseline drug washout: No<br>- Pre study trial-drug phase: No<br><br>Inclusion criteria:<br>- DSM-IV: Schizophrenia (paranoid, disorganized or undifferentiated type)<br>- Acute exacerbation, >4 days and <4 weeks<br>- PANSS score of ≥ 4 at ≥ 2 of the items: Hostility, excitement, tension, uncooperativeness, poor impulse control and a score of ≥ 17 on these items<br>- CGI-I ≥ 5<br>- Hospitalized or required hospitalization<br><br>Substance abuse allowed: Yes, but excluded if acute psychotic symptoms are explained by substance use<br>Hospitalized at inclusion and for at least 10 days<br><br>Must meet inclusion criteria after washout: Not relevant	Sex, male: 66.5%<br><br>Enriched population: Yes<br><br>PLB-responders withdrawn: Not relevant<br><br>PANSS-total mean at BL:<br>- Quetiapine: 101.3 (13.3)<br>- Paliperidone ER: 102.8 (13.1)<br>- PLB: 103.8 (15.7)<br><br>Completed the study monotherapy phase: 85.5%<br><br>LOCF / ITT population: Yes	- Quetiapine: 690.9 (134.3)<br><br>Dose titration: Quetiapine 50mg/d on day 1, 100mg/d on day 2, 200mg/d on day 3, 400mg/d on day 4, 600mg/d on day 5 and optional increase to 800mg/d on day 8. After day 14 the dose was fixed.<br><br>Fixed doses: no<br><br>Allowed supplemental medication:<br>- Day 1-14: Injectable lorazepam, amobarbital sodium or midazolam for agitation or restlessness, zaleplon, zopiclone or zolpidem for insomnia and benztropine mesylate or equivalent for movement disorders.<br>- After day 14: any psychotropic medication except risperidone, additional paliperidone ER, quetiapine, drugs that interact with CYP2A4, lithium, herbal or over-the-counter medication that have psychotropic effects and drugs that prolong the QTc-interval.	Follow up (days): 3, 5, 7, 9, 14, 21, 28, 42, endpoint<br><br>Interrater reliability: NSPEC. No κ-value.
**QUETIAPINE EXTENDED RELEASE**				
**Study ID**	**Design**	**Population**	**Intervention**	**Outcome measure**
Loebel et al 2013[31]	RCT, DB, PLB-controlled<br>4-arm study<br><br>Duration: 6 weeks<br><br>Run-in period:<br>- Pre baseline washout: Yes (3-7 days)<br>- Pre study trial-drug phase: No<br><br>Inclusion criteria:<br>- DSM-IV: Schizophrenia<br>- Acute exacerbation ≤ 2 months<br>- CGI-S ≥4	Randomized, N= 488<br><br>Age, mean (SD), years:<br>- Lurasidone 80 mg: 36.2 (10.9)<br>- Lurasidone 160 mg: 37.9 (11.3)<br>- Quetiapine XR: 37.4 (10.4)<br>- PLB: 37.4 (10.8)<br><br>Sex, male: 68.6%<br><br>Enriched population: No	Lurasidone at two dosages (n=246) vs quetiapine XR 600 mg (n=116) PLB (n=120)<br><br>Administration: Once daily (evening)<br>Dosage groups,<br>- Lurasidone 80 mg (n=125)<br>- Lurasidone 160 mg (n=121)<br><br>Dose titration: The Quetiapine XR 600mg/d group was treated with 300mg/d on day 1-2 before being increased to their target dose.<br><br>Fixed doses: Yes	Primary:<br>- PANSS-total<br><br>Secondary:<br>- PANSS subscales<br>- CGI-S<br>- NSA-16<br>- MADRS<br>- QWB-SA<br><br>Follow up (days): 4, 7, 14, 21, 28, 35, 42

	- PANSS ≥ 80 and score of ≥ 4 on ≥ 2 of items: Delusions, conceptual disorganization, hallucinations, unusual thought content and suspiciousness<br>- Disease duration > 1 year<br><br>Substance abuse allowed: NSPEC<br>Hospitalized at least 21 days<br><br>Must meet inclusion criteria after washout: Yes	PLB-responders withdrawn: NSPEC<br><br>PANSS-total mean (SD) at BL:<br>- Lurasidone 80 mg: 97.7 (9.7)<br>- Lurasidone 160 mg: 97,5 (11.8)<br>- Quetiapine: 97.7 (10.2)<br>- PLB: 96.6 (10.2)<br><br>Completed the study: 72.3% (353)<br><br>LOCF/ITT-population: Yes	Allowed supplemental medication: NSPEC	Interrater reliability: NSPEC. No κ-value.
Kahn et al. 2007[32]	RCT, DB, PLB-controlled<br>5-arm study<br><br>Duration: 6 weeks<br><br>Run-in period:<br>- Pre baseline washout: Yes (2 days)<br>- Pre study trial-drug phase: No<br><br>Inclusion criteria:<br>- DSM-IV: Schizophrenia: catatonic, disorganized, paranoid or undifferentiated type<br>- Worsening of the patient's condition in the previous 3 weeks in the opinion of the investigator.<br>- PANSS ≥ 70 and a score of ≥ 4 for at least one of the following items: Delusions, conceptual disorganization, hallucinatory behavior, suspiciousness<br>- CGI-S ≥ 4<br><br>Substance abuse allowed: No<br>Both in- and outpatients included<br><br>Must meet inclusion criteria after washout: NSPEC	Randomized, N= 588<br><br>Age, mean (SD), years:<br>- XR 400 mg/d: 34.1 (9.6)<br>- XR 600 mg/d: 34.2 (9.9)<br>- XR 800 mg/d: 34.4 (10.3)<br>- IR 400 mg/d: 34.4 (10.2)<br>- PLB: 34.1 (12.1)<br><br>Sex, male: 60.2%<br><br>Enriched population: Yes<br><br>PLB-responders withdrawn: NSPEC<br><br>PANSS-total mean (SD) at BL:<br>- XR 400 mg/d: 95.8 (13.9)<br>- XR 600 mg/d: 96.8 (14.1)<br>- XR 800 mg/d: 97.3 (14.7)<br>- IR 400 mg/d: 96.5 (16.0)<br>- PLB: 96.2 (13.3)<br><br>Completed the study: 76%<br><br>LOCF/ITT-population: Yes	Quetiapine XR at 3 dosages (n=339) vs Quetiapine IR 400 mg (n=119) vs PLB (n=115)<br><br>Administration: B.i.d.<br>Dosage groups,<br>- Quetiapine XR 400 mg/d (n=111)<br>- Quetiapine XR 600 mg/d (n=111)<br>- Quetiapine XR 800 mg/d (n=117)<br><br>Fixed doses: Yes<br><br>Allowed supplemental medication:<br>- Anticholinergics to treat emergent EPS<br>- Lorazepam ≤ 6mg/d, only the first 6 days<br>- Oxazepam ≤ 60mg/d, only the first 6 days<br>- Hypnotics and sedatives continuation, taken at bedtime only<br>- Zolpidem, chloral hydrate, zaleplon and zopiclone	Primary:<br>- PANSS-total<br><br>Secondary:<br>- CGI-S<br>- CGI-I<br>- PANSS response rate<br>- PANSS positive subscale<br>- PANSS negative subscale<br>- PANSS general psychopathology<br>- PANSS aggression and hostility cluster<br>- PANSS depression cluster<br><br>Follow up (days): 7, 14, 21, 28, 42<br><br>Inter-rater reliability:<br>- Overall k= 0.819<br>- Lowest 0.379<br>- All other >0.49<br>- Majority >0.83

Lindenmayer et al. 2008[33]	RCT, DB, PLB-controlled<br>6-arm study<br><br>Duration: 6 weeks<br><br>Run-in period:<br>- Pre baseline washout: Yes (2 days)<br>- Pre study trial-drug phase: No<br><br>Inclusion criteria:<br>- DSM-IV: Schizophrenia: Catatonic, disorganized, paranoid or undifferentiated type<br>- Worsening of the patient's condition in the previous 3 weeks in the opinion of the investigator.<br>- PANSS ≥ 60 and a score of ≥ 4 for at least one of the following items: Delusions, conceptual disorganization, hallucinatory behavior, suspiciousness<br>- CGI-S ≥ 4<br><br>Substance abuse allowed: No<br>Hospitalized at least 10 days<br><br>Must meet inclusion criteria after washout: NSPEC	Randomized, N= 532<br><br>Age, mean (SD), years:<br>- XR 300 mg/d: 39.1 (11.2)<br>- XR 600 mg/d: 38.9 (9.3)<br>- XR 800 mg/d: 37.8 (10.5)<br>- IR 300 mg/d: 39.8 (10.6)<br>- IR 600 mg/d: 40.6 (9.7)<br>- PLB: 38.4 (10.1)<br><br>Sex, male: 74.7%<br><br>Enriched population: Yes<br><br>PLB-responders withdrawn: NSPEC<br><br>PANSS-total mean (SD) at BL:<br>- XR 300 mg/d: 91.8 (19.4)<br>- XR 600 mg/d: 92.5 (17.3)<br>- XR 800 mg/d: 89.2 (15.1)<br>- IR 300 mg/d: 89.5 (15.7)<br>- IR 600 mg/d: 88.6 (17.4)<br>- PLB: 91.3 (16.4)<br><br>Completed the study: 41.7%<br><br>LOCF/ITT-population: Yes	Quetiapine XR at three dosages (n=255) vs Quetiapine IR at two dosages (n=165) vs PLB (n=78)<br><br>Administration: B.i.d.<br>Dosage groups,<br>- Quetiapine XR 300 mg/d (n=83)<br>- Quetiapine XR 600 mg/d (n=87)<br>- Quetiapine XR 800 mg/d (n=85)<br>- Quetiapine IR 300 mg/d (n=85)<br>- Quetiapine IR 600 mg/d (n=80)<br><br>Fixed doses: Yes<br><br>Allowed supplemental medication:<br>- Anticholinergics to treat emergent EPS<br>- Lorazepam ≤ 4mg/d day 1-3 and ≤ 2 mg/d day 4-6<br>- Continuation medication for sleep if taken at bedtime only	Primary:<br>- PANSS-total<br>Secondary:<br>- CGI-S<br>- CGI-I<br>- PANSS positive subscale<br>- PANSS negative subscale<br>- PANSS general psychopathology<br><br>Follow up (days): 4, 8, 15, 28, 42<br><br>Inter-rater reliability:<br>At each center, a single certified PANSS rater conducted all PANSS assessments across visits. No κ-value.
		**ORAL ZOTEPINE**		
**Study ID**	**Design**	**Population**	**Intervention**	**Outcome measure**
Cooper et al. 2000 (March)[34]	RCT, DB, PLB-controlled, parallel-group<br>3-arm study<br><br>Duration: 8 weeks<br><br>Run-in period:	Randomized, N= 159<br><br>Age, mean (range), years:<br>- Zotepine: 39.6 (20.8-64.2)<br>- Chlorpromazine: 41.0 (20.4-60.6)<br>- PLB: 36.3 (19.4-62.7)	Zotepine 300mg/d (n=53) vs Chlorpromazine 600mg/d (n=52) vs PLB (n=53)<br><br>Administration: T.i.d.<br><br>Dose titration: Zotepine 150mg/d on days 1-6, 300mg/d on day 7 and onward. Chlorpromazine	Primary:<br>- BPRS, 18-items, score 0-7 at each item<br><br>Secondary:<br>- CGI-S<br>- CGI-I

	- Pre baseline washout: No (stop oral antipsychotics at study entry. If treated with depot antipsychotics ≥ 1 dose interval had to have been omitted before study entry)<br>- Pre study trial-drug phase: no<br><br>Inclusion criteria:<br>- DSM-III: Schizophrenia<br>- Acute episode or acute exacerbation of subchronic or chronic schizophrenia<br>- CGI-S ≥ 4<br><br>Substance abuse allowed: No<br>Hospitalization: NSPEC<br><br>Must meet inclusion criteria after washout: Not relevant	Sex, male: 72.3%<br><br>Enriched population: No<br><br>PLB-responders withdrawn: Not relevant<br><br>BPRS-total mean at BL:<br>- Zotepine: 60.2<br>- Chlorpromazine: 56.9<br>- PLB: 57.9<br><br>Completed the study: 56%<br><br>LOCF/ITT-population: Yes	200mg/d on days 1-6 and increased to 600mg/d on day 7 and onward.<br><br>Fixed doses: If the dose level was not tolerated the daily dose could be reduced to 150mg/d of zotepine and 300mg/d of chlorpromazine<br><br>Allowed supplemental medication:<br>- Psychotropic agents only benzodiazepines and chloral hydrate<br>- All other treatments allowed including anticholinergics for EPS.	- SANS<br><br>Follow up (week): 1, 2, 4, 6, 8<br><br>Interrater reliability: NSPEC. No κ-value.
Cooper et al. 2000 (June)[35]	RCT, DB, PLB-controlled, parallel-group trial<br><br>Duration: 26 weeks<br><br>Run-in period:<br>- Pre baseline washout: No<br>- Pre study trial-drug phase: No<br><br>Inclusion criteria:<br>- DSM-III: Schizophrenia, chronic<br>- History of recurrence within the past 18 months<br>- CGI-S ≥ 3<br><br>Substance abuse allowed: No<br>Hospitalization: both in- and outpatients included<br><br>Must meet inclusion criteria after washout: Not relevant	Randomized, N= 121<br><br>Age, mean (SD), years:<br>- Zotepine: 43.0 (12.5)<br>- PLB: 41.6 (12.4)<br><br>Sex, male: 68.9%<br><br>Enriched population: No<br><br>PLB-responders withdrawn: Not relevant<br><br>BPRS-total mean (SD) at BL:<br>- Zotepine: 49.8 (13.1)<br>- PLB: 48.4 (11.6)<br><br>Completed the study: Not relevant<br><br>LOCF/ITT-population: Yes	Zotepine 300 mg/d (n=61) vs PLB (n=58)<br><br>Administration: T.i.d.<br><br>Dose titration: Zotepine 150mg/d initially and increased to 300mg/d over the first 4 days.<br><br>Fixed doses: If the dose level was not tolerated the daily dose could be reduced to 150mg/d of zotepine or placebo equivalent.<br><br>Allowed supplemental medication:<br>- Benzodiazepine as hypnotics	Primary:<br>- Time to recurrence<br><br>Secondary:<br>- BPRS<br>- CGI-S<br>- CGI-I<br>- SANS<br><br>Follow up (week): 2, 4, 8, 16, 20, 26<br><br>Interrater reliability: Training sessions chaired by a psychiatrist experienced in the use of the scales. No κ-value.

Moller et al. 2004[36]	RCT, DB, PLB-controlled, parallel group trial<br><br>Duration: 8 weeks<br><br>Run-in period:<br>- Pre baseline washout: Yes (for oral neuroleptic 5 halftimes and depot medication two injection intervals)<br>- Pre study trial-drug phase: No<br><br>Inclusion criteria:<br>- ICD-10: Schizophrenia, residual type<br>- Stable symptomatic condition the last 6 months prior to enrollment<br>- Not received neuroleptic treatment for symptom suppression the last 6 months, but only administrated for relapse prevention<br>- A score of ≥ 3 on 3 items of PANSS-negative subscale<br>- A score of ≤ 3 on ≤2 items of the PANSS-positive subscale<br>- A score of >3 on the item "blunted affect" and a score of >3 on item "Lack of spontaneity and flow of conversation" of the PANSS-negative subscale<br>- MADRS <20<br>- EPS <1.0<br><br>Substance abuse allowed: No<br>Hospitalization: NSPEC<br><br>Must meet inclusion criteria after washout: NSPEC	Randomized, N= 85<br><br>Age, mean (SD), years:<br>- Zotepine: 39.8 (11.9)<br>- PLB: 42.2 (9.9)<br><br>Sex, male: 53.2%<br><br>Enriched population: No<br><br>PLB-responders withdrawn: Yes, if ≥ 25% decrease in PANSS-total from screening to BL.<br><br>PANSS-total mean (SD) at BL:<br>- Zotepine: 81.2 (13.9)<br>- PLB: 77.6 (10.9)<br><br>Completed the study: 74%<br><br>LOCF/ITT-population: Yes	Zotepine (n=38) vs PLB (n=41)<br><br>Administration: Once daily<br>Dosage, mean (SD): 131 (49) mg/day (range 25-225 mg)<br><br>Dose titration: Initial zotepine dose was 25mg or 50mg/d and the maximum dose was 225 mg/d. The dose was increased by ≤ 100mg/week if necessary, according to the investigator.<br><br>Fixed doses: No<br><br>Allowed supplemental medication:<br>- Chloral hydrate ≤ 2 g or promethiazine 50 mg for sleep difficulties<br>- Biperiden for Parkinson disease<br>- Mild analgesics	Primary:<br>- PANSS-negative subscale<br><br>Secondary:<br>- PANSS-total<br>- PANSS-positive subscale<br>- PANSS general psychopathology<br>- MADRS<br>- CGI<br><br>Follow up (week): 1, 2, 3, 4, 6, 8<br><br>Interrater reliability: NSPEC. No κ-value.

B.i.d.= bis in die (twice a day), BL = Baseline (day 0), BMI = Body Mass Index, BPRS = Brief Psychiatric Rating Scale, CDSS= Calgary Depression Scale for Schizophrenia, CGI = Clinical Global Impression, DB = Double blinded, DSM = Diagnostic and Statistical Manual of Mental Disorders, EPS=Extrapyramidal Symptoms, ER = Extended release, HAM-A = Hamilton Anxiety Scale, HAM-D-17= Hamilton Rating Scale for Depression, ICD-10 = International Classification of Diseases, IR = immediate release, ITT = Intention To Treat, LAI =Long-Acting Injection , LOCF = Last Observation Carried Forward, MADRS= Montgomery–Åsberg Depression Rating Scale, N= randomized patients, n=ITT-population, NOSIE = Nurses Observation Scale for Inpatient Evaluation, NSA-16= Negative Symptom Assessment, NSPEC = non specified, PDS = Psychotic Depression Scale, PGI= Patient´s Global Impression Scores, PLB = Placebo, PANSS = Positive And Negative Syndrome Scale, PSP = Personal and Social Performance scale, QWB-SA= Quality of Well-being Scale, RCT = Randomized Controlled Trial, SANS =Scale for the Assessment of Negative Symptom, SD=standard deviation, SE = Standard error, T.i.d. = Ter in die (three times a day), XR = extended release.

Supplemental 4 – Risk of Bias Assessment

Illustrates the Risk of bias assessment using "A Revised Cochrane risk of bias tool for randomized trials, ROB2" [37]

D1	Randomisation process
D2	Deviations from the intended interventions
D3	Missing outcome data
D4	Measurement of the outcome
D5	Selection of the reported result

- \+ Low risk
- ! Some concerns
- \- High risk

Unique ID	Experimental	Outcome	D1	D2	D3	D4	D5	Overall
Potkin 2007	Asenapine, SL	PANSS	+	+	-	!	!	-
Kane 2010	Asenapine, SL	PANSS	+	+	-	!	!	-
Kinoshita 2016	Asenapine, SL	PANSS	+	+	-	+	!	-
Landbloom 2017	Asenapine, SL + Olanzapine	PANSS	+	+	-	!	!	-
Citrome 2020	Asenapine, TDD	PANSS	+	+	-	!	+	-
Lecrubier 2006	Olanzapine	PANSS	+	+	-	+	!	-
Meltzer 2011	Olanzapine	PANSS	+	+	-	!	!	-
Beasley 1996_1	Olanzapine	BPRS	!	+	-	!	!	-
Beasley 1996_2	Olanzapine	PANSS	!	+	-	!	!	-
Corrigan 2004	Olanzapine	PANSS	+	+	-	!	!	-
Marder 2007	Olanzapine	PANSS	+	+	-	!	!	-
Schmidt 2012	Olanzapine	PANSS	+	+	-	!	!	-
Bugarski-Kirola 2014	Olanzapine	PANSS	+	+	-	!	!	-
Shen 2014	Olanzapine	PANSS	+	+	-	+	!	-
Davidson 2007	Olanzapine	PANSS	+	+	-	!	!	-
Kane 2007	Olanzapine	PANSS	+	+	-	!	!	-
Potkin 2020	Olanzapine	PANSS	+	+	-	!	!	-

Egan 2013	Olanzapine	PANSS	+	+	-	+	!	-
Kinon 2011	Olanzapine	PANSS	+	+	-	!	!	-
Litman 2014	Olanzapine	PANSS	+	+	-	!	!	-
Mosolov 2011	Olanzapine	PANSS	+	+	-	!	!	-
Shen 2008	Olanzapine	PANSS	!	!	-	+	!	-
Lauriello 2008	Olanzapine, LAI	PANSS	+	+	-	!	!	-
Fabre 1995	Quetiapine	BPRS	!	+	-	!	!	!
Borison 1996	Quetiapine	BPRS	+	+	-	!	!	-
Arvanitis 1997	Quetiapine	BPRS	+	+	-	+	!	-
Small 1997	Quetiapine	BPRS	+	+	-	!	!	-
Potkin 2006	Quetiapine	PANSS	+	+	-	!	!	-
Canuso 2009	Quetiapine	PANSS	+	+	-	!	!	-
Loebel 2013	Quetiapine, XR	PANSS	+	+	-	!	!	-
Kahn 2007	Quetiapine + Quetapine, XR	PANSS	+	+	-	+	!	-
Lindenmayer 2008	Quetiapine + Quetiapine, XR	PANSS	+	+	-	+	!	-
Cooper 2000_1	Zotepine	BPRS	+	+	-	!	!	-
Cooper 2000_2	Zotepine	BPRS	+	+	-	!	!	-
Moller 2004	Zotepine	PANSS	+	+	-	!	!	-

Supplemental 5 – Forest plots

Pooled meta-analyses for immediate release and transdermal drug delivery antipsychotics

Shows the forest plots of the standardized mean difference in the changes of symptom score from baseline to week 1, 2, 3, 4, 5, 6 (green squares) with a 95% CI (bars) between either sublingual asenapine, transdermal asenapine, olanzapine, quetiapine or zotepine and placebo. The black diamonds are the summary effect estimates, which forms the dots in figure 3A.

Week 1:

	Antipsychotics			Placebo				Std. Mean Difference
Study or Subgroup	Mean	SD	Total	Mean	SD	Total	Weight	IV, Fixed, 95% CI
Beasley 1996_2 (OLZ)	-3.46	12.5	49	-2.56	12.3	49	3.3%	-0.07 [-0.47, 0.32]
Borrison 1996 (QTP)	-3.16	10.083	53	-0.7	10.1922	53	3.5%	-0.24 [-0.62, 0.14]
Canuso 2009 (QTP)	-12.88	15.9962	150	-11.6	15.9962	79	7.0%	-0.08 [-0.35, 0.19]
Citrome 2020 (ASN_T)	-5.7	10.1159	203	-4.77	8.4062	203	13.7%	-0.10 [-0.29, 0.09]
Cooper 2000_1 (ZOT)	-4.6	7.4	53	-1.4	7.9	53	3.5%	-0.42 [-0.80, -0.03]
Corrigan 2004 (OLZ)	-8.56	14.6215	93	-4.23	14.6215	85	5.9%	-0.29 [-0.59, 0.00]
Egan 2013 (OLZ)	-7.15	10.3977	45	-4.68	10.4215	78	3.8%	-0.24 [-0.60, 0.13]
Fabre 1995 (QTP)	-9.01	8.5	8	-5.59	13.2	4	0.4%	-0.31 [-1.52, 0.90]
Kane 2010 (ASN_S)	-7.99	10.0932	105	-6.19	9.8856	122	7.6%	-0.18 [-0.44, 0.08]
Meltzer 2011 (OLZ)	-10.45	20.9	121	-6.82	22.4219	114	7.9%	-0.17 [-0.42, 0.09]
Mosolov 2011 (OLZ)	-3.81	14.1109	34	1.91	20.24	63	2.9%	-0.31 [-0.73, 0.11]
Potkin 2006 (QTP)	-13.88	17.028	146	-16.38	17.028	68	6.2%	0.15 [-0.14, 0.43]
Potkin 2007 (ASN_S)	-6.41	7.5015	58	-3.99	6.9326	60	3.9%	-0.33 [-0.70, 0.03]
Potkin 2020 (OLZ)	-7.95	9.5934	132	-7.19	8.7647	133	8.9%	-0.08 [-0.32, 0.16]
Schmidt 2012 (OLZ)	-7.5	11.1866	93	-3.84	10.8454	99	6.4%	-0.33 [-0.62, -0.05]
Shen 2008 (OLZ)	-10.2	13.7712	68	-2.55	14.0186	68	4.4%	-0.55 [-0.89, -0.20]
Shen 2014 (OLZ)	-9.7	13.2712	71	-1.62	13.2712	71	4.6%	-0.61 [-0.94, -0.27]
Small 1997 (QTP)	-2.41	9.9753	92	-0.39	10.0233	92	6.2%	-0.20 [-0.49, 0.09]
**Total (95% CI)**			**1574**			**1494**	**100.0%**	**-0.20 [-0.28, -0.13]**

Heterogeneity: Chi² = 21.53, df = 17 (P = 0.20); I² = 21%
Test for overall effect: Z = 5.57 (P < 0.00001)

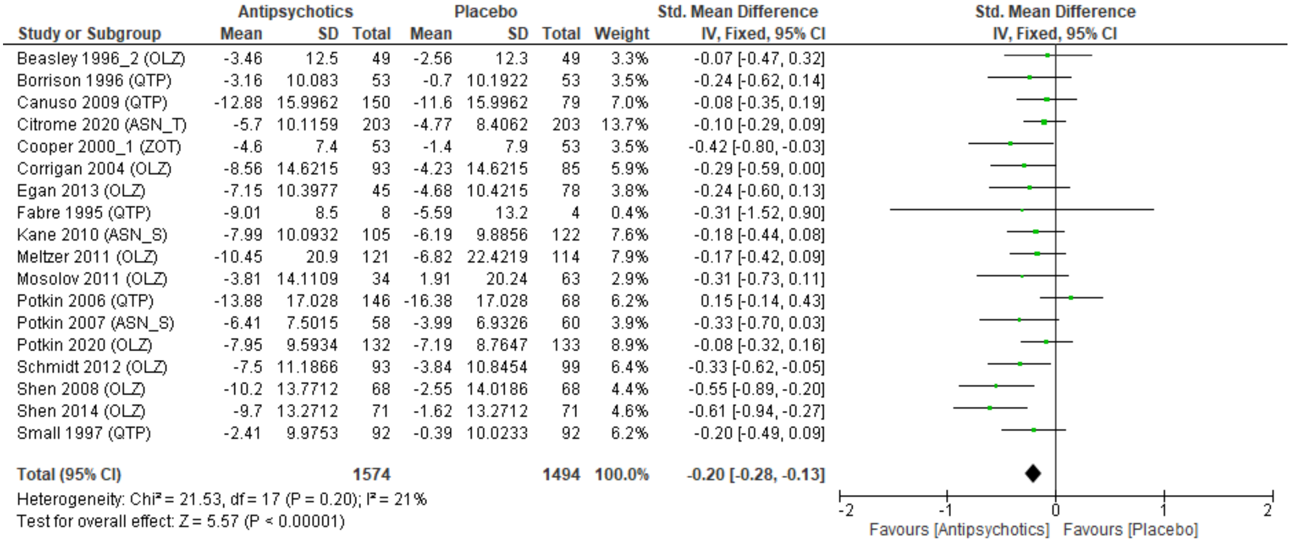

Week 2:

	Antipsychotics			Placebo				Std. Mean Difference
Study or Subgroup	Mean	SD	Total	Mean	SD	Total	Weight	IV, Fixed, 95% CI
Beasley 1996_2 (OLZ)	-8.1	12.5	47	-2.75	12.3	45	3.0%	-0.43 [-0.84, -0.01]
Borrison 1996 (QTP)	-5.6	14.997	53	-0.5	13.8322	53	3.5%	-0.35 [-0.73, 0.03]
Canuso 2009 (QTP)	-20.26	14.9351	134	-21.12	14.9351	68	6.0%	0.06 [-0.23, 0.35]
Citrome 2020 (ASN_T)	-10.31	10.1159	203	-8.27	10.5434	203	13.5%	-0.20 [-0.39, -0.00]
Cooper 2000_1 (ZOT)	-9	10.8	53	-2.4	11.9	53	3.4%	-0.58 [-0.97, -0.19]
Cooper 2000_2 (ZOT)	-3.38	15	61	-0.98	17.1	58	4.0%	-0.15 [-0.51, 0.21]
Corrigan 2004 (OLZ)	-18.14	19.0761	93	-8.56	19.0761	85	5.7%	-0.50 [-0.80, -0.20]
Egan 2013 (OLZ)	-11.42	15.0935	45	-8.01	15.2789	78	3.8%	-0.22 [-0.59, 0.15]
Fabre 1995 (QTP)	-17.05	8.5	8	-1.22	13.2	4	0.3%	-1.44 [-2.83, -0.05]
Kane 2010 (ASN_S)	-12	13.1673	105	-9.41	12.9231	122	7.5%	-0.20 [-0.46, 0.06]
Meltzer 2011 (OLZ)	-15.77	20.9	121	-10.23	22.4219	114	7.8%	-0.26 [-0.51, 0.00]
Mosolov 2011 (OLZ)	-10.72	18.2509	34	4.44	22.4624	63	2.8%	-0.71 [-1.14, -0.28]
Potkin 2006 (QTP)	-23.8	15.6222	129	-21.38	15.6222	56	5.2%	-0.15 [-0.47, 0.16]
Potkin 2007 (ASN_S)	-11.32	15.6288	58	-5.62	15.6288	60	3.9%	-0.36 [-0.73, 0.00]
Potkin 2020 (OLZ)	-13.56	12.2934	132	-11.41	12.3398	133	8.8%	-0.17 [-0.42, 0.07]
Schmidt 2012 (OLZ)	-13.41	13.6458	93	-4.74	13.7806	99	6.1%	-0.63 [-0.92, -0.34]
Shen 2008 (OLZ)	-10.14	15.4204	68	-2.78	15.6678	68	4.4%	-0.47 [-0.81, -0.13]
Shen 2014 (OLZ)	-9.95	14.3245	71	-1.47	15.1671	71	4.5%	-0.57 [-0.91, -0.24]
Small 1997 (QTP)	-4.57	12.2773	92	0.07	12.8528	92	6.0%	-0.37 [-0.66, -0.08]
**Total (95% CI)**			**1600**			**1525**	**100.0%**	**-0.31 [-0.38, -0.24]**

Heterogeneity: Chi² = 29.12, df = 18 (P = 0.05); I² = 38%
Test for overall effect: Z = 8.58 (P < 0.00001)

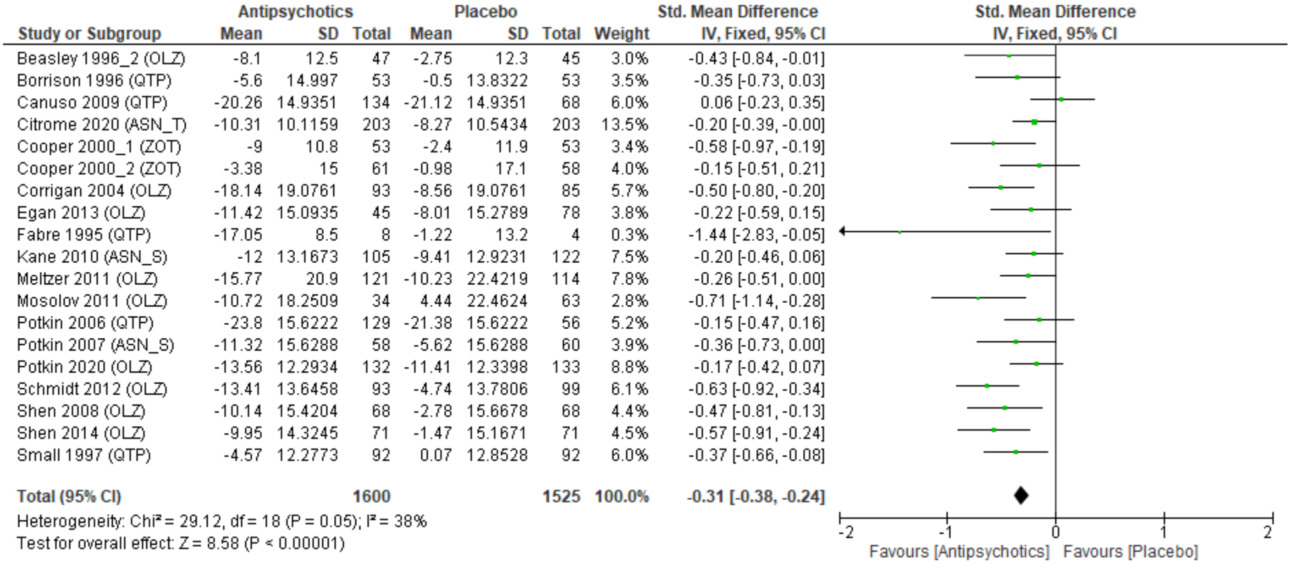

Week 3:

Study or Subgroup	Antipsychotics Mean	SD	Total	Placebo Mean	SD	Total	Weight	Std. Mean Difference IV, Fixed, 95% CI
Beasley 1996_2 (OLZ)	-7.03	12.5	43	-2.31	12.3	44	3.5%	-0.38 [-0.80, 0.05]
Borrison 1996 (QTP)	-7	15.2882	53	-1.97	15.179	53	4.3%	-0.33 [-0.71, 0.06]
Citrome 2020 (ASN_T)	-13.51	12.2531	203	-10.89	12.2531	203	16.7%	-0.21 [-0.41, -0.02]
Corrigan 2004 (OLZ)	-24.18	19.076	93	-13.8	19.076	85	7.1%	-0.54 [-0.84, -0.24]
Egan 2013 (OLZ)	-14.12	15.1605	45	-10.81	17.8402	78	4.7%	-0.19 [-0.56, 0.17]
Fabre 1995 (QTP)	-20.9	8.5	8	-4.7	13.2	4	0.3%	-1.47 [-2.87, -0.08]
Kane 2010 (ASN_S)	-13.87	14.1408	105	-10.93	14.6903	122	9.3%	-0.20 [-0.46, 0.06]
Meltzer 2011 (OLZ)	-20.88	20.9	121	-11.32	22.4219	114	9.5%	-0.44 [-0.70, -0.18]
Mosolov 2011 (OLZ)	-15.11	21.6911	34	7.12	25.9548	63	3.3%	-0.90 [-1.33, -0.46]
Potkin 2007 (ASN_S)	-17.01	17.0491	58	-6.41	17.0491	60	4.6%	-0.62 [-0.99, -0.25]
Potkin 2020 (OLZ)	-18.53	13.0976	132	-13.34	14.4734	133	10.7%	-0.37 [-0.62, -0.13]
Schmidt 2012 (OLZ)	-18.43	15.6709	93	-6.81	17.0143	99	7.4%	-0.71 [-1.00, -0.41]
Shen 2008 (OLZ)	-12.24	18.2241	68	-3.06	18.2241	68	5.4%	-0.50 [-0.84, -0.16]
Shen 2014 (OLZ)	-11.9	17.1472	71	-1.7	17.737	71	5.6%	-0.58 [-0.92, -0.25]
Small 1997 (QTP)	-6.43	14.6752	92	-0.71	14.1957	92	7.4%	-0.39 [-0.69, -0.10]
**Total (95% CI)**			**1219**			**1289**	**100.0%**	**-0.42 [-0.50, -0.34]**

Heterogeneity: Chi² = 22.17, df = 14 (P = 0.08); I² = 37%
Test for overall effect: Z = 10.25 (P < 0.00001)

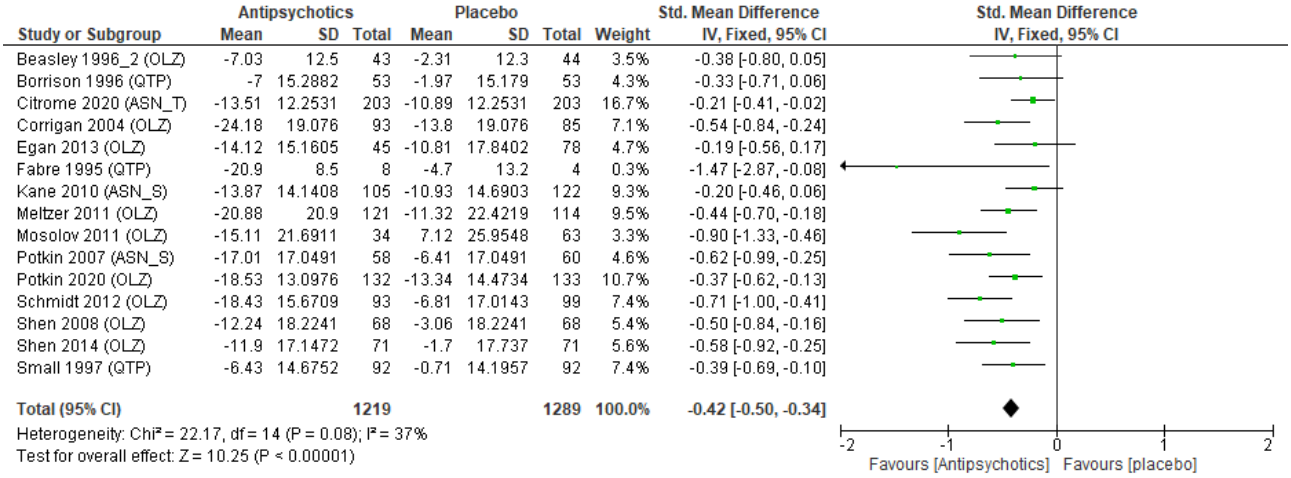

Week 4:

Study or Subgroup	Experimental Mean	SD	Total	Control Mean	SD	Total	Weight	Std. Mean Difference IV, Random, 95% CI
Beasley 1996_2 (OLZ)	-7.44	12.5	42	-2.09	12.3	42	3.9%	-0.43 [-0.86, 0.01]
Borrison 1996 (QTP)	-8.4	15.2882	53	-1.8	15.2882	53	4.5%	-0.43 [-0.81, -0.04]
Bugarski-Kirola 2014 (OLZ)	-14.9	16.6749	61	-11.9	16.8876	79	5.2%	-0.18 [-0.51, 0.16]
Citrome 2020 (ASN_T)	-15.95	13.108	203	-13.28	13.5354	203	7.8%	-0.20 [-0.40, -0.00]
Cooper 2000_1 (ZOT)	-11.8	11.9	53	-4.5	14.5	53	4.4%	-0.55 [-0.93, -0.16]
Cooper 2000_2 (ZOT)	-4.58	15	61	-3.2	17.1	58	4.8%	-0.09 [-0.45, 0.27]
Corrigan 2004 (OLZ)	-30.23	19.076	93	-17.13	19.076	85	5.7%	-0.68 [-0.99, -0.38]
Egan 2013 (OLZ)	-17	19.5209	45	-12.7	19.8715	78	4.7%	-0.22 [-0.58, 0.15]
Kane 2010 (ASN_S)	-14.52	15.1143	105	-12	15.2426	122	6.5%	-0.17 [-0.43, 0.10]
Kinnon 2011 (OLZ)	-20.68	24.2519	62	-14.6	24.2998	122	5.7%	-0.25 [-0.56, 0.06]
Litman 2014 (OLZ)	-8.24	14.84	22	4.82	14.99	41	2.9%	-0.86 [-1.40, -0.32]
Meltzer 2011 (OLZ)	-24.59	20.9	121	-12.91	22.4219	114	6.5%	-0.54 [-0.80, -0.28]
Mosolov 2011 (OLZ)	-19.13	22.3325	34	8.16	28.0185	63	3.8%	-1.03 [-1.48, -0.59]
Potkin 2007 (ASN_S)	-16.87	19.4513	58	-6.62	19.4513	60	4.7%	-0.52 [-0.89, -0.16]
Potkin 2020 (OLZ)	-22.8	14.9359	132	-17.5	14.9923	133	6.8%	-0.35 [-0.60, -0.11]
Schmidt 2012 (OLZ)	-20.74	17.3586	93	-7.41	18.8053	99	5.9%	-0.73 [-1.03, -0.44]
Shen 2008 (OLZ)	-14.16	18.9663	68	-3.74	19.2137	68	5.1%	-0.54 [-0.89, -0.20]
Shen 2014 (OLZ)	-14.2	17.9898	71	-2.23	18.369	71	5.2%	-0.65 [-0.99, -0.32]
Small 1997 (QTP)	-7.71	13.9079	92	-0.51	14.6752	92	5.9%	-0.50 [-0.80, -0.21]
**Total (95% CI)**			**1469**			**1636**	**100.0%**	**-0.45 [-0.55, -0.34]**

Heterogeneity: Tau² = 0.03; Chi² = 37.95, df = 18 (P = 0.004); I² = 53%
Test for overall effect: Z = 8.11 (P < 0.00001)

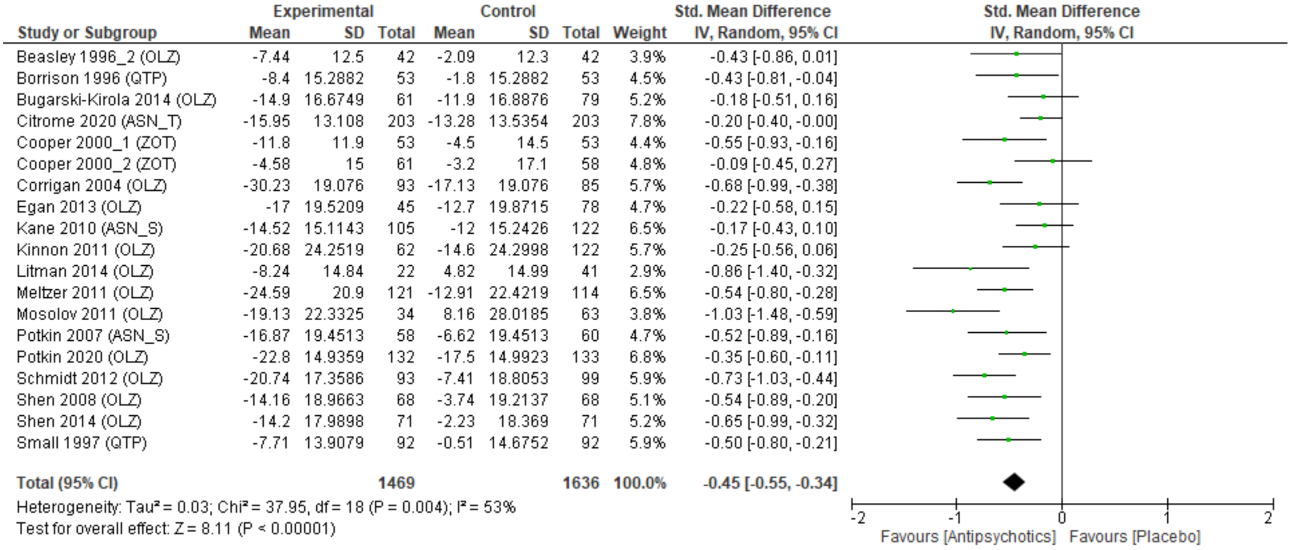

Week 5:

Study or Subgroup	Antipsychotics Mean	SD	Total	Placebo Mean	SD	Total	Weight	Std. Mean Difference IV, Random, 95% CI
Beasley 1996_2 (OLZ)	-12.53	12.5	22	-8.31	12.3	14	3.5%	-0.33 [-1.01, 0.34]
Borrison 1996 (QTP)	-8.7	16.6715	53	-1.3	16.7443	53	7.6%	-0.44 [-0.83, -0.05]
Citrome 2020 (ASN_T)	-18.29	14.8177	203	-14.45	15.2452	203	12.9%	-0.25 [-0.45, -0.06]
Corrigan 2004 (OLZ)	-35.57	19.076	93	-19.75	19.076	85	9.5%	-0.83 [-1.13, -0.52]
Kane 2010 (ASN_S)	-17.41	16.0365	105	-13.32	16.5128	122	10.8%	-0.25 [-0.51, 0.01]
Meltzer 2011 (OLZ)	-27.19	20.9	121	-14.91	22.4219	114	10.8%	-0.57 [-0.83, -0.30]
Potkin 2007 (ASN_S)	-15.94	17.9659	58	-4.77	17.9659	60	7.9%	-0.62 [-0.99, -0.25]
Schmidt 2012 (OLZ)	-21.87	17.3104	93	-5.94	19.651	99	9.8%	-0.86 [-1.15, -0.56]
Shen 2008 (OLZ)	-16.94	20.1208	68	-3.8	20.1208	68	8.5%	-0.65 [-0.99, -0.30]
Shen 2014 (OLZ)	-16.76	19.1695	71	-2.61	19.2959	71	8.6%	-0.73 [-1.07, -0.39]
Small 1997 (QTP)	-8.02	15.1548	92	-0.32	14.6752	92	9.9%	-0.51 [-0.81, -0.22]
**Total (95% CI)**			**979**			**981**	**100.0%**	**-0.55 [-0.69, -0.41]**

Heterogeneity: Tau² = 0.03; Chi² = 22.77, df = 10 (P = 0.01); I² = 56%
Test for overall effect: Z = 7.55 (P < 0.00001)

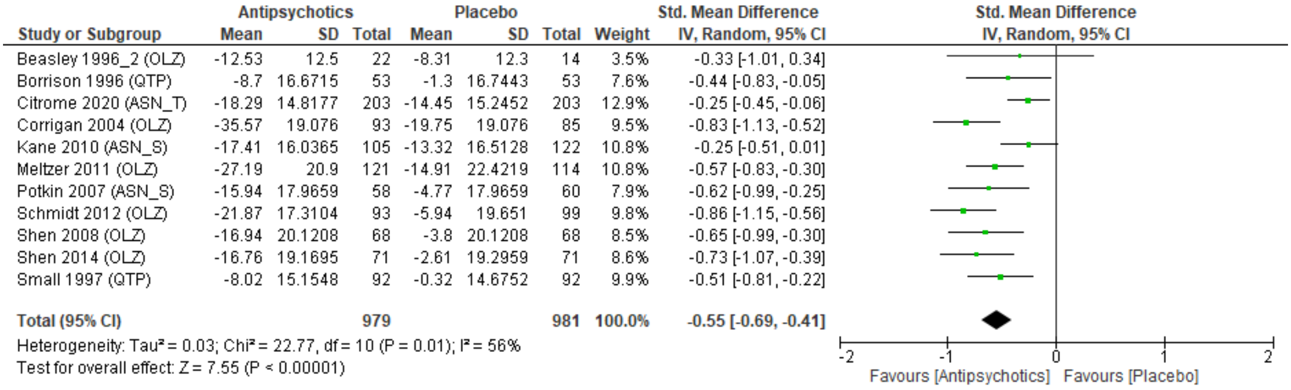

Week 6:

	Antipsychotics			Placebo				Std. Mean Difference
Study or Subgroup	Mean	SD	Total	Mean	SD	Total	Weight	IV, Random, 95% CI
Arvanitis 1997 (QTP)	-6.33	14.7058	53	1.71	14.7113	51	3.5%	-0.54 [-0.93, -0.15]
Beasley 1996_1 (OLZ)	-15.2	16.1	65	-3.1	17.5	62	3.9%	-0.72 [-1.08, -0.36]
Beasley 1996_2 (OLZ)	-15.6	12.5	19	-10.47	12.3	9	1.2%	-0.40 [-1.20, 0.40]
Borrison 1996 (QTP)	-8.1	17.3995	53	-2.1	17.3995	53	3.6%	-0.34 [-0.73, 0.04]
Citrome 2020 (ASN_T)	-20.39	16.1	203	-15.55	15.6726	203	6.5%	-0.30 [-0.50, -0.11]
Cooper 2000_1 (ZOT)	-14.8	13.7	53	-3.7	14.9	53	3.5%	-0.77 [-1.17, -0.37]
Corrigan 2004 (OLZ)	-31.5	19.076	93	-12.6	19.076	85	4.5%	-0.99 [-1.30, -0.67]
Davidson 2007 (OLZ)	-18.1	20.3	126	-2.8	20.9	120	5.4%	-0.74 [-1.00, -0.48]
Kahn 2007 (QTP, QTPXR)	-26.6	20.518	119	-18.8	20.518	115	5.4%	-0.38 [-0.64, -0.12]
Kane 2007 (OLZ)	-19.9	19	128	-4.1	23.2	126	5.4%	-0.74 [-1.00, -0.49]
Kane 2010 (ASN_S)	-19.44	17.1636	105	-14.56	16.8994	122	5.3%	-0.29 [-0.55, -0.02]
Kinoshita 2016 (ASN_S)	-14.17	20.0125	178	-0.95	20.1821	174	6.2%	-0.66 [-0.87, -0.44]
Landbloom 2017 (ASN_S)	-21.7	16.857	111	-16.2	17.0143	99	5.1%	-0.32 [-0.60, -0.05]
Landbloom 2017 (OLZ)	-21.6	15.563	45	-16.2	17.0143	99	3.9%	-0.32 [-0.68, 0.03]
Lindenmayer 2008 (QTP,QTPXR)	-6.97	17.4	80	-5.19	16.4	78	4.5%	-0.10 [-0.42, 0.21]
Marder 2007 (OLZ)	-18.4	19.9	105	-8	21.5	105	5.1%	-0.50 [-0.78, -0.23]
Meltzer 2011 (OLZ)	-28.7	20.9	121	-16	22.4219	114	5.3%	-0.58 [-0.85, -0.32]
Potkin 2007 (ASN_S)	-15.9	20.1155	58	-5.3	20.1155	60	3.8%	-0.52 [-0.89, -0.16]
Schmidt 2012 (OLZ)	-22.9	18.1301	93	-6.4	20.2977	99	4.8%	-0.85 [-1.15, -0.56]
Shen 2008 (OLZ)	-15.2	20.7805	68	-4.43	20.698	68	4.1%	-0.52 [-0.86, -0.17]
Shen 2014 (OLZ)	-14.7	20.2228	71	-2.7	20.5598	71	4.2%	-0.59 [-0.92, -0.25]
Small 1997 (QTP)	-8.7	15.7303	92	-1	15.4426	92	4.8%	-0.49 [-0.79, -0.20]
**Total (95% CI)**			**2039**			**2058**	**100.0%**	**-0.53 [-0.62, -0.44]**

Heterogeneity: Tau² = 0.02; Chi² = 43.62, df = 21 (P = 0.003); I² = 52%
Test for overall effect: Z = 11.20 (P < 0.00001)

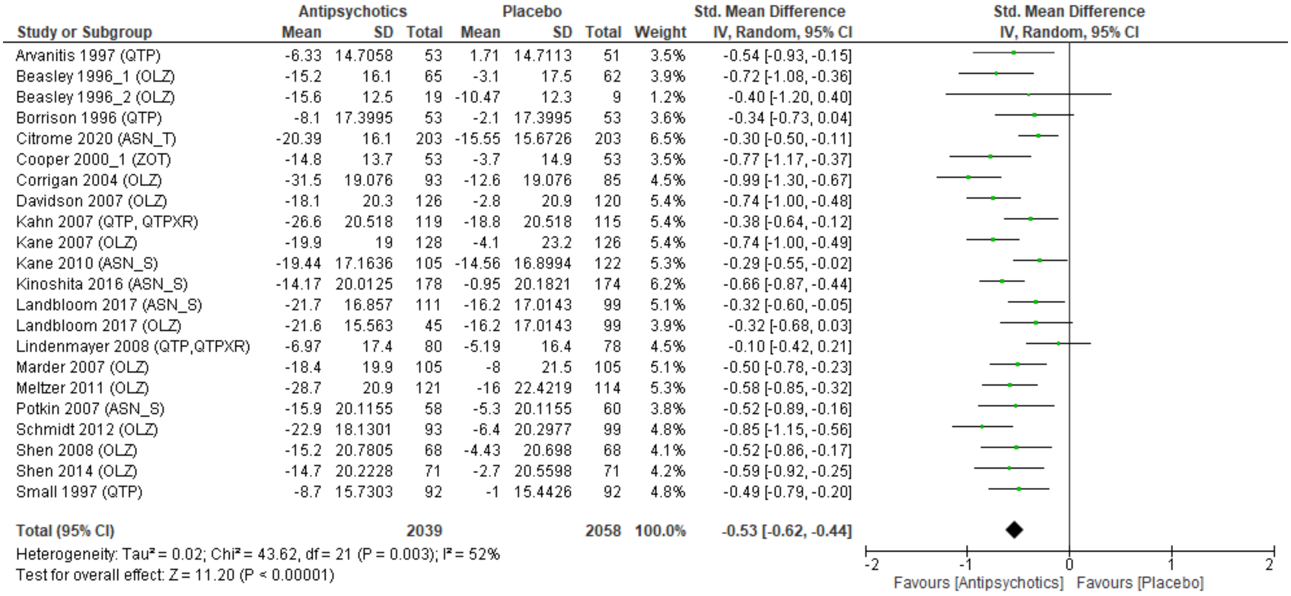

Asenapine

Shows the forest plots of the standardized mean difference in the changes of symptom score from baseline to week 1, 2, 3, 4, 5, 6 (green squares) with a 95% CI (bars) between sublingual asenapine and placebo. The black diamonds are the summary effect estimates, which forms the dots in figure 3B.

Week 1:

Study or Subgroup	Asenapine Mean	Asenapine SD	Asenapine Total	Placebo Mean	Placebo SD	Placebo Total	Weight	Std. Mean Difference IV, Fixed, 95% CI
Kane 2010 (ASN_S)	-7.99	10.0932	105	-6.19	9.8856	122	65.9%	-0.18 [-0.44, 0.08]
Potkin 2007 (ASN_S)	-6.41	7.5015	58	-3.99	6.9326	60	34.1%	-0.33 [-0.70, 0.03]
**Total (95% CI)**			**163**			**182**	**100.0%**	**-0.23 [-0.44, -0.02]**

Heterogeneity: Chi² = 0.45, df = 1 (P = 0.50); I² = 0%
Test for overall effect: Z = 2.14 (P = 0.03)

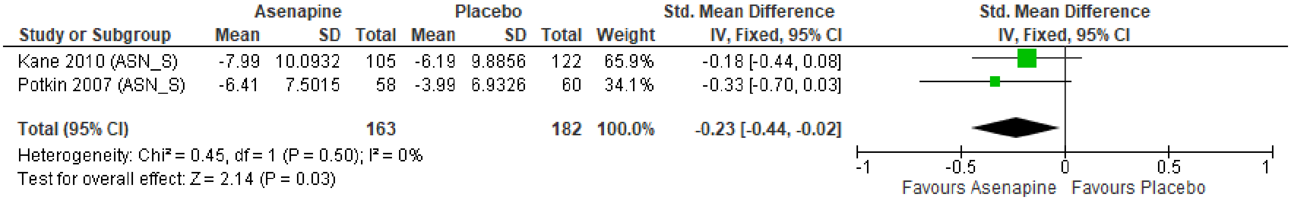

Week 2:

Study or Subgroup	Asenapine Mean	Asenapine SD	Asenapine Total	Placebo Mean	Placebo SD	Placebo Total	Weight	Std. Mean Difference IV, Fixed, 95% CI
Kane 2010 (ASN_S)	-12	13.1673	105	-9.41	12.9231	122	65.9%	-0.20 [-0.46, 0.06]
Potkin 2007 (ASN_S)	-11.32	15.6288	58	-5.62	15.6288	60	34.1%	-0.36 [-0.73, 0.00]
**Total (95% CI)**			**163**			**182**	**100.0%**	**-0.25 [-0.47, -0.04]**

Heterogeneity: Chi² = 0.52, df = 1 (P = 0.47); I² = 0%
Test for overall effect: Z = 2.34 (P = 0.02)

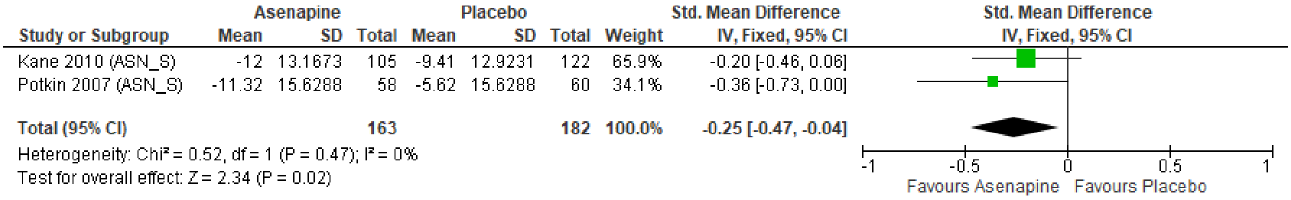

Week 3:

Study or Subgroup	Asenapine Mean	Asenapine SD	Asenapine Total	Placebo Mean	Placebo SD	Placebo Total	Weight	Std. Mean Difference IV, Fixed, 95% CI
Kane 2010 (ASN_S)	-13.87	14.1408	105	-10.93	14.6903	122	66.6%	-0.20 [-0.46, 0.06]
Potkin 2007 (ASN_S)	-17.01	17.0491	58	-6.41	17.0491	60	33.4%	-0.62 [-0.99, -0.25]
**Total (95% CI)**			**163**			**182**	**100.0%**	**-0.34 [-0.55, -0.13]**

Heterogeneity: Chi² = 3.22, df = 1 (P = 0.07); I² = 69%
Test for overall effect: Z = 3.13 (P = 0.002)

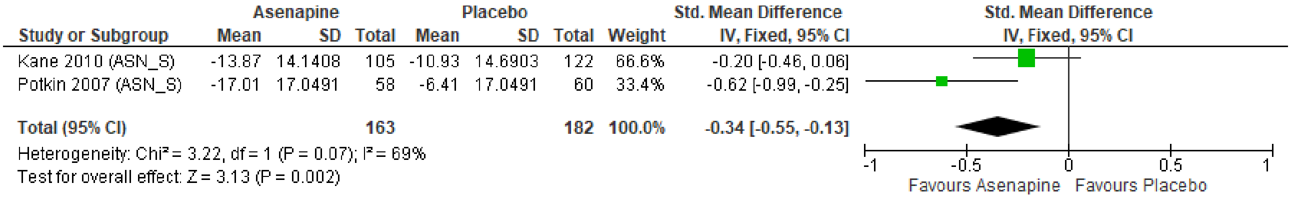

Week 4:

Study or Subgroup	Asenapine Mean	Asenapine SD	Asenapine Total	Placebo Mean	Placebo SD	Placebo Total	Weight	Std. Mean Difference IV, Fixed, 95% CI
Kane 2010 (ASN_S)	-14.52	15.1143	105	-12	15.2426	122	66.4%	-0.17 [-0.43, 0.10]
Potkin 2007 (ASN_S)	-16.87	19.4513	58	-6.62	19.4513	60	33.6%	-0.52 [-0.89, -0.16]
**Total (95% CI)**			**163**			**182**	**100.0%**	**-0.29 [-0.50, -0.07]**

Heterogeneity: Chi² = 2.42, df = 1 (P = 0.12); I² = 59%
Test for overall effect: Z = 2.63 (P = 0.009)

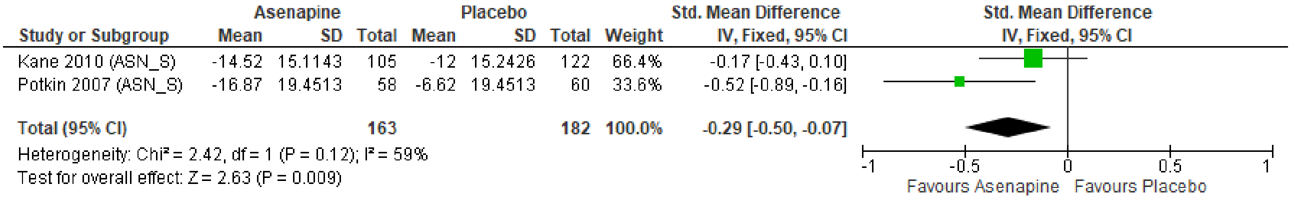

Week 5:

Study or Subgroup	Asenapine Mean	Asenapine SD	Asenapine Total	Placebo Mean	Placebo SD	Placebo Total	Weight	Std. Mean Difference IV, Fixed, 95% CI
Kane 2010 (ASN_S)	-17.41	16.0365	105	-13.32	16.5128	122	66.6%	-0.25 [-0.51, 0.01]
Potkin 2007 (ASN_S)	-15.94	17.9659	58	-4.77	17.9659	60	33.4%	-0.62 [-0.99, -0.25]
**Total (95% CI)**			**163**			**182**	**100.0%**	**-0.37 [-0.59, -0.16]**

Heterogeneity: Chi² = 2.53, df = 1 (P = 0.11); I² = 60%
Test for overall effect: Z = 3.42 (P = 0.0006)

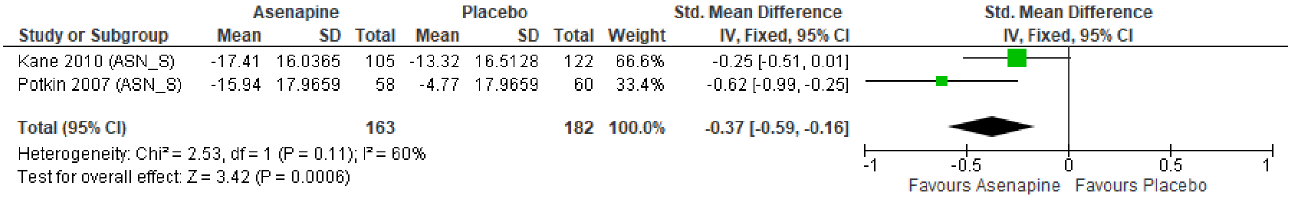

Week 6:

Study or Subgroup	Asenapine Mean	Asenapine SD	Asenapine Total	Placebo Mean	Placebo SD	Placebo Total	Weight	Std. Mean Difference IV, Fixed, 95% CI
Kane 2010 (ASN_S)	-19.44	17.1636	105	-14.56	16.8994	122	25.5%	-0.29 [-0.55, -0.02]
Kinoshita 2016 (ASN_S)	-14.17	20.0125	178	-0.95	20.1821	174	38.0%	-0.66 [-0.87, -0.44]
Landbloom 2017 (ASN_S)	-21.7	16.857	111	-16.2	17.0143	99	23.5%	-0.32 [-0.60, -0.05]
Potkin 2007 (ASN_S)	-15.9	20.1155	58	-5.3	20.1155	60	13.0%	-0.52 [-0.89, -0.16]
**Total (95% CI)**			**452**			**455**	**100.0%**	**-0.47 [-0.60, -0.33]**

Heterogeneity: Chi² = 5.98, df = 3 (P = 0.11); I² = 50%
Test for overall effect: Z = 6.91 (P < 0.00001)

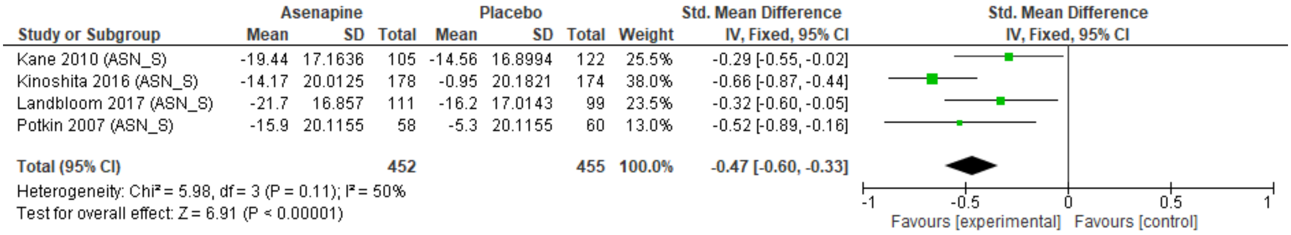

Asenapine, transdermal

Shows the forest plots of the standardized mean difference in the changes of symptom score from baseline to week 1, 2, 3, 4, 5, 6 (green squares) with a 95% CI (bars) between transdermal asenapine and placebo. The black diamonds are the summary effect estimates, which forms the dots in figure 3C.

Week 1:

Study or Subgroup	Asenapine Mean	Asenapine SD	Asenapine Total	Control Mean	Control SD	Control Total	Weight	Std. Mean Difference IV, Fixed, 95% CI
Citrome 2020 (ASN_T)	-5.7	10.1159	203	-4.77	8.4062	203	100.0%	-0.10 [-0.29, 0.09]
**Total (95% CI)**			**203**			**203**	**100.0%**	**-0.10 [-0.29, 0.09]**

Heterogeneity: Not applicable
Test for overall effect: Z = 1.00 (P = 0.31)

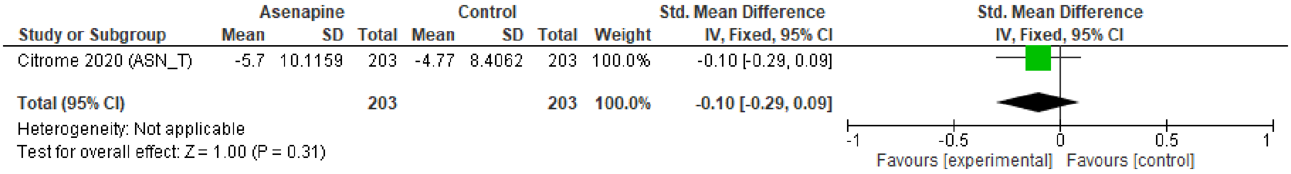

Week 2:

Study or Subgroup	Asenapine Mean	Asenapine SD	Asenapine Total	Control Mean	Control SD	Control Total	Weight	Std. Mean Difference IV, Fixed, 95% CI
Citrome 2020 (ASN_T)	-10.31	10.1159	203	-8.27	10.5434	203	100.0%	-0.20 [-0.39, -0.00]
**Total (95% CI)**			**203**			**203**	**100.0%**	**-0.20 [-0.39, -0.00]**

Heterogeneity: Not applicable
Test for overall effect: Z = 1.98 (P = 0.05)

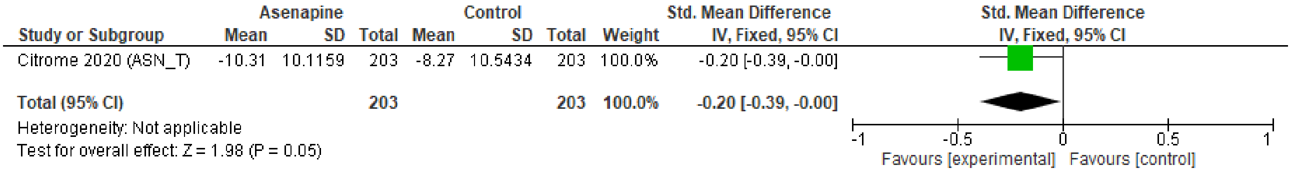

Week 3:

Study or Subgroup	Asenapine Mean	Asenapine SD	Asenapine Total	Control Mean	Control SD	Control Total	Weight	Std. Mean Difference IV, Fixed, 95% CI
Citrome 2020 (ASN_T)	-13.51	12.2531	203	-10.89	12.2531	203	100.0%	-0.21 [-0.41, -0.02]
**Total (95% CI)**			**203**			**203**	**100.0%**	**-0.21 [-0.41, -0.02]**

Heterogeneity: Not applicable
Test for overall effect: Z = 2.14 (P = 0.03)

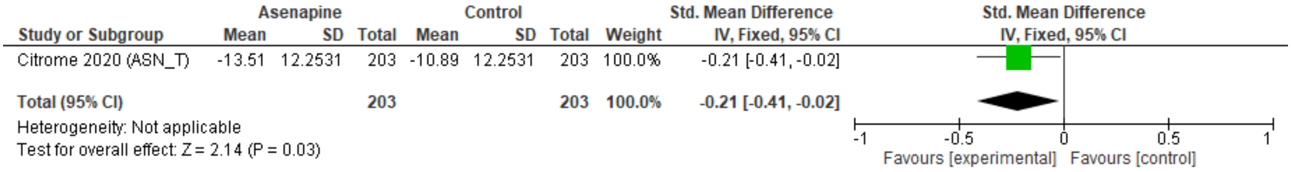

Week 4:

Study or Subgroup	Asenapine Mean	Asenapine SD	Asenapine Total	Control Mean	Control SD	Control Total	Weight	Std. Mean Difference IV, Fixed, 95% CI
Citrome 2020 (ASN_T)	-15.95	13.108	203	-13.28	13.5354	203	100.0%	-0.20 [-0.40, -0.00]
**Total (95% CI)**			**203**			**203**	**100.0%**	**-0.20 [-0.40, -0.00]**

Heterogeneity: Not applicable
Test for overall effect: Z = 2.01 (P = 0.04)

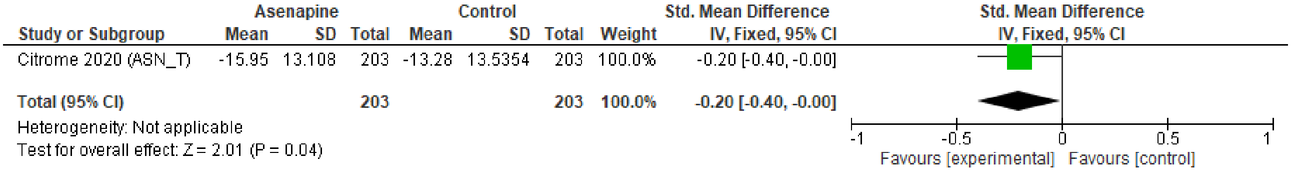

Week 5:

Study or Subgroup	Asenapine Mean	Asenapine SD	Asenapine Total	Control Mean	Control SD	Control Total	Weight	Std. Mean Difference IV, Fixed, 95% CI
Citrome 2020 (ASN_T)	-18.29	14.8177	203	-14.45	15.2452	203	100.0%	-0.25 [-0.45, -0.06]
**Total (95% CI)**			**203**			**203**	**100.0%**	**-0.25 [-0.45, -0.06]**

Heterogeneity: Not applicable
Test for overall effect: Z = 2.56 (P = 0.01)

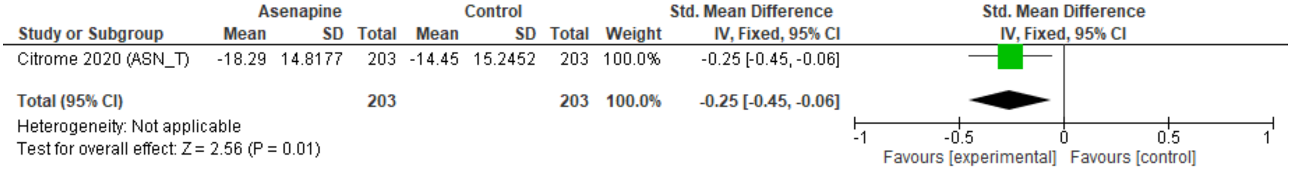

Week 6:

Study or Subgroup	Asenapine Mean	Asenapine SD	Asenapine Total	Control Mean	Control SD	Control Total	Weight	Std. Mean Difference IV, Fixed, 95% CI
Citrome 2020 (ASN_T)	-20.39	16.1	203	-15.55	15.6726	203	100.0%	-0.30 [-0.50, -0.11]
**Total (95% CI)**			**203**			**203**	**100.0%**	**-0.30 [-0.50, -0.11]**

Heterogeneity: Not applicable
Test for overall effect: Z = 3.05 (P = 0.002)

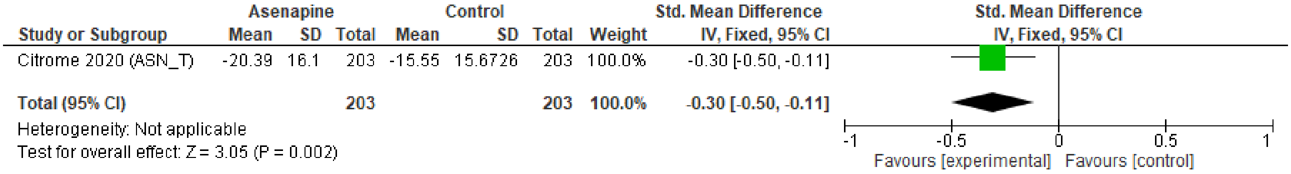

Olanzapine

Shows the forest plots of the standardized mean difference in the changes of symptom score from baseline to week 1, 2, 3, 4, 5, 6, 26 (green squares) with a 95% CI (bars) between olanzapine and placebo. The black diamonds are the summary effect estimates, which forms the dots in figure 3D.

Week 1:

	Experimental			Control				Std. Mean Difference
**Study or Subgroup**	**Mean**	**SD**	**Total**	**Mean**	**SD**	**Total**	**Weight**	**IV, Fixed, 95% CI**
Beasley 1996_2 (OLZ)	-3.46	12.5	49	-2.56	12.3	49	6.9%	-0.07 [-0.47, 0.32]
Corrigan 2004 (OLZ)	-8.56	14.6215	93	-4.23	14.6215	85	12.3%	-0.29 [-0.59, 0.00]
Egan 2013 (OLZ)	-7.15	10.3977	45	-4.68	10.4215	78	7.9%	-0.24 [-0.60, 0.13]
Meltzer 2011 (OLZ)	-10.45	20.9	121	-6.82	22.4219	114	16.4%	-0.17 [-0.42, 0.09]
Mosolov 2011 (OLZ)	-3.81	14.1109	34	1.91	20.24	63	6.1%	-0.31 [-0.73, 0.11]
Potkin 2020 (OLZ)	-7.95	9.5934	132	-7.19	8.7647	133	18.5%	-0.08 [-0.32, 0.16]
Schmidt 2012 (OLZ)	-7.5	11.1866	93	-3.84	10.8454	99	13.2%	-0.33 [-0.62, -0.05]
Shen 2008 (OLZ)	-10.2	13.7712	68	-2.55	14.0186	68	9.2%	-0.55 [-0.89, -0.20]
Shen 2014 (OLZ)	-9.7	13.2712	71	-1.62	13.2712	71	9.5%	-0.61 [-0.94, -0.27]
**Total (95% CI)**			**706**			**760**	**100.0%**	**-0.27 [-0.38, -0.17]**

Heterogeneity: Chi^2 = 10.51, df = 8 (P = 0.23); I^2 = 24%
Test for overall effect: Z = 5.16 (P < 0.00001)

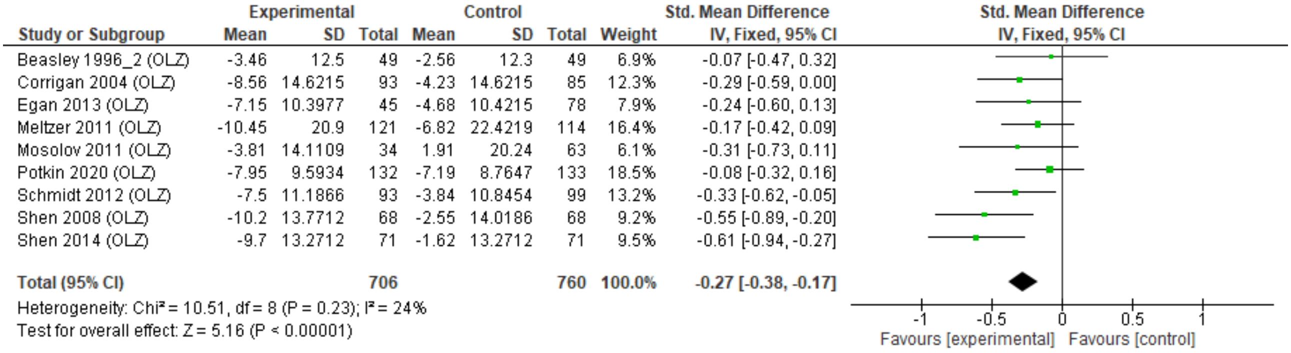

Week 2:

	Experimental			Control				Std. Mean Difference
**Study or Subgroup**	**Mean**	**SD**	**Total**	**Mean**	**SD**	**Total**	**Weight**	**IV, Fixed, 95% CI**
Beasley 1996_2 (OLZ)	-8.1	12.5	47	-2.75	12.3	45	6.4%	-0.43 [-0.84, -0.01]
Corrigan 2004 (OLZ)	-18.14	19.0761	93	-8.56	19.0761	85	12.2%	-0.50 [-0.80, -0.20]
Egan 2013 (OLZ)	-11.42	15.0935	45	-8.01	15.2789	78	8.1%	-0.22 [-0.59, 0.15]
Meltzer 2011 (OLZ)	-15.77	20.9	121	-10.23	22.4219	114	16.6%	-0.26 [-0.51, 0.00]
Mosolov 2011 (OLZ)	-10.72	18.2509	34	4.44	22.4624	63	5.9%	-0.71 [-1.14, -0.28]
Potkin 2020 (OLZ)	-13.56	12.2934	132	-11.41	12.3398	133	18.8%	-0.17 [-0.42, 0.07]
Schmidt 2012 (OLZ)	-13.41	13.6458	93	-4.74	13.7806	99	13.0%	-0.63 [-0.92, -0.34]
Shen 2008 (OLZ)	-10.14	15.4204	68	-2.78	15.6678	68	9.4%	-0.47 [-0.81, -0.13]
Shen 2014 (OLZ)	-9.95	14.3245	71	-1.47	15.1671	71	9.7%	-0.57 [-0.91, -0.24]
**Total (95% CI)**			**704**			**756**	**100.0%**	**-0.40 [-0.51, -0.30]**

Heterogeneity: Chi^2 = 11.54, df = 8 (P = 0.17); I^2 = 31%
Test for overall effect: Z = 7.59 (P < 0.00001)

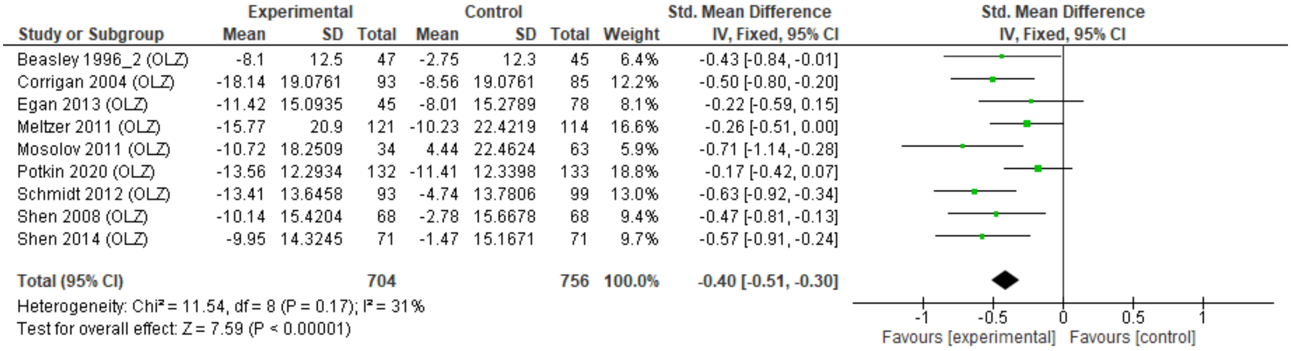

Week 3:

	Experimental			Control				Std. Mean Difference
**Study or Subgroup**	**Mean**	**SD**	**Total**	**Mean**	**SD**	**Total**	**Weight**	**IV, Fixed, 95% CI**
Beasley 1996_2 (OLZ)	-7.03	12.5	43	-2.31	12.3	44	6.2%	-0.38 [-0.80, 0.05]
Corrigan 2004 (OLZ)	-24.18	19.076	93	-13.8	19.076	85	12.3%	-0.54 [-0.84, -0.24]
Egan 2013 (OLZ)	-14.12	15.1605	45	-10.81	17.8402	78	8.2%	-0.19 [-0.56, 0.17]
Meltzer 2011 (OLZ)	-20.88	20.9	121	-11.32	22.4219	114	16.5%	-0.44 [-0.70, -0.18]
Mosolov 2011 (OLZ)	-15.11	21.6911	34	7.12	25.9548	63	5.8%	-0.90 [-1.33, -0.46]
Potkin 2020 (OLZ)	-18.53	13.0976	132	-13.34	14.4734	133	18.8%	-0.37 [-0.62, -0.13]
Schmidt 2012 (OLZ)	-18.43	15.6709	93	-6.81	17.0143	99	13.0%	-0.71 [-1.00, -0.41]
Shen 2008 (OLZ)	-12.24	18.2241	68	-3.06	18.2241	68	9.5%	-0.50 [-0.84, -0.16]
Shen 2014 (OLZ)	-11.9	17.1472	71	-1.7	17.737	71	9.8%	-0.58 [-0.92, -0.25]
**Total (95% CI)**			**700**			**755**	**100.0%**	**-0.50 [-0.60, -0.39]**

Heterogeneity: Chi^2 = 9.62, df = 8 (P = 0.29); I^2 = 17%
Test for overall effect: Z = 9.27 (P < 0.00001)

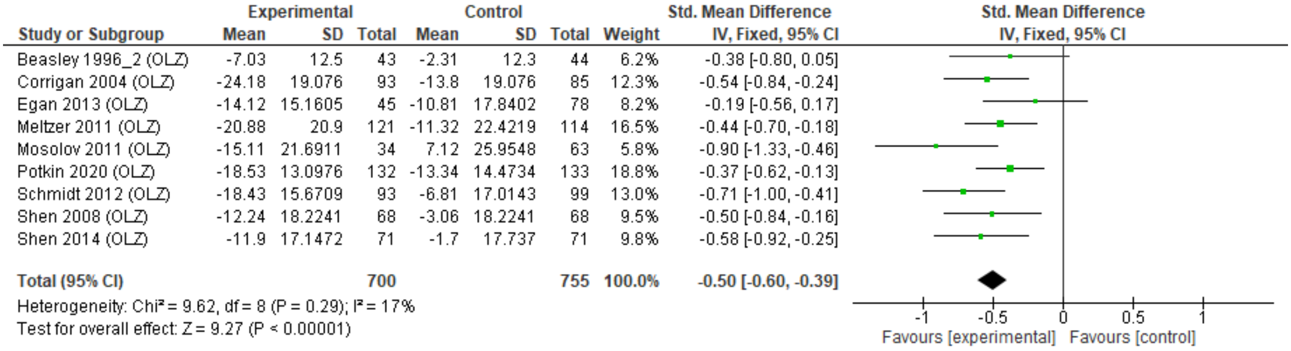

Week 4:

Study or Subgroup	Experimental Mean	Experimental SD	Experimental Total	Control Mean	Control SD	Control Total	Weight	Std. Mean Difference IV, Random, 95% CI
Beasley 1996_2 (OLZ)	-7.44	12.5	42	-2.09	12.3	42	6.3%	-0.43 [-0.86, 0.01]
Bugarski-Kirola 2014 (OLZ)	-14.9	16.6749	61	-11.9	16.8876	79	8.5%	-0.18 [-0.51, 0.16]
Corrigan 2004 (OLZ)	-30.23	19.076	93	-17.13	19.076	85	9.3%	-0.68 [-0.99, -0.38]
Egan 2013 (OLZ)	-17	19.5209	45	-12.7	19.8715	78	7.7%	-0.22 [-0.58, 0.15]
Kinnon 2011 (OLZ)	-20.68	24.2519	62	-14.6	24.2998	122	9.2%	-0.25 [-0.56, 0.06]
Litman 2014 (OLZ)	-8.24	14.84	22	4.82	14.99	41	4.7%	-0.86 [-1.40, -0.32]
Meltzer 2011 (OLZ)	-24.59	20.9	121	-12.91	22.4219	114	10.6%	-0.54 [-0.80, -0.28]
Mosolov 2011 (OLZ)	-19.13	22.3325	34	8.16	28.0185	63	6.1%	-1.03 [-1.48, -0.59]
Potkin 2020 (OLZ)	-22.8	14.9359	132	-17.5	14.9923	133	11.2%	-0.35 [-0.60, -0.11]
Schmidt 2012 (OLZ)	-20.74	17.3586	93	-7.41	18.8053	99	9.6%	-0.73 [-1.03, -0.44]
Shen 2008 (OLZ)	-14.16	18.9663	68	-3.74	19.2137	68	8.3%	-0.54 [-0.89, -0.20]
Shen 2014 (OLZ)	-14.2	17.9898	71	-2.23	18.369	71	8.4%	-0.65 [-0.99, -0.32]
**Total (95% CI)**			**844**			**995**	**100.0%**	**-0.52 [-0.65, -0.38]**

Heterogeneity: Tau² = 0.03; Chi² = 22.03, df = 11 (P = 0.02); I² = 50%
Test for overall effect: Z = 7.40 (P < 0.00001)

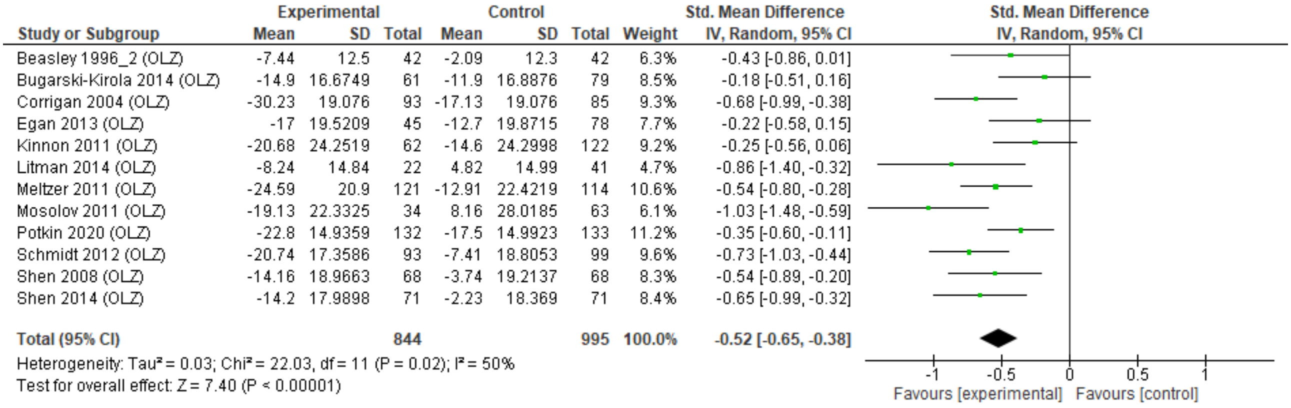

Week 5:

Study or Subgroup	Experimental Mean	Experimental SD	Experimental Total	Control Mean	Control SD	Control Total	Weight	Std. Mean Difference IV, Fixed, 95% CI
Beasley 1996_2 (OLZ)	-12.53	12.5	22	-8.31	12.3	14	3.9%	-0.33 [-1.01, 0.34]
Corrigan 2004 (OLZ)	-35.57	19.076	93	-19.75	19.076	85	19.0%	-0.83 [-1.13, -0.52]
Meltzer 2011 (OLZ)	-27.19	20.9	121	-14.91	22.4219	114	26.2%	-0.57 [-0.83, -0.30]
Schmidt 2012 (OLZ)	-21.87	17.3104	93	-5.94	19.651	99	20.4%	-0.86 [-1.15, -0.56]
Shen 2008 (OLZ)	-16.94	20.1208	68	-3.8	20.1208	68	15.0%	-0.65 [-0.99, -0.30]
Shen 2014 (OLZ)	-16.76	19.1695	71	-2.61	19.2959	71	15.4%	-0.73 [-1.07, -0.39]
**Total (95% CI)**			**468**			**451**	**100.0%**	**-0.70 [-0.84, -0.57]**

Heterogeneity: Chi² = 3.98, df = 5 (P = 0.55); I² = 0%
Test for overall effect: Z = 10.31 (P < 0.00001)

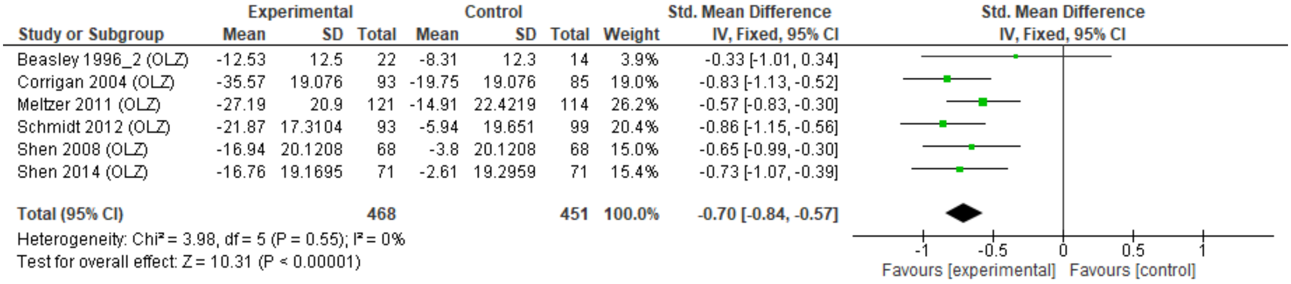

Week 6:

Study or Subgroup	Experimental Mean	Experimental SD	Experimental Total	Control Mean	Control SD	Control Total	Weight	Std. Mean Difference IV, Fixed, 95% CI
Beasley 1996_1 (OLZ)	-15.2	16.1	65	-3.1	17.5	62	6.8%	-0.72 [-1.08, -0.36]
Beasley 1996_2 (OLZ)	-15.6	12.5	19	-10.47	12.3	9	1.4%	-0.40 [-1.20, 0.40]
Corrigan 2004 (OLZ)	-31.5	19.076	93	-12.6	19.076	85	9.0%	-0.99 [-1.30, -0.67]
Davidson 2007 (OLZ)	-18.1	20.3	126	-2.8	20.9	120	13.0%	-0.74 [-1.00, -0.48]
Kane 2007 (OLZ)	-19.9	19	128	-4.1	23.2	126	13.5%	-0.74 [-1.00, -0.49]
Landbloom 2017 (OLZ)	-21.6	15.563	45	-16.2	17.0143	99	6.9%	-0.32 [-0.68, 0.03]
Marder 2007 (OLZ)	-18.4	19.9	105	-8	21.5	105	11.5%	-0.50 [-0.78, -0.23]
Meltzer 2011 (OLZ)	-28.7	20.9	121	-16	22.4219	114	12.8%	-0.58 [-0.85, -0.32]
Schmidt 2012 (OLZ)	-22.9	18.1301	93	-6.4	20.2977	99	10.0%	-0.85 [-1.15, -0.56]
Shen 2008 (OLZ)	-15.2	20.7805	68	-4.43	20.698	68	7.5%	-0.52 [-0.86, -0.17]
Shen 2014 (OLZ)	-14.7	20.2228	71	-2.7	20.5598	71	7.7%	-0.59 [-0.92, -0.25]
**Total (95% CI)**			**934**			**958**	**100.0%**	**-0.66 [-0.76, -0.57]**

Heterogeneity: Chi² = 13.06, df = 10 (P = 0.22); I² = 23%
Test for overall effect: Z = 13.91 (P < 0.00001)

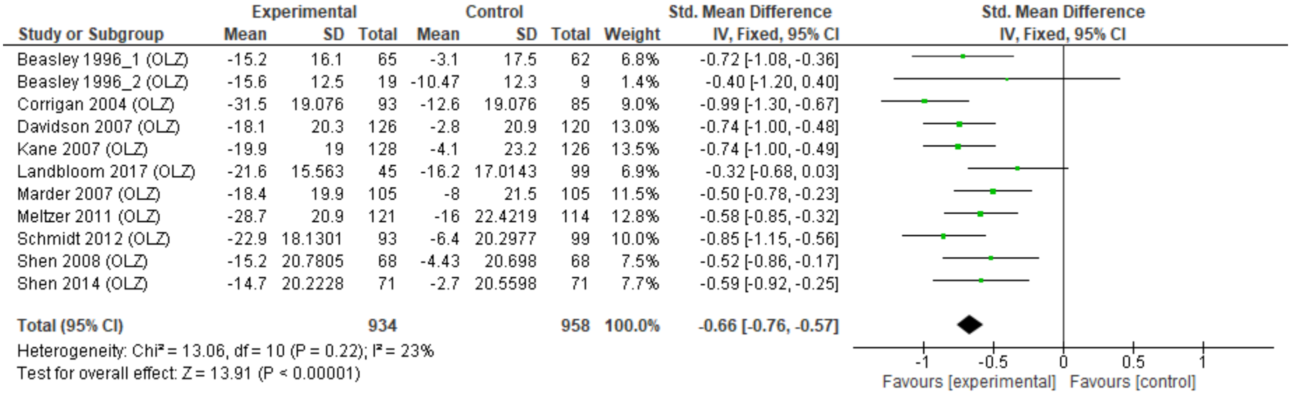

Week 26:

Study or Subgroup	Experimental Mean	Experimental SD	Experimental Total	Control Mean	Control SD	Control Total	Weight	Std. Mean Difference IV, Fixed, 95% CI
Lecrubier 2006 (OLZ)	-15.5	24.5	70	-12.6	24	34	100.0%	-0.12 [-0.53, 0.29]
**Total (95% CI)**			**70**			**34**	**100.0%**	**-0.12 [-0.53, 0.29]**

Heterogeneity: Not applicable
Test for overall effect: Z = 0.57 (P = 0.57)

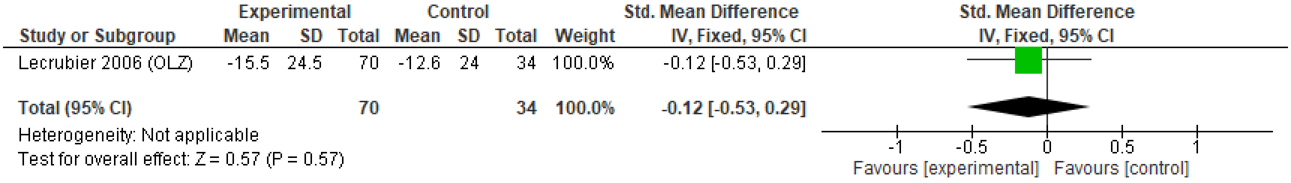

Quetiapine

Shows the forest plots of the standardized mean difference in the changes of symptom score from baseline to week 1, 2, 3, 4, 5, 6 (green squares) with a 95% CI (bars) between quetiapine and placebo. The black diamonds are the summary effect estimates, which forms the dots in figure 3E.

Week 1:

	Experimental			Control				Std. Mean Difference
Study or Subgroup	Mean	SD	Total	Mean	SD	Total	Weight	IV, Fixed, 95% CI
Borrison 1996 (QTP)	-3.16	10.083	53	-0.7	10.1922	53	15.2%	-0.24 [-0.62, 0.14]
Canuso 2009 (QTP)	-12.88	15.9962	150	-11.6	15.9962	79	29.9%	-0.08 [-0.35, 0.19]
Fabre 1995 (QTP)	-9.01	8.5	8	-5.59	13.2	4	1.5%	-0.31 [-1.52, 0.90]
Potkin 2006 (QTP)	-13.88	17.028	146	-16.38	17.028	68	26.8%	0.15 [-0.14, 0.43]
Small 1997 (QTP)	-2.41	9.9753	92	-0.39	10.0233	92	26.5%	-0.20 [-0.49, 0.09]
**Total (95% CI)**			**449**			**296**	**100.0%**	**-0.08 [-0.23, 0.07]**

Heterogeneity: Chi² = 3.86, df = 4 (P = 0.42); I² = 0%
Test for overall effect: Z = 1.04 (P = 0.30)

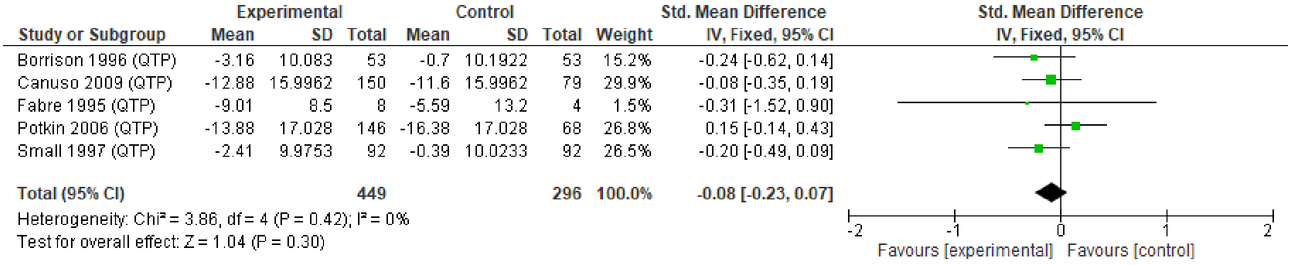

Week 2:

	Experimental			Control				Std. Mean Difference
Study or Subgroup	Mean	SD	Total	Mean	SD	Total	Weight	IV, Fixed, 95% CI
Borrison 1996 (QTP)	-5.6	14.997	53	-0.5	13.8322	53	16.6%	-0.35 [-0.73, 0.03]
Canuso 2009 (QTP)	-20.26	14.9351	134	-21.12	14.9351	68	28.7%	0.06 [-0.23, 0.35]
Fabre 1995 (QTP)	-17.05	8.5	8	-1.22	13.2	4	1.3%	-1.44 [-2.83, -0.05]
Potkin 2006 (QTP)	-23.8	15.6222	129	-21.38	15.6222	56	24.8%	-0.15 [-0.47, 0.16]
Small 1997 (QTP)	-4.57	12.2773	92	0.07	12.8528	92	28.7%	-0.37 [-0.66, -0.08]
**Total (95% CI)**			**416**			**273**	**100.0%**	**-0.20 [-0.36, -0.05]**

Heterogeneity: Chi² = 7.99, df = 4 (P = 0.09); I² = 50%
Test for overall effect: Z = 2.56 (P = 0.01)

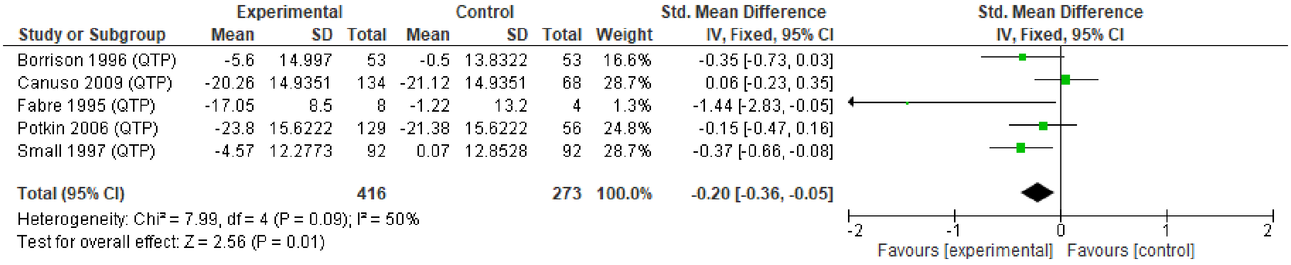

Week 3:

	Experimental			Control				Std. Mean Difference
Study or Subgroup	Mean	SD	Total	Mean	SD	Total	Weight	IV, Fixed, 95% CI
Borrison 1996 (QTP)	-7	15.2882	53	-1.97	15.179	53	35.7%	-0.33 [-0.71, 0.06]
Fabre 1995 (QTP)	-20.9	8.5	8	-4.7	13.2	4	2.7%	-1.47 [-2.87, -0.08]
Small 1997 (QTP)	-6.43	14.6752	92	-0.71	14.1957	92	61.6%	-0.39 [-0.69, -0.10]
**Total (95% CI)**			**153**			**149**	**100.0%**	**-0.40 [-0.63, -0.17]**

Heterogeneity: Chi² = 2.40, df = 2 (P = 0.30); I² = 17%
Test for overall effect: Z = 3.42 (P = 0.0006)

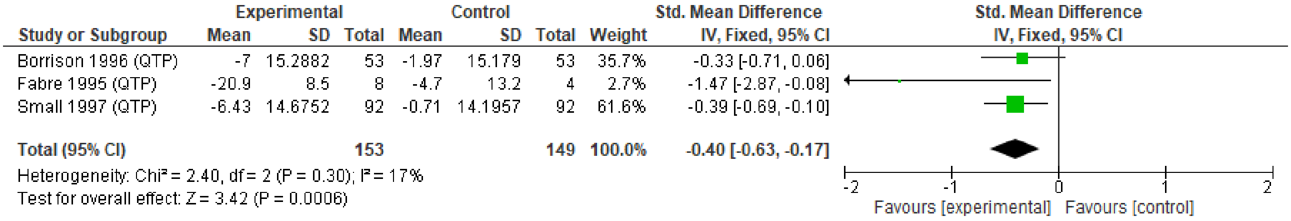

Week 4:

	Experimental			Control				Std. Mean Difference
Study or Subgroup	Mean	SD	Total	Mean	SD	Total	Weight	IV, Fixed, 95% CI
Borrison 1996 (QTP)	-8.4	15.2882	53	-1.8	15.2882	53	36.7%	-0.43 [-0.81, -0.04]
Small 1997 (QTP)	-7.71	13.9079	92	-0.51	14.6752	92	63.3%	-0.50 [-0.80, -0.21]
**Total (95% CI)**			**145**			**145**	**100.0%**	**-0.47 [-0.71, -0.24]**

Heterogeneity: Chi² = 0.09, df = 1 (P = 0.77); I² = 0%
Test for overall effect: Z = 3.98 (P < 0.0001)

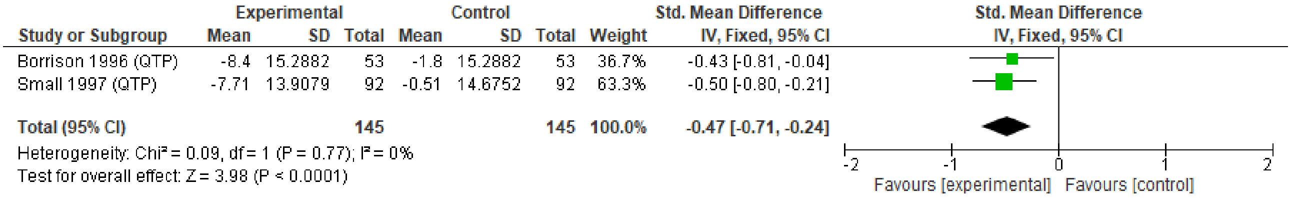

Week 5:

	Experimental			Control				Std. Mean Difference
Study or Subgroup	Mean	SD	Total	Mean	SD	Total	Weight	IV, Fixed, 95% CI
Borrison 1996 (QTP)	-8.7	16.6715	53	-1.3	16.7443	53	36.7%	-0.44 [-0.83, -0.05]
Small 1997 (QTP)	-8.02	15.1548	92	-0.32	14.6752	92	63.3%	-0.51 [-0.81, -0.22]
**Total (95% CI)**			**145**			**145**	**100.0%**	**-0.49 [-0.72, -0.25]**

Heterogeneity: Chi² = 0.09, df = 1 (P = 0.76); I² = 0%
Test for overall effect: Z = 4.08 (P < 0.0001)

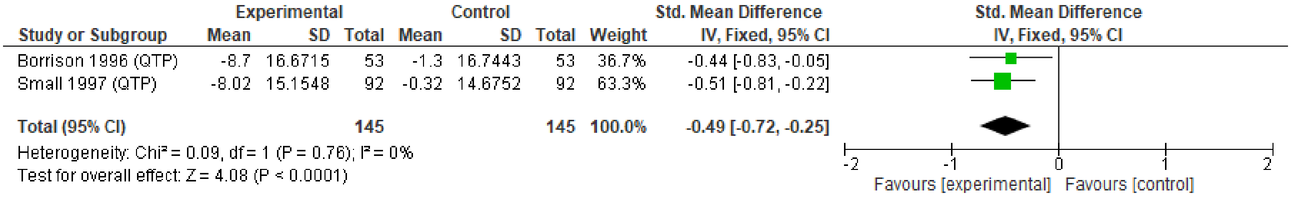

Week 6:

Study or Subgroup	Experimental Mean	Experimental SD	Experimental Total	Control Mean	Control SD	Control Total	Weight	Std. Mean Difference IV, Fixed, 95% CI
Arvanitis 1997 (QTP)	-6.33	14.7058	53	1.71	14.7113	51	13.0%	-0.54 [-0.93, -0.15]
Borrison 1996 (QTP)	-8.1	17.3995	53	-2.1	17.3995	53	13.6%	-0.34 [-0.73, 0.04]
Kahn 2007 (QTP, QTPXR)	-26.6	20.518	119	-18.8	20.518	115	29.8%	-0.38 [-0.64, -0.12]
Lindenmayer 2008 (QTP,QTPXR)	-6.97	17.4	80	-5.19	16.4	78	20.5%	-0.10 [-0.42, 0.21]
Small 1997 (QTP)	-8.7	15.7303	92	-1	15.4426	92	23.2%	-0.49 [-0.79, -0.20]
**Total (95% CI)**			**397**			**389**	**100.0%**	**-0.37 [-0.51, -0.22]**

Heterogeneity: Chi² = 4.20, df = 4 (P = 0.38); I² = 5%
Test for overall effect: Z = 5.07 (P < 0.00001)

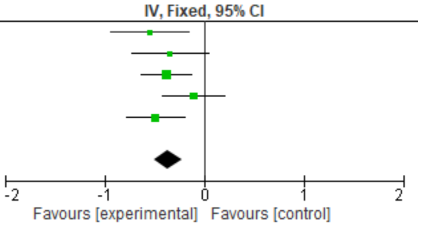

Zotepine

Shows the forest plots of the standardized mean difference in the changes of symptom score from baseline to week 1, 2, 4, 6, 8, 16, 20, 26 (green squares) with a 95% CI (bars) between zotepine and placebo. The black diamonds are the summary effect estimates, which forms the dots in figure 3F.

Week 1:

Study or Subgroup	Experimental Mean	Experimental SD	Experimental Total	Control Mean	Control SD	Control Total	Weight	Std. Mean Difference IV, Fixed, 95% CI
Cooper 2000_1 (ZOT)	-4.6	7.4	53	-1.4	7.9	53	100.0%	-0.42 [-0.80, -0.03]
**Total (95% CI)**			**53**			**53**	**100.0%**	**-0.42 [-0.80, -0.03]**

Heterogeneity: Not applicable
Test for overall effect: Z = 2.11 (P = 0.03)

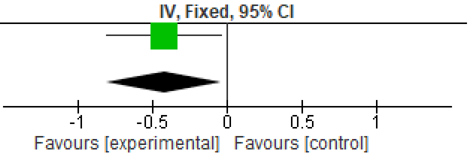

Week 2:

Study or Subgroup	Experimental Mean	Experimental SD	Experimental Total	Control Mean	Control SD	Control Total	Weight	Std. Mean Difference IV, Fixed, 95% CI
Cooper 2000_1 (ZOT)	-9	10.8	53	-2.4	11.9	53	46.1%	-0.58 [-0.97, -0.19]
Cooper 2000_2 (ZOT)	-3.38	15	61	-0.98	17.1	58	53.9%	-0.15 [-0.51, 0.21]
**Total (95% CI)**			**114**			**111**	**100.0%**	**-0.35 [-0.61, -0.08]**

Heterogeneity: Chi² = 2.51, df = 1 (P = 0.11); I² = 60%
Test for overall effect: Z = 2.57 (P = 0.01)

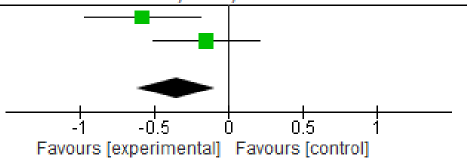

Week 4:

Study or Subgroup	Experimental Mean	Experimental SD	Experimental Total	Control Mean	Control SD	Control Total	Weight	Std. Mean Difference IV, Fixed, 95% CI
Cooper 2000_1 (ZOT)	-11.8	11.9	53	-4.5	14.5	53	46.2%	-0.55 [-0.93, -0.16]
Cooper 2000_2 (ZOT)	-4.58	15	61	-3.2	17.1	58	53.8%	-0.09 [-0.45, 0.27]
**Total (95% CI)**			**114**			**111**	**100.0%**	**-0.30 [-0.56, -0.03]**

Heterogeneity: Chi² = 2.92, df = 1 (P = 0.09); I² = 66%
Test for overall effect: Z = 2.22 (P = 0.03)

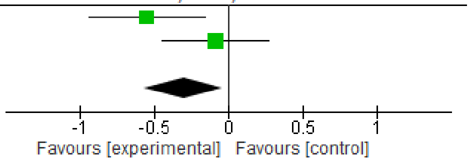

Week 6:

Study or Subgroup	Experimental Mean	Experimental SD	Experimental Total	Control Mean	Control SD	Control Total	Weight	Std. Mean Difference IV, Fixed, 95% CI
Cooper 2000_1 (ZOT)	-14.8	13.7	53	-3.7	14.9	53	100.0%	-0.77 [-1.17, -0.37]
**Total (95% CI)**			**53**			**53**	**100.0%**	**-0.77 [-1.17, -0.37]**

Heterogeneity: Not applicable
Test for overall effect: Z = 3.82 (P = 0.0001)

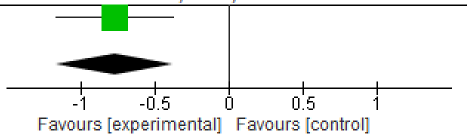

Week 8:

Study or Subgroup	Experimental Mean	SD	Total	Control Mean	SD	Total	Weight	Std. Mean Difference IV, Fixed, 95% CI
Cooper 2000_1 (ZOT)	-16.8	14.9	53	-2.9	16.3	53	33.0%	-0.88 [-1.28, -0.48]
Cooper 2000_2 (ZOT)	-6.68	15	61	-1.59	17.1	58	40.3%	-0.31 [-0.68, 0.05]
Moller 2004 (ZOT)	-13.3	18.2	38	-8.4	15.4	41	26.8%	-0.29 [-0.73, 0.16]
**Total (95% CI)**			**152**			**152**	**100.0%**	**-0.50 [-0.73, -0.27]**

Heterogeneity: Chi² = 5.42, df = 2 (P = 0.07); I² = 63%
Test for overall effect: Z = 4.23 (P < 0.0001)

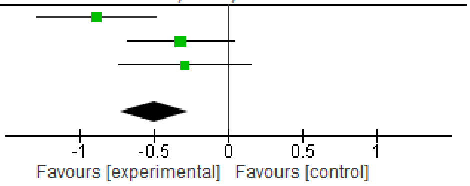

Week 16:

Study or Subgroup	Experimental Mean	SD	Total	Control Mean	SD	Total	Weight	Std. Mean Difference IV, Fixed, 95% CI
Cooper 2000_2 (ZOT)	-5.52	15	61	0.9	17.1	58	100.0%	-0.40 [-0.76, -0.03]
**Total (95% CI)**			**61**			**58**	**100.0%**	**-0.40 [-0.76, -0.03]**

Heterogeneity: Not applicable
Test for overall effect: Z = 2.14 (P = 0.03)

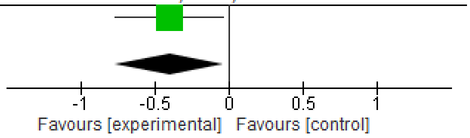

Week 20:

Study or Subgroup	Experimental Mean	SD	Total	Control Mean	SD	Total	Weight	Std. Mean Difference IV, Fixed, 95% CI
Cooper 2000_2 (ZOT)	-5.71	15	61	1.27	17.1	58	100.0%	-0.43 [-0.80, -0.07]
**Total (95% CI)**			**61**			**58**	**100.0%**	**-0.43 [-0.80, -0.07]**

Heterogeneity: Not applicable
Test for overall effect: Z = 2.33 (P = 0.02)

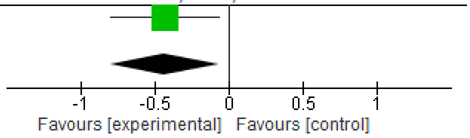

Week 26:

Study or Subgroup	Experimental Mean	SD	Total	Control Mean	SD	Total	Weight	Std. Mean Difference IV, Fixed, 95% CI
Cooper 2000_2 (ZOT)	-5.7	15	61	1.8	17.1	58	100.0%	-0.46 [-0.83, -0.10]
**Total (95% CI)**			**61**			**58**	**100.0%**	**-0.46 [-0.83, -0.10]**

Heterogeneity: Not applicable
Test for overall effect: Z = 2.50 (P = 0.01)

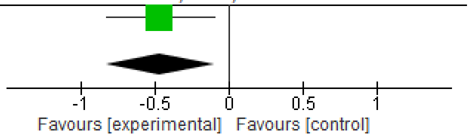

Olanzapine, Long-acting Injections

Shows the forest plots of the standardized mean difference in the changes of symptom score from baseline to week 1, 2, 3, 4, 5, 6, 7, 8 (green squares) with a 95% CI (bars) between long acting olanzapine and placebo. The black diamonds are the summary effect estimates, which forms the dots in figure 3G.

Week 1:

Study or Subgroup	Olanzapine LAI Mean	SD	Total	Placebo Mean	SD	Total	Weight	Std. Mean Difference IV, Fixed, 95% CI
Lauriello 2008 (OLZLAI)	-14.9	24.9	98	-9.35	23	98	100.0%	-0.23 [-0.51, 0.05]
**Total (95% CI)**			**98**			**98**	**100.0%**	**-0.23 [-0.51, 0.05]**

Heterogeneity: Not applicable
Test for overall effect: Z = 1.61 (P = 0.11)

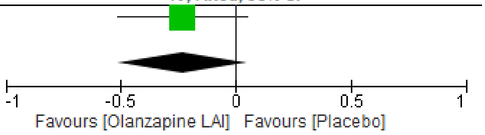

Week 2:

Study or Subgroup	Olanzapin LAI Mean	Olanzapin LAI SD	Olanzapin LAI Total	Placebo Mean	Placebo SD	Placebo Total	Weight	Std. Mean Difference IV, Fixed, 95% CI
Lauriello 2008 (OLZLAI)	-19.68	24.9	98	-10.91	23	98	100.0%	-0.36 [-0.65, -0.08]
**Total (95% CI)**			**98**			**98**	**100.0%**	**-0.36 [-0.65, -0.08]**
Heterogeneity: Not applicable								
Test for overall effect: Z = 2.53 (P = 0.01)								

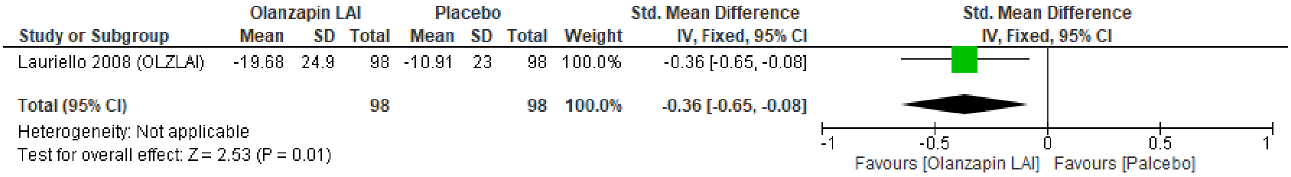

Week 3:

Study or Subgroup	Olanzapin LAI Mean	Olanzapin LAI SD	Olanzapin LAI Total	Placebo Mean	Placebo SD	Placebo Total	Weight	Std. Mean Difference IV, Fixed, 95% CI
Lauriello 2008 (OLZLAI)	-22.31	24.9	98	-10.71	23	98	100.0%	-0.48 [-0.77, -0.20]
**Total (95% CI)**			**98**			**98**	**100.0%**	**-0.48 [-0.77, -0.20]**
Heterogeneity: Not applicable								
Test for overall effect: Z = 3.33 (P = 0.0009)								

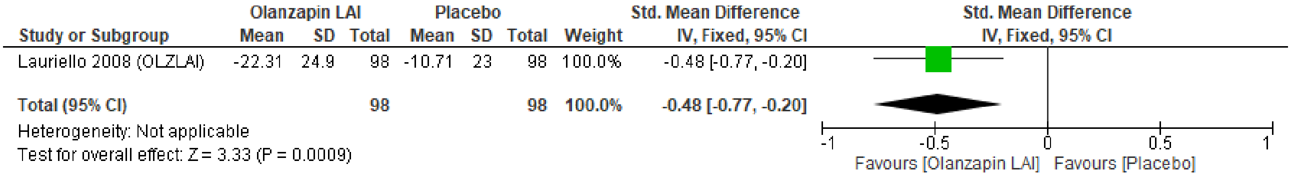

Week 4:

Study or Subgroup	Olanzapin LAI Mean	Olanzapin LAI SD	Olanzapin LAI Total	Placebo Mean	Placebo SD	Placebo Total	Weight	Std. Mean Difference IV, Fixed, 95% CI
Lauriello 2008 (OLZLAI)	-22.69	24.9	98	-8.86	23	98	100.0%	-0.57 [-0.86, -0.29]
**Total (95% CI)**			**98**			**98**	**100.0%**	**-0.57 [-0.86, -0.29]**
Heterogeneity: Not applicable								
Test for overall effect: Z = 3.94 (P < 0.0001)								

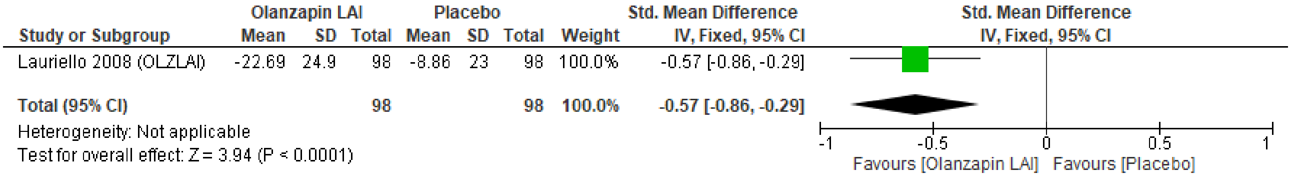

Week 5:

Study or Subgroup	Olanzapin LAI Mean	Olanzapin LAI SD	Olanzapin LAI Total	Placebo Mean	Placebo SD	Placebo Total	Weight	Std. Mean Difference IV, Fixed, 95% CI
Lauriello 2008 (OLZLAI)	-23.38	24.9	98	-8.86	23	98	100.0%	-0.60 [-0.89, -0.32]
**Total (95% CI)**			**98**			**98**	**100.0%**	**-0.60 [-0.89, -0.32]**
Heterogeneity: Not applicable								
Test for overall effect: Z = 4.13 (P < 0.0001)								

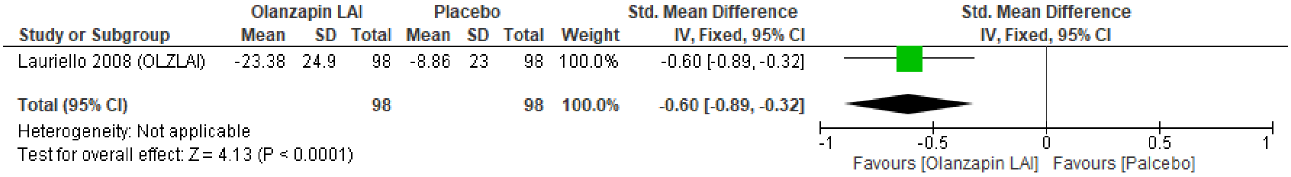

Week 6:

Study or Subgroup	Olanzapin LAI Mean	Olanzapin LAI SD	Olanzapin LAI Total	Placebo Mean	Placebo SD	Placebo Total	Weight	Std. Mean Difference IV, Fixed, 95% CI
Lauriello 2008 (OLZLAI)	-24.84	24.9	98	-8.87	23	98	100.0%	-0.66 [-0.95, -0.38]
**Total (95% CI)**			**98**			**98**	**100.0%**	**-0.66 [-0.95, -0.38]**
Heterogeneity: Not applicable								
Test for overall effect: Z = 4.52 (P < 0.00001)								

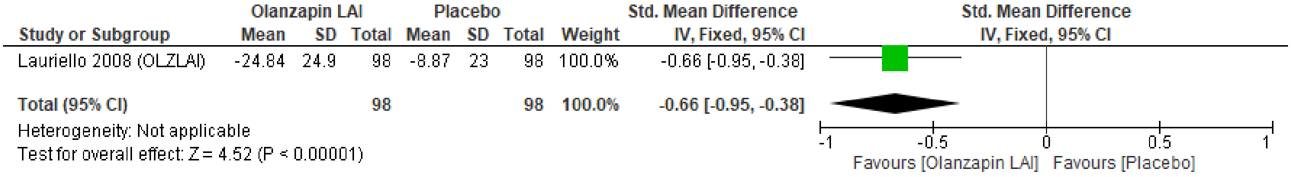

Week 7:

Study or Subgroup	Olanzapin LAI Mean	Olanzapin LAI SD	Olanzapin LAI Total	Placebo Mean	Placebo SD	Placebo Total	Weight	Std. Mean Difference IV, Fixed, 95% CI
Lauriello 2008 (OLZLAI)	-25.81	24.9	98	-8.57	23	98	100.0%	-0.72 [-1.01, -0.43]
**Total (95% CI)**			**98**			**98**	**100.0%**	**-0.72 [-1.01, -0.43]**
Heterogeneity: Not applicable								
Test for overall effect: Z = 4.86 (P < 0.00001)								

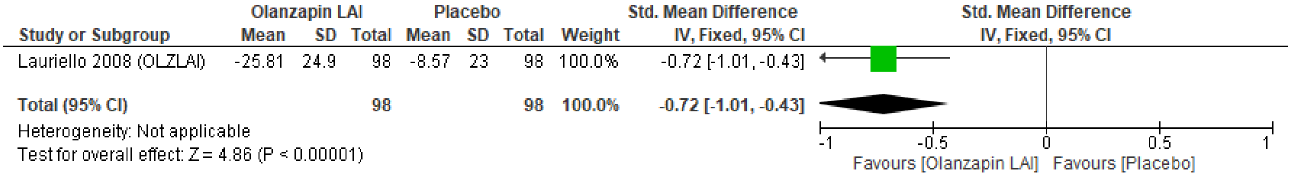

Week 8:

Study or Subgroup	Olanzapin LAI Mean	Olanzapin LAI SD	Olanzapin LAI Total	Placebo Mean	Placebo SD	Placebo Total	Weight	Std. Mean Difference IV, Fixed, 95% CI
Lauriello 2008 (OLZLAI)	-26.3	24.9	98	-8.5	23	98	100.0%	-0.74 [-1.03, -0.45]
**Total (95% CI)**			**98**			**98**	**100.0%**	**-0.74 [-1.03, -0.45]**
Heterogeneity: Not applicable								
Test for overall effect: Z = 5.01 (P < 0.00001)								

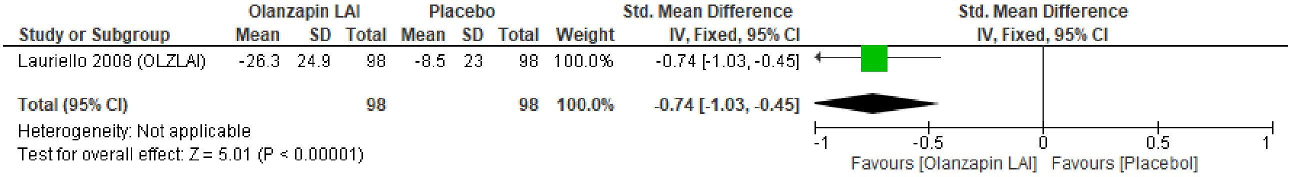

Quetiapine, Extended Release

Shows the forest plots of the standardized mean difference in the changes of symptom score from baseline to week 1, 2, 3, 4, 5, 6 (green squares) with a 95% CI (bars) between quetiapine extended release and placebo. The black diamonds are the summary effect estimates, which forms the dots in figure 3H.

Week 1:

	Experimental			Control				Std. Mean Difference
**Study or Subgroup**	**Mean**	**SD**	**Total**	**Mean**	**SD**	**Total**	**Weight**	**IV, Fixed, 95% CI**
Loebel 2013 (QTPXR)	-8.12	19.3866	116	-3.33	19.718	120	100.0%	-0.24 [-0.50, 0.01]
**Total (95% CI)**			**116**			**120**	**100.0%**	**-0.24 [-0.50, 0.01]**

Heterogeneity: Not applicable
Test for overall effect: Z = 1.87 (P = 0.06)

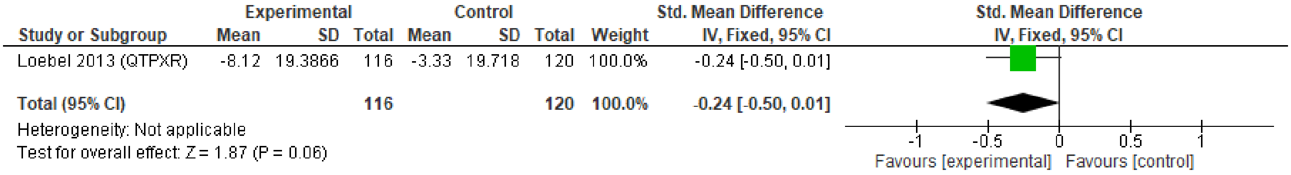

Week 2:

	Experimental			Control				Std. Mean Difference
**Study or Subgroup**	**Mean**	**SD**	**Total**	**Mean**	**SD**	**Total**	**Weight**	**IV, Fixed, 95% CI**
Loebel 2013 (QTPXR)	-14.86	19.3866	116	-5.8	19.718	120	100.0%	-0.46 [-0.72, -0.20]
**Total (95% CI)**			**116**			**120**	**100.0%**	**-0.46 [-0.72, -0.20]**

Heterogeneity: Not applicable
Test for overall effect: Z = 3.50 (P = 0.0005)

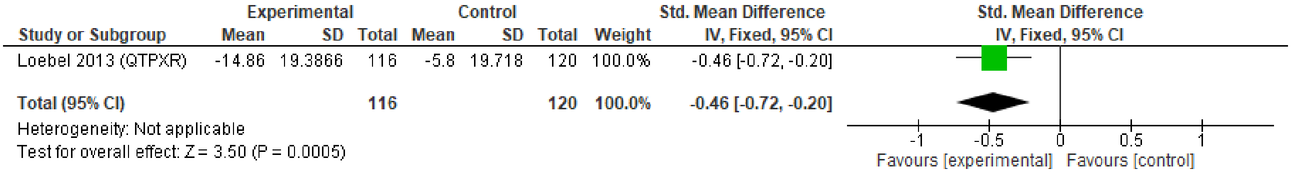

Week 3:

	Experimental			Control				Std. Mean Difference
**Study or Subgroup**	**Mean**	**SD**	**Total**	**Mean**	**SD**	**Total**	**Weight**	**IV, Fixed, 95% CI**
Loebel 2013 (QTPXR)	-19.28	19.3866	116	-7.1	19.718	120	100.0%	-0.62 [-0.88, -0.36]
**Total (95% CI)**			**116**			**120**	**100.0%**	**-0.62 [-0.88, -0.36]**

Heterogeneity: Not applicable
Test for overall effect: Z = 4.66 (P < 0.00001)

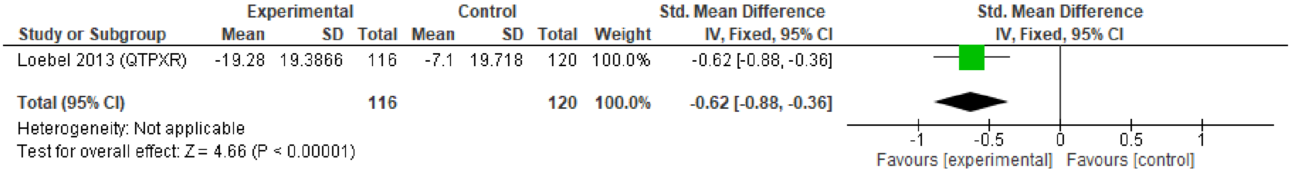

Week 4:

	Experimental			Control				Std. Mean Difference
**Study or Subgroup**	**Mean**	**SD**	**Total**	**Mean**	**SD**	**Total**	**Weight**	**IV, Fixed, 95% CI**
Loebel 2013 (QTPXR)	-22.97	19.3866	116	-8.12	19.718	120	100.0%	-0.76 [-1.02, -0.49]
**Total (95% CI)**			**116**			**120**	**100.0%**	**-0.76 [-1.02, -0.49]**

Heterogeneity: Not applicable
Test for overall effect: Z = 5.61 (P < 0.00001)

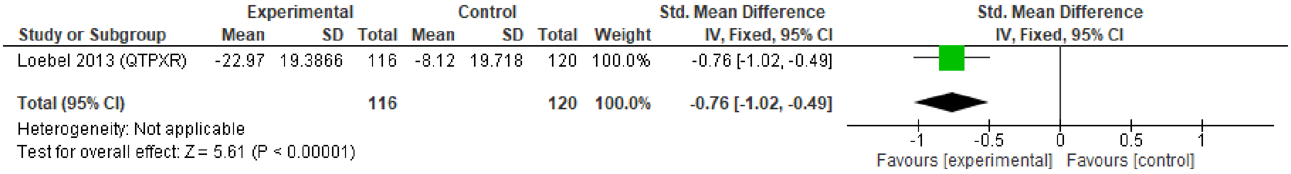

Week 5:

	Experimental			Control				Std. Mean Difference
**Study or Subgroup**	**Mean**	**SD**	**Total**	**Mean**	**SD**	**Total**	**Weight**	**IV, Fixed, 95% CI**
Loebel 2013 (QTPXR)	-25.36	19.3866	116	-8.55	19.718	120	100.0%	-0.86 [-1.12, -0.59]
**Total (95% CI)**			**116**			**120**	**100.0%**	**-0.86 [-1.12, -0.59]**

Heterogeneity: Not applicable
Test for overall effect: Z = 6.29 (P < 0.00001)

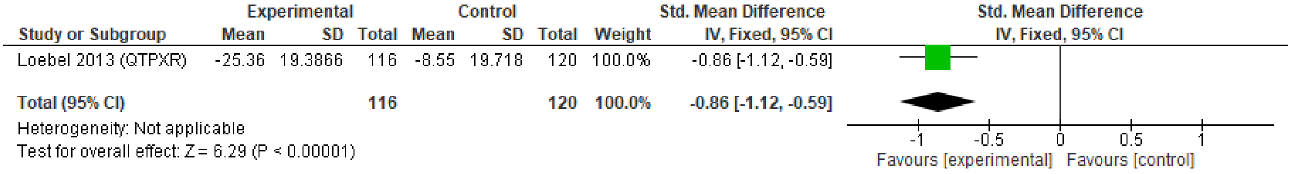

Week 6:

	Experimental			Control				Std. Mean Difference
**Study or Subgroup**	**Mean**	**SD**	**Total**	**Mean**	**SD**	**Total**	**Weight**	**IV, Random, 95% CI**
Kahn 2007 (QTP, QTPXR)	-31.3	28.5584	117	-18.8	28.5584	115	34.5%	-0.44 [-0.70, -0.18]
Lindenmayer 2008 (QTP,QTPXR)	-11.17	15.1	85	-5.19	16.4	78	31.5%	-0.38 [-0.69, -0.07]
Loebel 2013 (QTPXR)	-27.8	19.3866	116	-10.3	19.718	120	34.0%	-0.89 [-1.16, -0.62]
**Total (95% CI)**			**318**			**313**	**100.0%**	**-0.57 [-0.90, -0.25]**

Heterogeneity: Tau² = 0.06; Chi² = 8.01, df = 2 (P = 0.02); I² = 75%
Test for overall effect: Z = 3.49 (P = 0.0005)

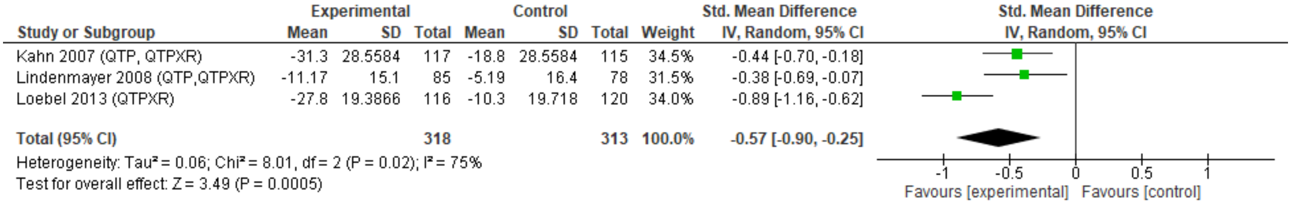

Supplemental 6 – Sensitivity analysis, sequentially data only

Asenapine sequentially data only

Shows the forest plots of the standardized mean difference in the changes of symptom score from baseline to week 1, 2, 3, 4, 5, 6 (green squares) with a 95% CI (bars) between asenapine and placebo. The black diamonds are the summary effect estimates. Only the sequential data are included, meaning studies that in addition to the baseline symptom score presented at least two symptom scores.

Week 1:

	Asenapine			Placebo				Std. Mean Difference
Study or Subgroup	Mean	SD	Total	Mean	SD	Total	Weight	IV, Fixed, 95% CI
Kane 2010 (ASN_S)	-7.99	10.0932	105	-6.19	9.8856	122	65.9%	-0.18 [-0.44, 0.08]
Potkin 2007 (ASN_S)	-6.41	7.5015	58	-3.99	6.9326	60	34.1%	-0.33 [-0.70, 0.03]
**Total (95% CI)**			**163**			**182**	**100.0%**	**-0.23 [-0.44, -0.02]**

Heterogeneity: Chi^2 = 0.45, df = 1 (P = 0.50); I^2 = 0%
Test for overall effect: Z = 2.14 (P = 0.03)

Std. Mean Difference IV, Fixed, 95% CI

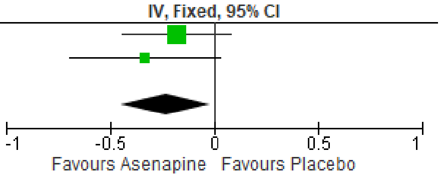

Week 2:

	Asenapine			Placebo				Std. Mean Difference
Study or Subgroup	Mean	SD	Total	Mean	SD	Total	Weight	IV, Fixed, 95% CI
Kane 2010 (ASN_S)	-12	13.1673	105	-9.41	12.9231	122	65.9%	-0.20 [-0.46, 0.06]
Potkin 2007 (ASN_S)	-11.32	15.6288	58	-5.62	15.6288	60	34.1%	-0.36 [-0.73, 0.00]
**Total (95% CI)**			**163**			**182**	**100.0%**	**-0.25 [-0.47, -0.04]**

Heterogeneity: Chi^2 = 0.52, df = 1 (P = 0.47); I^2 = 0%
Test for overall effect: Z = 2.34 (P = 0.02)

Std. Mean Difference IV, Fixed, 95% CI

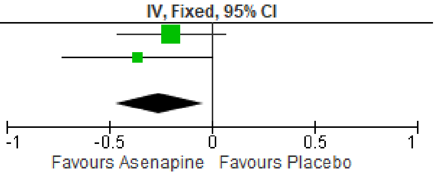

Week 3:

	Asenapine			Placebo				Std. Mean Difference
Study or Subgroup	Mean	SD	Total	Mean	SD	Total	Weight	IV, Fixed, 95% CI
Kane 2010 (ASN_S)	-13.87	14.1408	105	-10.93	14.6903	122	66.6%	-0.20 [-0.46, 0.06]
Potkin 2007 (ASN_S)	-17.01	17.0491	58	-6.41	17.0491	60	33.4%	-0.62 [-0.99, -0.25]
**Total (95% CI)**			**163**			**182**	**100.0%**	**-0.34 [-0.55, -0.13]**

Heterogeneity: Chi^2 = 3.22, df = 1 (P = 0.07); I^2 = 69%
Test for overall effect: Z = 3.13 (P = 0.002)

Std. Mean Difference IV, Fixed, 95% CI

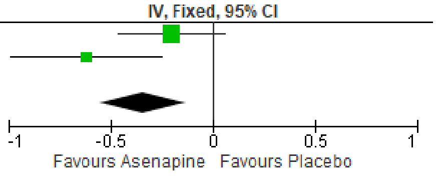

Week 4:

	Asenapine			Placebo				Std. Mean Difference
Study or Subgroup	Mean	SD	Total	Mean	SD	Total	Weight	IV, Fixed, 95% CI
Kane 2010 (ASN_S)	-14.52	15.1143	105	-12	15.2426	122	66.4%	-0.17 [-0.43, 0.10]
Potkin 2007 (ASN_S)	-16.87	19.4513	58	-6.62	19.4513	60	33.6%	-0.52 [-0.89, -0.16]
**Total (95% CI)**			**163**			**182**	**100.0%**	**-0.29 [-0.50, -0.07]**

Heterogeneity: Chi^2 = 2.42, df = 1 (P = 0.12); I^2 = 59%
Test for overall effect: Z = 2.63 (P = 0.009)

Std. Mean Difference IV, Fixed, 95% CI

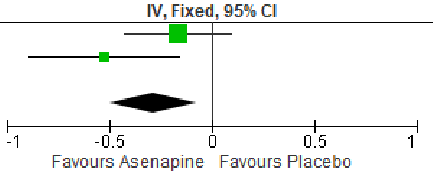

Week 5:

	Asenapine			Placebo				Std. Mean Difference
Study or Subgroup	Mean	SD	Total	Mean	SD	Total	Weight	IV, Fixed, 95% CI
Kane 2010 (ASN_S)	-17.41	16.0365	105	-13.32	16.5128	122	66.6%	-0.25 [-0.51, 0.01]
Potkin 2007 (ASN_S)	-15.94	17.9659	58	-4.77	17.9659	60	33.4%	-0.62 [-0.99, -0.25]
**Total (95% CI)**			**163**			**182**	**100.0%**	**-0.37 [-0.59, -0.16]**

Heterogeneity: Chi^2 = 2.53, df = 1 (P = 0.11); I^2 = 60%
Test for overall effect: Z = 3.42 (P = 0.0006)

Std. Mean Difference IV, Fixed, 95% CI

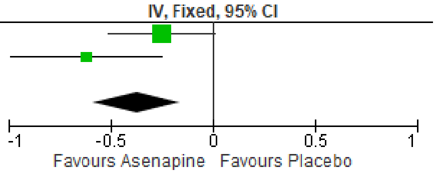

Week 6:

	Asenapine			Placebo				Std. Mean Difference
Study or Subgroup	Mean	SD	Total	Mean	SD	Total	Weight	IV, Fixed, 95% CI
Kane 2010 (ASN_S)	-19.44	17.1636	105	-14.56	18.0039	122	66.2%	-0.28 [-0.54, -0.01]
Potkin 2007 (ASN_S)	-15.9	20.1155	58	-5.3	20.1155	60	33.8%	-0.52 [-0.89, -0.16]
**Total (95% CI)**			**163**			**182**	**100.0%**	**-0.36 [-0.57, -0.15]**

Heterogeneity: Chi^2 = 1.16, df = 1 (P = 0.28); I^2 = 13%
Test for overall effect: Z = 3.30 (P = 0.0010)

Std. Mean Difference IV, Fixed, 95% CI

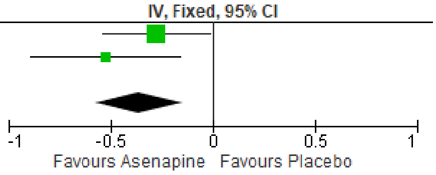

Olanzapine sequentially data only

Shows the forest plots of the standardized mean difference in the changes of symptom score from baseline to week 1, 2, 3, 4, 5, 6 (green squares) with a 95% CI (bars) between olanzapine and placebo. The black diamonds are the summary effect estimates. Only the sequential data are included, meaning studies that in addition to the baseline symptom score presented at least two symptom scores.

Week 1:

	Experimental			Control				Std. Mean Difference
Study or Subgroup	Mean	SD	Total	Mean	SD	Total	Weight	IV, Fixed, 95% CI
Beasley 1996_2 (OLZ)	-3.46	12.5	49	-2.56	12.3	49	6.9%	-0.07 [-0.47, 0.32]
Corrigan 2004 (OLZ)	-8.56	14.6215	93	-4.23	14.6215	85	12.3%	-0.29 [-0.59, 0.00]
Egan 2013 (OLZ)	-7.15	10.3977	45	-4.68	10.4215	78	7.9%	-0.24 [-0.60, 0.13]
Meltzer 2011 (OLZ)	-10.45	20.9	121	-6.82	22.4219	114	16.4%	-0.17 [-0.42, 0.09]
Mosolov 2011 (OLZ)	-3.81	14.1109	34	1.91	20.24	63	6.1%	-0.31 [-0.73, 0.11]
Potkin 2020 (OLZ)	-7.95	9.5934	132	-7.19	8.7647	133	18.5%	-0.08 [-0.32, 0.16]
Schmidt 2012 (OLZ)	-7.5	11.1866	93	-3.84	10.8454	99	13.2%	-0.33 [-0.62, -0.05]
Shen 2008 (OLZ)	-10.2	13.7712	68	-2.55	14.0186	68	9.2%	-0.55 [-0.89, -0.20]
Shen 2014 (OLZ)	-9.7	13.2712	71	-1.62	13.2712	71	9.5%	-0.61 [-0.94, -0.27]
**Total (95% CI)**			**706**			**760**	**100.0%**	**-0.27 [-0.38, -0.17]**
Heterogeneity: Chi² = 10.51, df = 8 (P = 0.23); I² = 24%								
Test for overall effect: Z = 5.16 (P < 0.00001)								

Std. Mean Difference
IV, Fixed, 95% CI

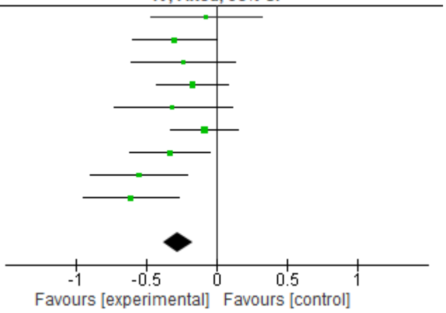

Week 2:

	Experimental			Control				Std. Mean Difference
Study or Subgroup	Mean	SD	Total	Mean	SD	Total	Weight	IV, Fixed, 95% CI
Beasley 1996_2 (OLZ)	-8.1	12.5	47	-2.75	12.3	45	6.4%	-0.43 [-0.84, -0.01]
Corrigan 2004 (OLZ)	-18.14	19.0761	93	-8.56	19.0761	85	12.2%	-0.50 [-0.80, -0.20]
Egan 2013 (OLZ)	-11.42	15.0935	45	-8.01	15.2789	78	8.1%	-0.22 [-0.59, 0.15]
Meltzer 2011 (OLZ)	-15.77	20.9	121	-10.23	22.4219	114	16.6%	-0.26 [-0.51, 0.00]
Mosolov 2011 (OLZ)	-10.72	18.2509	34	4.44	22.4624	63	5.9%	-0.71 [-1.14, -0.28]
Potkin 2020 (OLZ)	-13.56	12.2934	132	-11.41	12.3398	133	18.8%	-0.17 [-0.42, 0.07]
Schmidt 2012 (OLZ)	-13.41	13.6458	93	-4.74	13.7806	99	13.0%	-0.63 [-0.92, -0.34]
Shen 2008 (OLZ)	-10.14	15.4204	68	-2.78	15.6678	68	9.4%	-0.47 [-0.81, -0.13]
Shen 2014 (OLZ)	-9.95	14.3245	71	-1.47	15.1671	71	9.7%	-0.57 [-0.91, -0.24]
**Total (95% CI)**			**704**			**756**	**100.0%**	**-0.40 [-0.51, -0.30]**
Heterogeneity: Chi² = 11.54, df = 8 (P = 0.17); I² = 31%								
Test for overall effect: Z = 7.59 (P < 0.00001)								

Std. Mean Difference
IV, Fixed, 95% CI

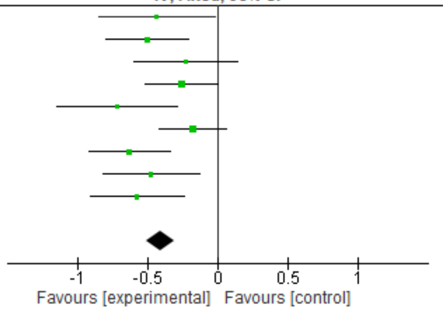

Week 3:

	Experimental			Control				Std. Mean Difference
Study or Subgroup	Mean	SD	Total	Mean	SD	Total	Weight	IV, Fixed, 95% CI
Beasley 1996_2 (OLZ)	-7.03	12.5	43	-2.31	12.3	44	6.2%	-0.38 [-0.80, 0.05]
Corrigan 2004 (OLZ)	-24.18	19.076	93	-13.8	19.076	85	12.3%	-0.54 [-0.84, -0.24]
Egan 2013 (OLZ)	-14.12	15.1605	45	-10.81	17.8402	78	8.2%	-0.19 [-0.56, 0.17]
Meltzer 2011 (OLZ)	-20.88	20.9	121	-11.32	22.4219	114	16.5%	-0.44 [-0.70, -0.18]
Mosolov 2011 (OLZ)	-15.11	21.6911	34	7.12	25.9548	63	5.8%	-0.90 [-1.33, -0.46]
Potkin 2020 (OLZ)	-18.53	13.0976	132	-13.34	14.4734	133	18.8%	-0.37 [-0.62, -0.13]
Schmidt 2012 (OLZ)	-18.43	15.6709	93	-6.81	17.0143	99	13.0%	-0.71 [-1.00, -0.41]
Shen 2008 (OLZ)	-12.24	18.2241	68	-3.06	18.2241	68	9.5%	-0.50 [-0.84, -0.16]
Shen 2014 (OLZ)	-11.9	17.1472	71	-1.7	17.737	71	9.8%	-0.58 [-0.92, -0.25]
**Total (95% CI)**			**700**			**755**	**100.0%**	**-0.50 [-0.60, -0.39]**
Heterogeneity: Chi² = 9.62, df = 8 (P = 0.29); I² = 17%								
Test for overall effect: Z = 9.27 (P < 0.00001)								

Std. Mean Difference
IV, Fixed, 95% CI

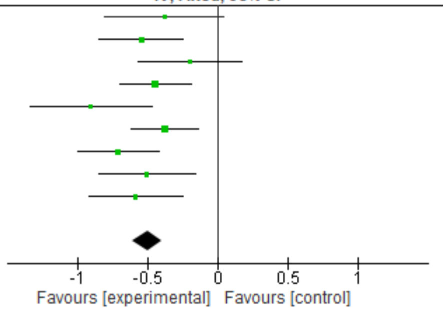

Week 4:

Study or Subgroup	Experimental Mean	SD	Total	Control Mean	SD	Total	Weight	Std. Mean Difference IV, Fixed, 95% CI
Beasley 1996_2 (OLZ)	-7.44	12.5	42	-2.09	12.3	42	6.0%	-0.43 [-0.86, 0.01]
Corrigan 2004 (OLZ)	-30.23	19.076	93	-17.13	19.076	85	12.2%	-0.68 [-0.99, -0.38]
Egan 2013 (OLZ)	-17	19.5209	45	-12.7	19.8715	78	8.3%	-0.22 [-0.58, 0.15]
Meltzer 2011 (OLZ)	-24.59	20.9	121	-12.91	22.4219	114	16.5%	-0.54 [-0.80, -0.28]
Mosolov 2011 (OLZ)	-19.13	22.3325	34	8.16	28.0185	63	5.7%	-1.03 [-1.48, -0.59]
Potkin 2020 (OLZ)	-22.8	14.9359	132	-17.5	14.9923	133	19.0%	-0.35 [-0.60, -0.11]
Schmidt 2012 (OLZ)	-20.74	17.3586	93	-7.41	18.8053	99	13.1%	-0.73 [-1.03, -0.44]
Shen 2008 (OLZ)	-14.16	18.9663	68	-3.74	19.2137	68	9.5%	-0.54 [-0.89, -0.20]
Shen 2014 (OLZ)	-14.2	17.9898	71	-2.23	18.369	71	9.8%	-0.65 [-0.99, -0.32]
**Total (95% CI)**			**699**			**753**	**100.0%**	**-0.55 [-0.66, -0.45]**

Heterogeneity: Chi² = 13.21, df = 8 (P = 0.10); I² = 39%
Test for overall effect: Z = 10.25 (P < 0.00001)

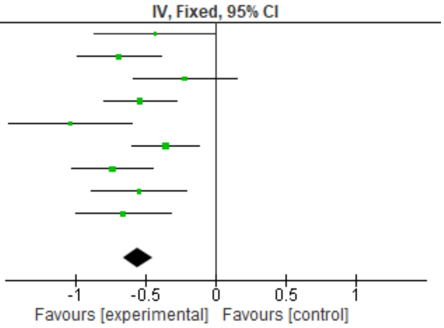

Week 5:

Study or Subgroup	Experimental Mean	SD	Total	Control Mean	SD	Total	Weight	Std. Mean Difference IV, Fixed, 95% CI
Beasley 1996_2 (OLZ)	-12.53	12.5	22	-8.31	12.3	14	3.9%	-0.33 [-1.01, 0.34]
Corrigan 2004 (OLZ)	-35.57	19.076	93	-19.75	19.076	85	19.0%	-0.83 [-1.13, -0.52]
Meltzer 2011 (OLZ)	-27.19	20.9	121	-14.91	22.4219	114	26.2%	-0.57 [-0.83, -0.30]
Schmidt 2012 (OLZ)	-21.87	17.3104	93	-5.94	19.651	99	20.4%	-0.86 [-1.15, -0.56]
Shen 2008 (OLZ)	-16.94	20.1208	68	-3.8	20.1208	68	15.0%	-0.65 [-0.99, -0.30]
Shen 2014 (OLZ)	-16.76	19.1695	71	-2.61	19.2959	71	15.4%	-0.73 [-1.07, -0.39]
**Total (95% CI)**			**468**			**451**	**100.0%**	**-0.70 [-0.84, -0.57]**

Heterogeneity: Chi² = 3.98, df = 5 (P = 0.55); I² = 0%
Test for overall effect: Z = 10.31 (P < 0.00001)

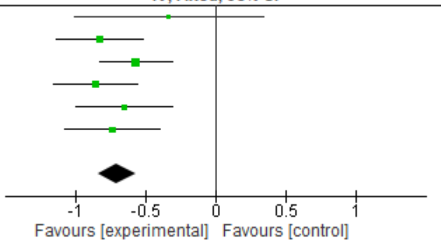

Week 6:

Study or Subgroup	Experimental Mean	SD	Total	Control Mean	SD	Total	Weight	Std. Mean Difference IV, Fixed, 95% CI
Beasley 1996_2 (OLZ)	-15.2	16.1	65	-3.1	17.5	62	12.6%	-0.72 [-1.08, -0.36]
Corrigan 2004 (OLZ)	-31.5	19.076	93	-12.6	19.076	85	16.7%	-0.99 [-1.30, -0.67]
Meltzer 2011 (OLZ)	-28.7	20.9	121	-16	22.4219	114	23.8%	-0.58 [-0.85, -0.32]
Schmidt 2012 (OLZ)	-22.9	18.1301	93	-6.4	20.2977	99	18.6%	-0.85 [-1.15, -0.56]
Shen 2008 (OLZ)	-15.2	20.7805	68	-4.43	20.698	68	13.9%	-0.52 [-0.86, -0.17]
Shen 2014 (OLZ)	-14.7	20.2228	71	-2.7	20.5598	71	14.4%	-0.59 [-0.92, -0.25]
**Total (95% CI)**			**511**			**499**	**100.0%**	**-0.71 [-0.84, -0.58]**

Heterogeneity: Chi² = 6.56, df = 5 (P = 0.26); I² = 24%
Test for overall effect: Z = 10.89 (P < 0.00001)

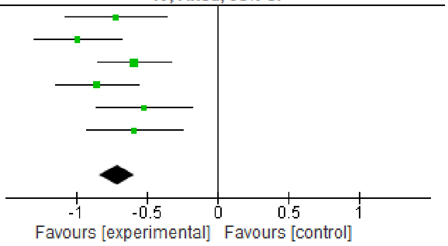

Quetiapine sequentially data only

Shows the forest plots of the standardized mean difference in the changes of symptom score from baseline to week 1, 2, 3, 4, 5, 6 (green squares) with a 95% CI (bars) between quetiapine and placebo. The black diamonds are the summary effect estimates. Only the sequential data are included, meaning studies that in addition to the baseline symptom score presented at least two symptom scores.

Week 1:

Study or Subgroup	Experimental Mean	SD	Total	Control Mean	SD	Total	Weight	Std. Mean Difference IV, Fixed, 95% CI
Borrison 1996 (QTP)	-3.16	10.083	53	-0.7	10.1922	53	15.2%	-0.24 [-0.62, 0.14]
Canuso 2009 (QTP)	-12.88	15.9962	150	-11.6	15.9962	79	29.9%	-0.08 [-0.35, 0.19]
Fabre 1995 (QTP)	-9.01	8.5	8	-5.59	13.2	4	1.5%	-0.31 [-1.52, 0.90]
Potkin 2006 (QTP)	-13.88	17.028	146	-16.38	17.028	68	26.8%	0.15 [-0.14, 0.43]
Small 1997 (QTP)	-2.41	9.9753	92	-0.39	10.0233	92	26.5%	-0.20 [-0.49, 0.09]
**Total (95% CI)**			**449**			**296**	**100.0%**	**-0.08 [-0.23, 0.07]**

Heterogeneity: Chi² = 3.86, df = 4 (P = 0.42); I² = 0%
Test for overall effect: Z = 1.04 (P = 0.30)

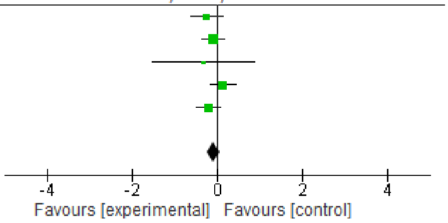

Week 2:

Study or Subgroup	Experimental Mean	SD	Total	Control Mean	SD	Total	Weight	Std. Mean Difference IV, Fixed, 95% CI	Std. Mean Difference IV, Fixed, 95% CI
Borrison 1996 (QTP)	-5.6	14.997	53	-0.5	13.8322	53	16.6%	-0.35 [-0.73, 0.03]	
Canuso 2009 (QTP)	-20.26	14.9351	134	-21.12	14.9351	68	28.7%	0.06 [-0.23, 0.35]	
Fabre 1995 (QTP)	-17.05	8.5	8	-1.22	13.2	4	1.3%	-1.44 [-2.83, -0.05]	
Potkin 2006 (QTP)	-23.8	15.6222	129	-21.38	15.6222	56	24.8%	-0.15 [-0.47, 0.16]	
Small 1997 (QTP)	-4.57	12.2773	92	0.07	12.8528	92	28.7%	-0.37 [-0.66, -0.08]	
**Total (95% CI)**			**416**			**273**	**100.0%**	**-0.20 [-0.36, -0.05]**	

Heterogeneity: Chi² = 7.99, df = 4 (P = 0.09); I² = 50%
Test for overall effect: Z = 2.56 (P = 0.01)

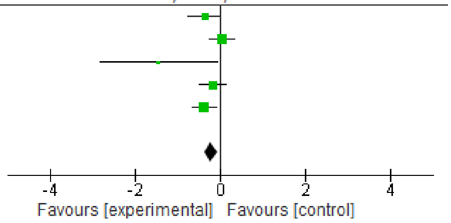

Week 3:

Study or Subgroup	Experimental Mean	SD	Total	Control Mean	SD	Total	Weight	Std. Mean Difference IV, Fixed, 95% CI	Std. Mean Difference IV, Fixed, 95% CI
Borrison 1996 (QTP)	-7	15.2882	53	-1.97	15.179	53	35.7%	-0.33 [-0.71, 0.06]	
Fabre 1995 (QTP)	-20.9	8.5	8	-4.7	13.2	4	2.7%	-1.47 [-2.87, -0.08]	
Small 1997 (QTP)	-6.43	14.6752	92	-0.71	14.1957	92	61.6%	-0.39 [-0.69, -0.10]	
**Total (95% CI)**			**153**			**149**	**100.0%**	**-0.40 [-0.63, -0.17]**	

Heterogeneity: Chi² = 2.40, df = 2 (P = 0.30); I² = 17%
Test for overall effect: Z = 3.42 (P = 0.0006)

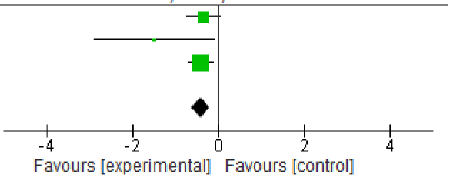

Week 4:

Study or Subgroup	Experimental Mean	SD	Total	Control Mean	SD	Total	Weight	Std. Mean Difference IV, Fixed, 95% CI	Std. Mean Difference IV, Fixed, 95% CI
Borrison 1996 (QTP)	-8.4	15.2882	53	-1.8	15.2882	53	36.7%	-0.43 [-0.81, -0.04]	
Small 1997 (QTP)	-7.71	13.9079	92	-0.51	14.6752	92	63.3%	-0.50 [-0.80, -0.21]	
**Total (95% CI)**			**145**			**145**	**100.0%**	**-0.47 [-0.71, -0.24]**	

Heterogeneity: Chi² = 0.09, df = 1 (P = 0.77); I² = 0%
Test for overall effect: Z = 3.98 (P < 0.0001)

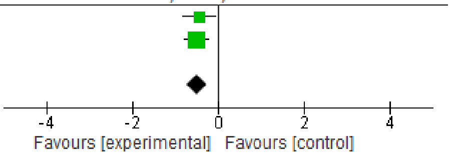

Week 5:

Study or Subgroup	Experimental Mean	SD	Total	Control Mean	SD	Total	Weight	Std. Mean Difference IV, Fixed, 95% CI	Std. Mean Difference IV, Fixed, 95% CI
Borrison 1996 (QTP)	-8.7	16.6715	53	-1.3	16.7443	53	36.7%	-0.44 [-0.83, -0.05]	
Small 1997 (QTP)	-8.02	15.1548	92	-0.32	14.6752	92	63.3%	-0.51 [-0.81, -0.22]	
**Total (95% CI)**			**145**			**145**	**100.0%**	**-0.49 [-0.72, -0.25]**	

Heterogeneity: Chi² = 0.09, df = 1 (P = 0.76); I² = 0%
Test for overall effect: Z = 4.08 (P < 0.0001)

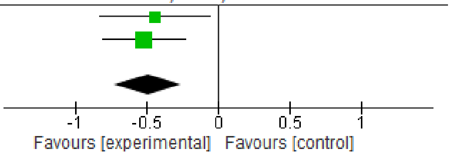

Week 6:

Study or Subgroup	Experimental Mean	SD	Total	Control Mean	SD	Total	Weight	Std. Mean Difference IV, Fixed, 95% CI	Std. Mean Difference IV, Fixed, 95% CI
Borrison 1996 (QTP)	-8.1	17.3995	53	-2.1	17.3995	53	36.9%	-0.34 [-0.73, 0.04]	
Small 1997 (QTP)	-8.7	15.7303	92	-1	15.4426	92	63.1%	-0.49 [-0.79, -0.20]	
**Total (95% CI)**			**145**			**145**	**100.0%**	**-0.44 [-0.67, -0.20]**	

Heterogeneity: Chi² = 0.37, df = 1 (P = 0.54); I² = 0%
Test for overall effect: Z = 3.67 (P = 0.0002)

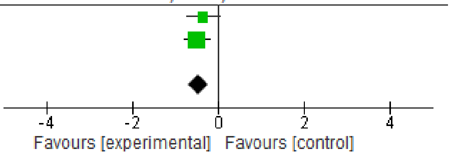

Zotepine sequentially data only

Shows the forest plots of the standardized mean difference in the changes of symptom score from baseline to week 1, 2, 4, 6, 8, 16, 20, 26 (green squares) with a 95% CI (bars) between zotepine and placebo. The black diamonds are the summary effect estimates. Only the sequential data are included, meaning studies that in addition to the baseline symptom score presented at least two symptom scores.

Week 1:

	Experimental			Control				Std. Mean Difference
Study or Subgroup	Mean	SD	Total	Mean	SD	Total	Weight	IV, Fixed, 95% CI
Cooper 2000_1 (ZOT)	-4.6	7.4	53	-1.4	7.9	53	100.0%	-0.42 [-0.80, -0.03]
**Total (95% CI)**			**53**			**53**	**100.0%**	**-0.42 [-0.80, -0.03]**

Heterogeneity: Not applicable
Test for overall effect: Z = 2.11 (P = 0.03)

Std. Mean Difference IV, Fixed, 95% CI

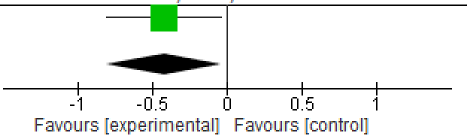

Week 2:

	Experimental			Control				Std. Mean Difference
Study or Subgroup	Mean	SD	Total	Mean	SD	Total	Weight	IV, Fixed, 95% CI
Cooper 2000_1 (ZOT)	-9	10.8	53	-2.4	11.9	53	46.1%	-0.58 [-0.97, -0.19]
Cooper 2000_2 (ZOT)	-3.38	15	61	-0.98	17.1	58	53.9%	-0.15 [-0.51, 0.21]
**Total (95% CI)**			**114**			**111**	**100.0%**	**-0.35 [-0.61, -0.08]**

Heterogeneity: Chi² = 2.51, df = 1 (P = 0.11); I² = 60%
Test for overall effect: Z = 2.57 (P = 0.01)

Std. Mean Difference IV, Fixed, 95% CI

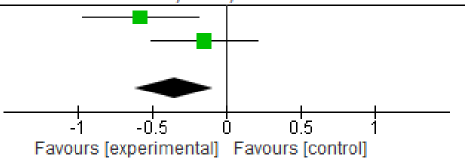

Week 4:

	Experimental			Control				Std. Mean Difference
Study or Subgroup	Mean	SD	Total	Mean	SD	Total	Weight	IV, Fixed, 95% CI
Cooper 2000_1 (ZOT)	-11.8	11.9	53	-4.5	14.5	53	46.2%	-0.55 [-0.93, -0.16]
Cooper 2000_2 (ZOT)	-4.58	15	61	-3.2	17.1	58	53.8%	-0.09 [-0.45, 0.27]
**Total (95% CI)**			**114**			**111**	**100.0%**	**-0.30 [-0.56, -0.03]**

Heterogeneity: Chi² = 2.92, df = 1 (P = 0.09); I² = 66%
Test for overall effect: Z = 2.22 (P = 0.03)

Std. Mean Difference IV, Fixed, 95% CI

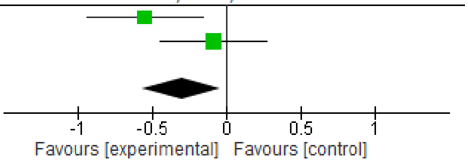

Week 6:

	Experimental			Control				Std. Mean Difference
Study or Subgroup	Mean	SD	Total	Mean	SD	Total	Weight	IV, Fixed, 95% CI
Cooper 2000_1 (ZOT)	-14.8	13.7	53	-3.7	14.9	53	100.0%	-0.77 [-1.17, -0.37]
**Total (95% CI)**			**53**			**53**	**100.0%**	**-0.77 [-1.17, -0.37]**

Heterogeneity: Not applicable
Test for overall effect: Z = 3.82 (P = 0.0001)

Std. Mean Difference IV, Fixed, 95% CI

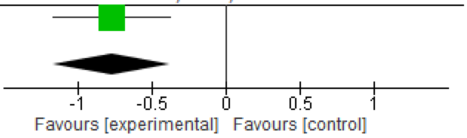

Week 8:

	Experimental			Control				Std. Mean Difference
Study or Subgroup	Mean	SD	Total	Mean	SD	Total	Weight	IV, Random, 95% CI
Cooper 2000_1 (ZOT)	-16.8	14.9	53	-2.9	16.3	53	48.8%	-0.88 [-1.28, -0.48]
Cooper 2000_2 (ZOT)	-6.68	15	61	-1.59	17.1	58	51.2%	-0.31 [-0.68, 0.05]
**Total (95% CI)**			**114**			**111**	**100.0%**	**-0.59 [-1.15, -0.04]**

Heterogeneity: Tau² = 0.12; Chi² = 4.28, df = 1 (P = 0.04); I² = 77%
Test for overall effect: Z = 2.08 (P = 0.04)

Std. Mean Difference IV, Random, 95% CI

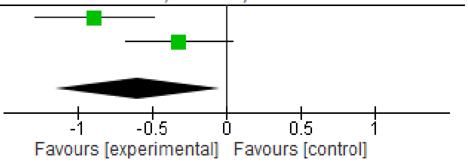

Week 16:

Study or Subgroup	Experimental Mean	Experimental SD	Experimental Total	Control Mean	Control SD	Control Total	Weight	Std. Mean Difference IV, Fixed, 95% CI
Cooper 2000_2 (ZOT)	-5.52	15	61	0.9	17.1	58	100.0%	-0.40 [-0.76, -0.03]
**Total (95% CI)**			**61**			**58**	**100.0%**	**-0.40 [-0.76, -0.03]**

Heterogeneity: Not applicable
Test for overall effect: Z = 2.14 (P = 0.03)

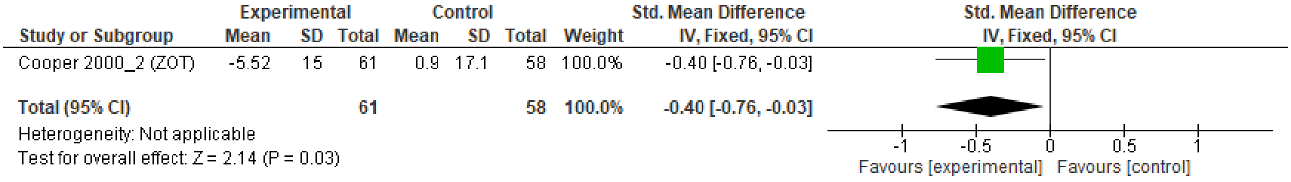

Week 20:

Study or Subgroup	Experimental Mean	Experimental SD	Experimental Total	Control Mean	Control SD	Control Total	Weight	Std. Mean Difference IV, Fixed, 95% CI
Cooper 2000_2 (ZOT)	-5.71	15	61	1.27	17.1	58	100.0%	-0.43 [-0.80, -0.07]
**Total (95% CI)**			**61**			**58**	**100.0%**	**-0.43 [-0.80, -0.07]**

Heterogeneity: Not applicable
Test for overall effect: Z = 2.33 (P = 0.02)

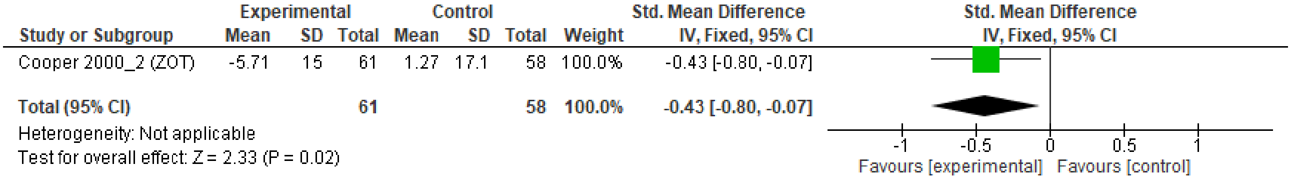

Week 26:

Study or Subgroup	Experimental Mean	Experimental SD	Experimental Total	Control Mean	Control SD	Control Total	Weight	Std. Mean Difference IV, Fixed, 95% CI
Cooper 2000_2 (ZOT)	-5.7	15	61	1.8	17.1	58	100.0%	-0.46 [-0.83, -0.10]
**Total (95% CI)**			**61**			**58**	**100.0%**	**-0.46 [-0.83, -0.10]**

Heterogeneity: Not applicable
Test for overall effect: Z = 2.50 (P = 0.01)

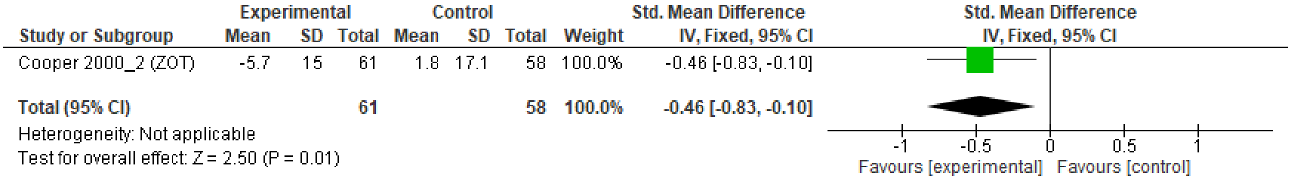

Supplemental 7 – Sensitivity analyses, enriched vs non-enriched

Illustrates the summary of standardized mean difference in the changes of symptom score from baseline to week 1, 2, 3, 4, 5, 6 with a 95% CI between placebo and sublingual asenapine, olanzapine, quetiapine and quetiapine extended release respectively. CHI^2 expresses the heterogeneity across studies, in each meta-analysis. The meta-analyses were made for the enriched population and the non-enriched population respectively. ([n=number of participants])

Asenapine, enriched and non-enriched

	Enriched	Non-enriched
Studies	Potkin 2007<br>Kinoshita 2016<br>Landbloom 2017	Kane 2010
Week 1	-0.33 [-0.70, 0.03]<br>P(CHI^2) = NA<br>(n=118)	-0.18 [-0.44, 0.08]<br>P(CHI^2) = NA<br>(n=227)
Week 2	-0.36 [-0.73, 0.00]<br>P(CHI^2) = NA<br>(n=118)	-0.20 [-0.46, 0.06]<br>P(CHI^2) = NA<br>(n=227)
Week 3	-0.62 [-0.99, -0.25]<br>P(CHI^2) = NA<br>(n=118)	-0.20 [-0.46, 0.06]<br>P(CHI^2) = NA<br>(n=227)
Week 4	-0.52 [-0.89, -0.16]<br>P(CHI^2) = NA<br>(n=118)	-0.17 [-0.43, 0.10]<br>P(CHI^2) = NA<br>(n=227)
Week 5	-0.62 [-0.99, -0.25]<br>P(CHI^2) = NA<br>(n=118)	-0.25 [-0.51, 0.01]<br>P(CHI^2) = NA<br>(n=227)
Week 6	-0.53 [-0.68, -0.37]<br>P(CHI^2) = 0.17<br>(n=680)	-0.29 [-0.55, -0.02]<br>P(CHI^2) = NA<br>(n=227)

Olanzapine, enriched and non-enriched

	Enriched	Non-enriched
Studies	Bugarski-kirola 2014<br>Davidson 2007<br>Egan 2013<br>Kane 2007<br>Landbloom 2017<br>Lecrubier 2006<br>Litman 2014<br>Marder 2007<br>Potkin 2020<br>Schmidt 2012<br>Shen 2014	Beasley 1996 (March)<br>Beasley 1996 (July)<br>Corrigan 2004<br>Kinon 2011<br>Meltzer 2011<br>Mosolov 2011<br>Shen 2008

Week 1	-0.28 [-0.42, -0.13]<br>$P(CHI^2) = 0.10$<br>(n=722)	-0.27 [-0.42, -0.13]<br>$P(CHI^2) = 0.38$<br>(n=744)
Week 2	-0.38 [-0.53, -0.23]<br>$P(CHI^2) = 0.06$<br>(n=722)	-0.43 [-0.58, -0.28]<br>$P(CHI^2) = 0.45$<br>(n=738)
Week 3	-0.47 [-0.62, -0.32]<br>$P(CHI^2) = 0.13$<br>(n=722)	-0.52 [-0.67, -0.37]<br>$P(CHI^2) = 0.45$<br>(n=733)
Week 4	-0.48 [-0.69, -0.26]<br>$P(CHI^2) = 0.04$<br>(n=925)	-0.55 [-0.68, -0.41]<br>$P(CHI^2) = 0.10$<br>(n=914)
Week 5	-0.80 [-1.03, -0.58]<br>$P(CHI^2) = 0.59$<br>(n=334)	-0.65 [-0.81, -0.48]<br>$P(CHI^2) = 0.47$<br>(n=585)
Week 6	-0.65 [-0.77, -0.53]<br>P(CHI2) = 0.20<br>(n=1188)	-0.68 [-0.84, -0.53]<br>$P(CHI^2) = 0.23$<br>(n=704)
6 months	-0.12 [-0.53, 0.29]<br>$P(CHI^2)$ = NA<br>(n=104)	-

Quetiapine, enriched and non-enriched

	Enriched	Non-enriched
Studies	Canuso 2009<br>Kahn 2007<br>Lindenmayer 2008	Arvanitis 1997<br>Borison 1996<br>Fabre 1995<br>Potkin 2006<br>Small 1997
Week 1	-0.08 [-0.35, 0.19]<br>$P(CHI^2)$ = NA<br>(n=229)	-0.08 [-0.26, 0.10]<br>$P(CHI^2) = 0.28$<br>(n=516)
Week 2	0.06 [-0.23, 0.35]<br>$P(CHI^2)$ = NA<br>(n=202)	-0.31 [-0.49, -0.12]<br>$P(CHI^2) = 0.30$<br>(n=487)
Week 3	-	-0.40 [-0.63, -0.17]<br>$P(CHI^2) = 0.30$<br>(n=302)
Week 4	-	-0.47 [-0.71, -0.24]<br>$P(CHI^2) = 0.77$<br>(n=290)

Week 5	-	-0.49 [-0.72, -0.25]<br>P(CHI^2) = 0.76<br>(n=290)
Week 6	-0.27 [-0.47, -0.07]<br>P(CHI^2) = 0.18<br>(n=392)	-0.46 [-0.66, -0.26]<br>P(CHI^2) = 0.75<br>(n=394)

Quetiapine XR, enriched and non-enriched

	Enriched	Non-enriched
Studies	Kahn 2007<br>Lindenmayer 2008	Loebel 2013
Week 1	-	-0.24 [-0.50, 0.01]<br>P(CHI2) = NA<br>(n=236)
Week 2	-	-0.46 [-0.72, -0.20]<br>P(CHI^2) = NA<br>(n=236)
Week 3	-	-0.62 [-0.88, -0.36]<br>P(CHI^2) = NA<br>(n=236)
Week 4	-	-0.76 [-1.02, -0.49]<br>P(CHI^2) = NA<br>(n=236)
Week 5	-	-0.86 [-1.12, -0.59]<br>P(CHI^2) = NA<br>(n=236)
Week 6	-0.41 [-0.61, -0.21]<br>P(CHI^2) = 0.78<br>(n=395)	-0.89 [-1.16, -0.62]<br>P(CHI^2) = NA<br>(n=236)

*NA=non-applied

Supplemental 8 – Sensitivity analyses, withdrawal of placebo-responders

Illustrates the summary of standardized mean difference in the changes of symptom score from baseline to week 1, 2, 3, 4, 5, 6 with a 95% CI between placebo and olanzapine, quetiapine and zotepine respectively. CHI^2 expresses the heterogeneity across studies in each meta-analysis. The meta-analyses were made by excluding studies reporting withdrawal of placebo-responders before randomization. ([n=number of participants])

Olanzapine, without studies reporting withdrawal of placebo-responders

	Placebo responders withdrawn
Studies removed	Beasley 1996_1<br>Beasley 1996_2
Week 1	-0.30 [-0.42, -0.18]<br>P(CHI^2) = 0.17<br>(n=1012)
Week 2	-0.43 [-0.55, -0.30]<br>P(CHI^2) = 0.13<br>(n=1012)
Week 3	-0.50 [-0.63, -0.38]<br>P(CHI^2) = 0.49<br>(n=1012)
Week 4	-0.47 [-0.58, -0.35]<br>P(CHI^2) = 0.13<br>(n=1152)
Week 5	-0.61 [-0.76, -0.46]<br>P(CHI^2) = 0.14<br>(n=747)
Week 6	-0.62 [-0.72, -0.52]<br>P(CHI^2) = 0.30<br>(n=1601)

Quetiapine, without studies reporting withdrawal of placebo-responders

	Placebo responders withdrawn
Studies removed	Arvanitis 1997
Week 6	-0.26 [-0.41, -0.11]<br>P(CHI^2) = 0.55<br>(n=686)

Zotepine, without studies reporting withdrawal of placebo-responders

	Placebo responders withdrawn
Studies removed	Moller 2004
Week 8	-0.59 [-1.15, -0.03]<br>$P(CHI^2) = 0.04$<br>(n=225)

References

1. Page, M.J.; McKenzie, J.E.; Bossuyt, P.M.; Boutron, I.; Hoffmann, T.C.; Mulrow, C.D.; Al., E. The PRISMA 2020 Statement: An Updated Guideline for Reporting Systematic Reviews. *BMJ* **2021**, *372*, n71, doi:10.1136/bmj.n71.
2. Potkin, S.G.; Cohen, M.; Panagides, J. Efficacy and Tolerability of Asenapine in Acute Schizophrenia: A Placebo- and Risperidone-Controlled Trial. *J. Clin. Psychiatry* **2007**, *68*, 1492–1500.
3. Kane, J.M.; Cohen, M.; Zhao, J.; Alphs, L.; Panagides, J. Efficacy and Safety of Asenapine in a Placebo- and Haloperidol-Controlled Trial in Patients with Acute Exacerbation of Schizophrenia. *J. Clin. Psychopharmacol.* **2010**, *30*, 106–115, doi:10.1097/JCP.0b013e3181d35d6b.
4. Kinoshita, T.; Bai, Y.-M.; Kim, J.-H.; Miyake, M.; Oshima, N. Efficacy and Safety of Asenapine in Asian Patients with an Acute Exacerbation of Schizophrenia: A Multicentre, Randomized, Double-Blind, 6-Week, Placebo-Controlled Study. *Psychopharmacology (Berl).* **2016**, *233*, 2663–2674, doi:10.1007/s00213-016-4295-9.
5. Landbloom, R.; Mackle, M.; Wu, X.; Kelly, L.; Snow-Adami, L.; McIntyre, R.S.; Mathews, M.; Hundt, C. Asenapine for the Treatment of Adults with an Acute Exacerbation of Schizophrenia: Results from a Randomized, Double-Blind, Fixed-Dose, Placebo-Controlled Trial with Olanzapine as an Active Control. *CNS Spectr.* **2017**, *22*, 333–341, doi:10.1017/S1092852916000377.
6. Citrome, L.; Walling, D.P.; Zeni, C.M.; Starling, B.R.; Terahara, T.; Kuriki, M.; Park, A.S.; Komaroff, M. Efficacy and Safety of HP-3070, an Asenapine Transdermal System, in Patients With Schizophrenia: A Phase 3, Randomized, Placebo-Controlled Study. *J. Clin. Psychiatry* **2020**, *82*, 20m13602, doi:10.4088/JCP.20m13602.
7. Lecrubier, Y.; Quintin, P.; Bouhassira, M.; Perrin, E.; Lancrenon, S. The Treatment of Negative Symptoms and Deficit States of Chronic Schizophrenia: Olanzapine Compared to Amisulpride and Placebo in a 6-Month Double-Blind Controlled Clinical Trial. *Acta Psychiatr. Scand.* **2006**, *114*, 319–327, doi:10.1111/j.1600-0447.2006.00887.x.
8. Meltzer, H.Y.; Cucchiaro, J.; Silva, R.; Ogasa, M.; Phillips, D.; Xu, J.; Kalali, A.H.; Schweizer, E.; Pikalov, A.; Loebel, A. Lurasidone in the Treatment of Schizophrenia: A Randomized, Double-Blind, Placebo- and Olanzapine-Controlled Study. *Am. J. Psychiatry* **2011**, *168*, 957–967, doi:10.1176/appi.ajp.2011.10060907.
9. Beasley, C.M.J.; Tollefson, G.; Tran, P.; Satterlee, W.; Sanger, T.; Hamilton, S. Olanzapine versus Placebo and Haloperidol: Acute Phase Results of the North American Double-Blind Olanzapine Trial. *Neuropsychopharmacology* **1996**, *14*, 111–123, doi:10.1016/0893-133X(95)00069-P.
10. Beasley, C.M.J.; Sanger, T.; Satterlee, W.; Tollefson, G.; Tran, P. Olanzapine versus Placebo: Results of a Double-Blind, Fixed Dose Olanzapine Trial. *Psychopharmacology (Berl).* **1996**, *124*, 159–167, doi:http://dx.doi.org/10.1007/BF02245617.
11. Corrigan, M.H.; Gallen, C.C.; Bonura, M.L.; Merchant, K.M. Effectiveness of the Selective D4 Antagonist Sonepiprazole in Schizophrenia: A Placebo-Controlled Trial. *Biol. Psychiatry* **2004**, *55*, 445–451, doi:10.1016/j.biopsych.2003.10.004.
12. Marder, S.R.; Kramer, M.; Ford, L.; Eerdekens, E.; Lim, P.; Eerdekens, M.; Lowy, A. Efficacy and Safety of Paliperidone Extended-Release Tablets: Results of a 6-Week, Randomized, Placebo-Controlled Study. *Biol. Psychiatry* **2007**, *62*, 1363–1370, doi:10.1016/j.biopsych.2007.01.017.
13. Schmidt, M.E.; Kent, J.M.; Daly, E.; Janssens, L.; Van Osselaer, N.; Husken, G.; Anghelescu, I.-G.; Van Nueten, L. A Double-Blind, Randomized, Placebo-Controlled Study with JNJ-37822681, a Novel, Highly Selective, Fast Dissociating D(2) Receptor Antagonist in the Treatment of Acute Exacerbation of Schizophrenia. *Eur. Neuropsychopharmacol.* **2012**, *22*, 721–733, doi:10.1016/j.euroneuro.2012.02.007.
14. Bugarski-Kirola, D.; Wang, A.; Abi-Saab, D.; Blattler, T. A Phase II/III Trial of Bitopertin Monotherapy Compared with Placebo in Patients with an Acute Exacerbation of Schizophrenia - Results from the CandleLyte Study. *Eur. Neuropsychopharmacol.* **2014**, *24*, 1024–1036, doi:10.1016/j.euroneuro.2014.03.007.

15. Shen, J.H.Q.; Zhao, Y.; Rosenzweig-Lipson, S.; Popp, D.; Williams, J.B.W.; Giller, E.; Detke, M.J.; Kane, J.M. A 6-Week Randomized, Double-Blind, Placebo-Controlled, Comparator Referenced Trial of Vabicaserin in Acute Schizophrenia. *J. Psychiatr. Res.* **2014**, *53*, 14–22, doi:10.1016/j.jpsychires.2014.02.012.
16. Davidson, M.; Emsley, R.; Kramer, M.; Ford, L.; Pan, G.; Lim, P.; Eerdekens, M. Efficacy, Safety and Early Response of Paliperidone Extended-Release Tablets (Paliperidone ER): Results of a 6-Week, Randomized, Placebo-Controlled Study. *Schizophr. Res.* **2007**, *93*, 117–130, doi:10.1016/j.schres.2007.03.003.
17. Kane, J.; Canas, F.; Kramer, M.; Ford, L.; Gassmann-Mayer, C.; Lim, P.; Eerdekens, M. Treatment of Schizophrenia with Paliperidone Extended-Release Tablets: A 6-Week Placebo-Controlled Trial. *Schizophr. Res.* **2007**, *90*, 147–161, doi:10.1016/j.schres.2006.09.012.
18. Potkin, S.G.; Kunovac, J.; Silverman, B.L.; Simmons, A.; Jiang, Y.; DiPetrillo, L.; McDonnell, D. Efficacy and Safety of a Combination of Olanzapine and Samidorphan in Adult Patients with an Acute Exacerbation of Schizophrenia: Outcomes from the Randomized, Phase 3 ENLIGHTEN-1 Study. *J. Clin. Psychiatry* **2020**, *81*, 19m12769, doi:10.4088/JCP.19m12769.
19. Egan, M.F.; Zhao, X.; Smith, A.; Troyer, M.D.; Uebele, V.N.; Pidkorytov, V.; Cox, K.; Murphy, M.; Snavely, D.; Lines, C.; et al. Randomized Controlled Study of the T-Type Calcium Channel Antagonist MK-8998 for the Treatment of Acute Psychosis in Patients with Schizophrenia. *Hum. Psychopharmacol.* **2013**, *28*, 124–133, doi:10.1002/hup.2289.
20. Kinon, B.J.; Zhang, L.; Millen, B.A.; Osuntokun, O.O.; Williams, J.E.; Kollack-Walker, S.; Jackson, K.; Kryzhanovskaya, L.; Jarkova, N. A Multicenter, Inpatient, Phase 2, Double-Blind, Placebo-Controlled Dose-Ranging Study of LY2140023 Monohydrate in Patients with DSM-IV Schizophrenia. *J. Clin. Psychopharmacol.* **2011**, *31*, 349–355, doi:10.1097/JCP.0b013e318218dcd5.
21. Litman, R.E.; Smith, M.A.; Desai, D.G.; Simpson, T.; Sweitzer, D.; Kanes, S.J. The Selective Neurokinin 3 Antagonist AZD2624 Does Not Improve Symptoms or Cognition in Schizophrenia: A Proof-of-Principle Study. *J. Clin. Psychopharmacol.* **2014**, *34*, 199–204, doi:10.1097/JCP.0000000000000071.
22. Mosolov, S.N.; Smulevich, A.B.; Neznanov, N.G.; Tochilov, V.A.; Andreev, B. V; Avedisova, A.S.; Bardenstein, L.M.; Gurovich, Iy.; Reznik, A.M.; Zharkova, N.B.; et al. MGlu2/3 Agonists - A New Approach to the Treatment of Schizophrenia: Results of a Randomized Double-Blind Trial. *Neurosci. Behav. Physiol.* **2011**, *41*, 559-566, doi:10.1007/s11055-011-9455-0.
23. Shen, J.; Kobak, K.A.; Zhao, Y.; Alexander, M.M.; Kane, J.M. Use of Remote Centralized Raters via Live 2-Way Video in a Multicenter Clinical Trial for Schizophrenia. *J. Clin. Psychopharmacol.* **2008**, *28*, 691–693, doi:http://dx.doi.org/10.1097/JCP.0b013e31818c9ba3.
24. Lauriello, J.; Lambert, T.; Andersen, S.; Lin, D.; Taylor, C.C.; McDonnell, D. An 8-Week, Double-Blind, Randomized, Placebo-Controlled Study of Olanzapine Long-Acting Injection in Acutely Ill Patients with Schizophrenia. *J. Clin. Psychiatry* **2008**, *69*, 790–799.
25. Fabre, L.F.J.; Arvanitis, L.; Pultz, J.; Jones, V.M.; Malick, J.B.; Slotnick, V.B. ICI 204,636, a Novel, Atypical Antipsychotic: Early Indication of Safety and Efficacy in Patients with Chronic and Subchronic Schizophrenia. *Clin. Ther.* **1995**, *17*, 366–378.
26. Borison, R.L.; Arvanitis, L.A.; Miller, B.G. ICI 204,636, an Atypical Antipsychotic: Efficacy and Safety in a Multicenter, Placebo-Controlled Trial in Patients with Schizophrenia. U.S. SEROQUEL Study Group. *J. Clin. Psychopharmacol.* **1996**, *16*, 158–169.
27. Arvanitis, L.A.; Miller, B.G. Multiple Fixed Doses of "Seroquel" (Quetiapine) in Patients with Acute Exacerbation of Schizophrenia: A Comparison with Haloperidol and Placebo. The Seroquel Trial 13 Study Group. *Biol. Psychiatry* **1997**, *42*, 233–246.
28. Small, J.G.; Hirsch, S.R.; Arvanitis, L.A.; Miller, B.G.; Link, C.G. Quetiapine in Patients with Schizophrenia. A High- and Low-Dose Double-Blind Comparison with Placebo. Seroquel Study Group. *Arch. Gen. Psychiatry* **1997**, *54*, 549–557.
29. Potkin, S.G.; Gharabawi, G.M.; Greenspan, A.J.; Mahmoud, R.; Kosik-Gonzalez, C.; Rupnow, M.F.T.; Bossie, C.A.; Davidson, M.; Burtea, V.; Zhu, Y.; et al. A Double-Blind Comparison of Risperidone,

Quetiapine and Placebo in Patients with Schizophrenia Experiencing an Acute Exacerbation Requiring Hospitalization. *Schizophr. Res.* **2006**, *85*, 254–265, doi:10.1016/j.schres.2006.03.027.
30. Canuso, C.M.; Dirks, B.; Carothers, J.; Kosik-Gonzalez, C.; Bossie, C.A.; Zhu, Y.; Damaraju, C. V; Kalali, A.H.; Mahmoud, R. Randomized, Double-Blind, Placebo-Controlled Study of Paliperidone Extended-Release and Quetiapine in Inpatients with Recently Exacerbated Schizophrenia. *Am. J. Psychiatry* **2009**, *166*, 691–701, doi:10.1176/appi.ajp.2009.08040613.
31. Loebel, A.; Cucchiaro, J.; Sarma, K.; Xu, L.; Hsu, C.; Kalali, A.H.; Pikalov, A.; Potkin, S.G. Efficacy and Safety of Lurasidone 80 Mg/Day and 160 Mg/Day in the Treatment of Schizophrenia: A Randomized, Double-Blind, Placebo- and Active-Controlled Trial. *Schizophr. Res.* **2013**, *145*, 101–109, doi:10.1016/j.schres.2013.01.009.
32. Kahn, R.S.; Schulz, S.C.; Palazov, V.D.; Reyes, E.B.; Brecher, M.; Svensson, O.; Andersson, H.M.; Meulien, D. Efficacy and Tolerability of Once-Daily Extended Release Quetiapine Fumarate in Acute Schizophrenia: A Randomized, Double-Blind, Placebo-Controlled Study. *J. Clin. Psychiatry* **2007**, *68*, 832–842.
33. Lindenmayer, J.-P.; Brown, D.; Liu, S.; Brecher, M.; Meulien, D. The Efficacy and Tolerability of Once-Daily Extended Release Quetiapine Fumarate in Hospitalized Patients with Acute Schizophrenia: A 6-Week Randomized, Double-Blind, Placebo-Controlled Study. *Psychopharmacol. Bull.* **2008**, *41*, 11–35.
34. Cooper, S.J.; Tweed, J.; Raniwalla, J.; Butler, A.; Welch, C. A Placebo-Controlled Comparison of Zotepine versus Chlorpromazine in Patients with Acute Exacerbation of Schizophrenia. *Acta Psychiatr. Scand.* **2000**, *101*, 218–225.
35. Cooper, S.J.; Butler, A.; Tweed, J.; Welch, C.; Raniwalla, J. Zotepine in the Prevention of Recurrence: A Randomised, Double-Blind, Placebo-Controlled Study for Chronic Schizophrenia. *Psychopharmacology (Berl).* **2000**, *150*, 237–243.
36. Moller, H.J.; Riedel, M.; Muller, N.; Fischer, W.; Kohnen, R. Zotepine versus Placebo in the Treatment of Schizophrenic Patients with Stable Primary Negative Symptoms: A Randomized Double-Blind Multicenter Trial. *Pharmacopsychiatry* **2004**, *37*, 270–278, doi:10.1055/s-2004-832683.
37. Sterne, J.; Savović, J.; Page, M.; Elbers, R.; Blencowe, N.; Boutron, I.; Cates, C.; Cheng, H.-Y.; Corbett, M.; Eldridge, S.; et al. RoB 2: A Revised Tool for Assessing Risk of Bias in Randomised Trials. *BMJ* **2019**, l4898, doi:doi: 10.1136/bmj.l4898.
